# Switch from canonical to recombination-dependent DNA synthesis by M-CDK phosphorylation of DNA Polα

**DOI:** 10.1038/s44318-026-00919-5

**Published:** 2026-10-10

**Authors:** Juan de Dios Barba Tena, Riccardo Montecchi, Maxime Lambert, Thomas Defrance, Elisabeth Gueydon, Julie Petit, Marjorie Drac, Noémie Legoff, Michela Galli, Isabelle Goiffon, Thierry Gostan, Khadija El Koulali, Serge Urbach, David Toczyski, Nicolas Talarek, Etienne Schwob

**Affiliations:** 1 IGMM, CNRS, Univ Montpellier, Montpellier, France; 2 MDC, BioCampus, IGMM, CNRS, Univ Montpellier, Montpellier, France; 3 SERANAD, IGMM, CNRS, Univ Montpellier, Montpellier, France; 4 BCM, Univ. Montpellier, CNRS, INSERM, Montpellier, France; 5 IGF, Univ. Montpellier, CNRS, INSERM, Montpellier, France; 6 Dept Biochemistry & Biophysics, UCSF, San Francisco, CA USA; 7 Present Address: Dortmund Life Science Center, TU Dortmund, Dortmund, Germany; 8 Present Address: IDgenX, Montpellier, France

**Keywords:** Cell Cycle, DNA Replication, Recombination & Repair

## Abstract

Cells preserve genome stability while occasionally generating genetic diversity through mutations and chromosomal rearrangements, but how these opposing outcomes are balanced remains unclear. Because late-replicating regions evolve more rapidly, we investigated the consequences of reduced origin licensing and lengthened S phase in yeast. Under these conditions, cells progress slowly through S phase without checkpoint activation, and enter mitosis with under-replicated DNA. We show that mitotic cyclin-dependent kinase (M-CDK) phosphorylates DNA polymerase α (Pol α), displacing it from replication forks and triggering fork collapse at mitotic entry. The resulting DNA damage response provides a window for mitotic DNA synthesis (MiDAS), enabling completion of replication through error-prone, recombination-dependent mechanisms resembling break-induced replication (BIR) and Fork Stalling & Template Switching (FoSTeS). These findings identify Pol α as a critical M-CDK target that promotes the transition from canonical replication to recombination-driven DNA synthesis when cells enter mitosis with incompletely-duplicated chromosomes. This mechanism offers a potential explanation for the accelerated evolution of late-replicating genomic regions.

## Synopsis

Under-licensing of replication origins fosters genome instability via late replication undetected by S-phase checkpoints. Phosphorylation of DNA Pol**α** by M-CDK is key for allowing the completion of DNA synthesis in mitosis using recombination-dependent mechanisms.

- Replication origin under-licensing extends S phase and leads to mitotic entry before S-phase completion.
- M-CDKs phosphorylate and evict DNA Pol**α** from CMG, activating the DNA damage response and providing time for replication completion by BIR/MiDAS at the expense of genome stability.
- Polα phosphorylation is dispensable for canonical DNA replication, but essential in cells entering mitosis with under-replicated DNA.
- Polα eviction and switch to BIR and to fork stalling & template switching may explain the faster evolution of late-replicating loci.

## Abstract (web summary)

A mechanism ensuring completion of DNA synthesis during mitosis upon replication origins under-licensing may be responsible for accelerated evolution of late-replicating regions.

## Graphical abstract

**Figure:**
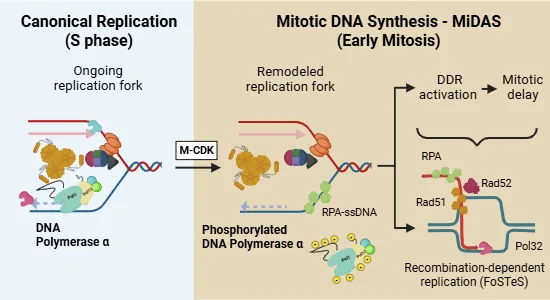

## Introduction

Genomes are the most stable constituents of living organisms. They define species and are perpetuated in the progeny through countless cell divisions. This requires a high fidelity in the processes that duplicate and segregate chromosomes during the S and M phases of the cell cycle. However, cells adapt to their environment, and species evolve by acquiring mutations (SNV, Single Nucleotide Variant) and/or chromosome structural variations (SV). Interestingly, mutation rates are not uniform along the genome (Schuster-Böckler and Lehner, 2012; Wolfe et al, 1989) and increase in conditions of stress and in cancer (Galhardo et al, 2007; Loeb et al, 2008; Radman, 1975; Shor et al, 2013). Genomes are organized in compartments and topologically-associating domains that duplicate at specific times during S phase (Pope et al, 2014; Raghuraman et al, 2001). Later-replicating regions evolve faster from bacteria to human (Dillon et al, 2018; Lang and Murray, 2011; Stamatoyannopoulos et al, 2009) to an extent that genes with adaptive value are often located within late-replicating regions or near telomeres (Cornejo-Páramo et al, 2024; Woo and Li, 2012). The mechanisms underlying the higher instability of late-replicating regions are not fully understood.

Cells ensure that DNA replication is completed in time by preparing (licensing) many more origins than needed to duplicate the genome entirely before mitosis, and by monitoring DNA replication to delay the onset of mitosis until DNA replication is complete (Bell and Labib, 2016). Origin licensing entails binding of the origin-recognition complex (Orc1-6) onto selected sites and recruitment in G_1_, by the Cdc6 and Cdt1 licensing factors, of inactive Mcm2-7 helicase dimers (Costa and Diffley, 2022). The MCM helicase is then activated throughout S phase by Dbf4-dependent (DDK) and S-phase cyclin-dependent (S-CDK) kinases that recruit additional factors and activate the Cdc45-MCM-GINS (CMG) helicase, generating bidirectional replication forks by successive steps that have been reconstituted in vitro (Yeeles et al, 2015). Still, many licensed origins do not fire, i.e., remain dormant during S phase, either because they fail to recruit limiting firing factors, or because they are inactivated by an incoming replication fork (Mantiero et al, 2011; Vujcic et al, 1999). Dormant origins become important for genome stability when replication forks are slowed down or stalled (Ge et al, 2007; Ibarra et al, 2008; Yekezare et al, 2013). Thus, extensive origin licensing in G_1_ is key to ensure the timely duplication of chromosomes. Besides building an excess of origins, cells can also delay the onset of mitosis when replication is ongoing (Eykelenboom et al, 2013; Lemmens et al, 2018; Magiera et al, 2014) or upon fork slowing/stalling when DNA polymerases and CMG helicase uncouple, which generates stretches of primed ssDNA that activate the DDR (Byun et al, 2005; Saldivar et al, 2017; Taylor and Yeeles, 2018). Whereas stalled forks generate substantial amounts of RPA-coated ssDNA that recruits and activates ATR-ATRIP and put a strong hold on mitotic entry, it has been shown that normally progressing forks are sensed by ATR-ETAA1, not ATR-TOPBP1, and do not lead to hyper-phosphorylation of the DDR effectors CHK1/Rad53 but nevertheless delay mitotic entry (Bastos de Oliveira et al, 2015; Koundrioukoff et al, 2013; Saldivar et al, 2018). Thus, the DNA replication checkpoint (DRC) and DNA damage response (DDR) provide extra time for cells to complete DNA synthesis or resolve/restart stalled forks.

However, DRC and DDR are not failsafe mechanisms. Indeed, the existence of unreplicated regions at mitotic entry may represent a prominent way by which genomes acquire new features at a slow pace during evolution or cataclysmically in cancer cells (Blumenfeld et al, 2017; Crasta et al, 2012; Simovic-Lorenz and Ernst, 2025). For example, common fragile sites (CFS) that replicate late due to a paucity in active origins are hotspots of recombination and copy-number variations (Glover et al, 2017; Le Beau et al, 1998; Letessier et al, 2011). Consistently, precocious activation of CDKs in G_1_, which is common in cancer cells, impairs origin licensing, extends S phase and causes genome instability in yeast and human cells (Ekholm-Reed et al, 2004; Lengronne & Schwob, 2002; Tanaka and Diffley, 2002; Teixeira et al, 2015). Interestingly, the strength of the DRC depends on the number of active forks, which decreases in late S and may be insufficient to prevent mitotic entry in case of origin under-licensing (Lengronne and Schwob, 2002; Mocanu et al, 2022; Shimada et al, 2002). Mitotic DNA synthesis (MiDAS) has been detected at CFS and other late-replicating loci, indicating that cells may enter mitosis with incompletely-duplicated chromosomes and then finish DNA synthesis using DNA recombination-based, break-induced replication (BIR)-like mechanisms that generate genome instability (Macheret et al, 2020; Minocherhomji et al, 2015; Mocanu et al, 2022).

Here, we investigate how origin under-licensing and late DNA replication promote MiDAS and genome instability in yeast. We uncover a prominent role for mitotic DNA Polα phosphorylation in the switch from canonical to recombination-dependent replication. Cell-cycle regulated phosphorylation of DNA Polα is conserved from yeast to human, but its role remained enigmatic (Desdouets et al, 1998; Foiani et al, 1995; Nasheuer et al, 1991; Voitenleitner et al, 1997). We show here that phosphorylation of DNA Polα at mitotic entry causes its eviction from the CMG helicase, fork collapse and production of RPA-coated ssDNA, which then triggers a potent DDR. This both halts canonical DNA synthesis and provides time for cells to complete replication using a Pol32-dependent BIR-related mechanism, such as fork stalling and template switching (FoSTeS) or microhomologies/microsatellite-induced Replication (MMIR) (Lee et al, 2007; Payen et al, 2008). This allows cell survival but also creates genome diversity that sustains adaptation and evolution. We propose that this mechanism underlies the faster evolution of late-replicating loci and may play a role in the genome instability of cancer cells.

## Results

### Replication origin under-licensing extends the S phase and alters the replication program

**Figure 1:**
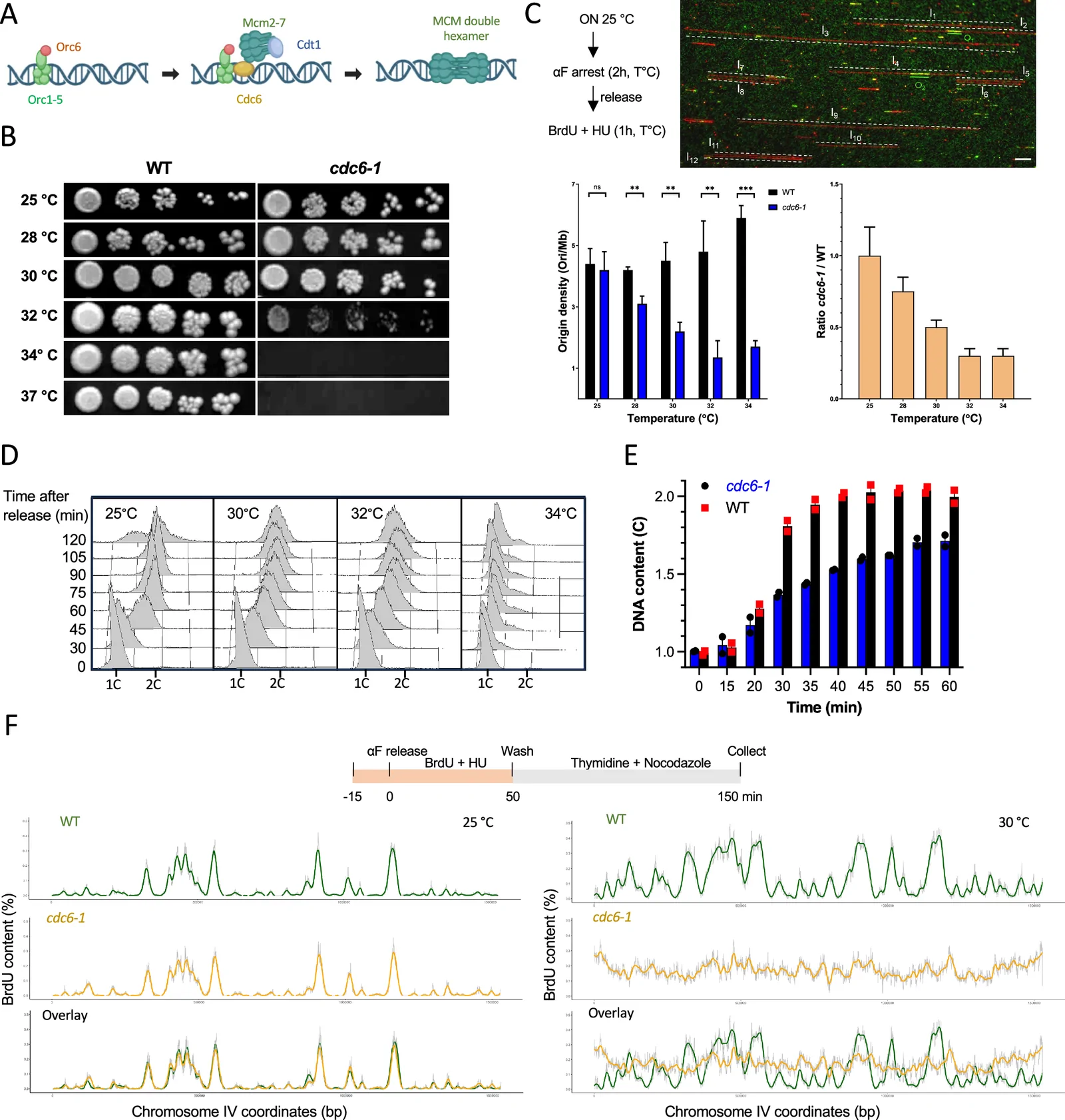
Origin under-licensing extends S phase and erodes chromosome replication profiles. (**A**) Cdc6 promotes origin licensing. (**B**) WT and *cdc6-1* cells spotted in fivefold serial dilutions on YPD plates were tested for viability at the indicated temperatures. (**C**) Replication origin density measured by DNA combing. WT and *cdc6-1* cells were synchronized in G_1_ with α-factor for 120–150 min at the indicated temperature, and released at the same temperature for 60 min in the presence of 50 µM BrdU to label nascent DNA and 200 mM HU to limit elongation. Representative image of DNA fibers stretched by DNA combing (red, dashed lines represent the length of each DNA fiber) with BrdU tracts (green; scale bar 10 µm). (Bottom) Density of active origins in WT and *cdc6-1* cells at different temperatures (mean ± SEM, *n* = 6, statistical analysis by Student *t*-test, at 25 °C, n.s. *p* value = 0.4458, at 28 °C **p value = 0.0003956, at 30 °C **p value = 9.296e-05, at 32 °C **p value = 5.655e-05, at 34 °C *** *p* value = 2.018e-07). Relative origin density in *cdc6-1* cells compared to WT. (**D**) S-phase progression by FACS in *cdc6-1* cells after G_1_ release at different temperatures. (**E**) DNA content by FACS of WT and *cdc6-1* cells after release from G_1_ at 30 °C (mean ± SEM, *n* = 3). *cdc6-1* cells have replicated only 70% of their genome, on average, 60 min after release from G_1_. (**F**) Chromosome IV replication profile by BrdU-nanopore sequencing of WT (green) and *cdc6-1* (yellow) cells at 25 and 30 °C. Cells were synchronized in G_1_ at 25 or 30 °C for 120 to 150 min. 100 µM BrdU and 200 mM HU were added 15 min prior to release from G_1_ and left for 50 min after the release; BrdU was then chased for 100 min with 200 µM thymidine and cells arrested in G_2_/M with 15 µg/mL nocodazole. Source data are available online for this figure.

To investigate the consequences on cell cycle progression and genome stability of extending S phase without perturbing fork progression with drugs, we lowered the number of active origins in *S. cerevisiae* using temperature-sensitive (ts) forms of the essential replication licensing factors Cdc6 and Cdt1 (Fig. 1A). *cdc6-1* mutant cells progressively lose viability with increasing temperatures (Fig. 1B), while displaying high rates of chromosome loss/rearrangements (Bruschi et al, 1995; Hartwell and Smith, 1985; Palmer et al, 1990). To quantify the effect of Cdc6 inactivation on DNA replication we measured the density of active origins in *cdc6-1* cells synchronized in G_1_ and released into S phase at various temperatures, by counting the density of BrdU tracts on single DNA molecules stretched by DNA combing (Fig. 1C). Origin density was similar in WT and *cdc6-1* cells at 25 °C but decreased progressively between 28 °C and 32 °C in *cdc6-1* cells (Fig. 1C). For further experiments we settled for the semi-permissive temperature of 30 °C, because viability was still high (90%; Fig. 1B) despite a twofold reduction in origin density and clear slowdown in S-phase progression (Fig. 1D,E).

**Figure EV1:**
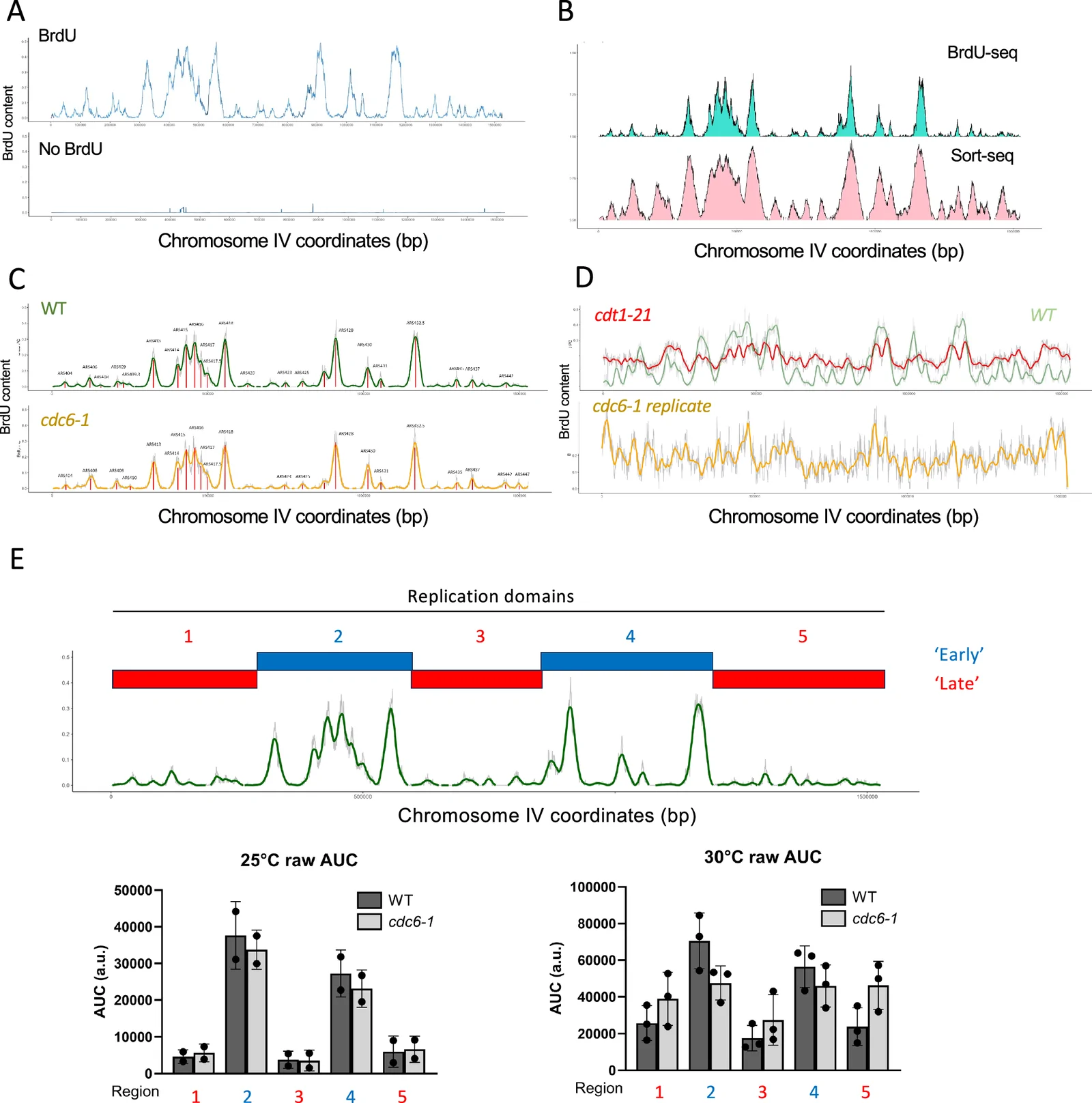
Validation of nanopore BrdU-seq pipeline (related to Fig. 1). (**A**) ChrIV replication profiles of WT cells grown in the presence (top) or absence (bottom) of BrdU, using our nanopore BrdU-seq detection pipeline. (**B**) Comparison of ChrIV replication profiles of WT cells obtained either by nanopore BrdU-seq or by copy-number-based Sort-seq method (Müller et al, 2014). Note that late-firing origins are detected in the BrdU-seq method despite using HU, yet at a lower level than in asynchronous cells not exposed to HU, as expected. (**C**) Replication origins identified on ChrIV in WT (green) or *cdc6-1* (orange) cells grown at 25 °C. Origins were called based on the position of BrdU profile peaks (local maxima) and the position of known ARS (OriDB Database). (**D**) ChrIV replication profiles of WT (green) and *cdt1-21* cells (red) grown at 30 °C (top), and a biological replicate of *cdc6-1* cells grown at 30 °C. (**E**) Quantification of BrdU incorporation within early and late replication domains on ChrIV. Profile (top) is WT at 25 °C. Area under the curve (AUC, bottom, mean ± SD) for the shown early and late domains of WT and *cdc6-1* cells grown at 25 °C (left, *n* = 2) or 30 °C (right, *n* = 3).

To test if *cdc6-1* cells at 30 °C fail to license only a subset of replication origins, we developed a method adapted from (Müller et al, 2019) for direct BrdU detection on long nanopore sequencing reads, which allows us to determine origin firing efficiencies genome-wide. WT and *cdc6-1* cells pre-grown at 25 °C were synchronized with α-factor at 25 or 30 °C and released for 60 min in rich medium containing BrdU to label active origins, and hydroxyurea (HU) to limit incorporation to 5–10 kb around them (Lengronne et al, 2001). BrdU-containing k-mers were called using DNAscent v3 (Jones et al, 2025) and summed over 500-bp windows (see Methods). WT cells synchronized at 25 or 30 °C and *cdc6-1* cells at 25 °C generated replication profiles that were dependent on the presence of BrdU (Fig. EV1A) and highly similar to those obtained by Sort-seq (Fig. EV1B), an orthogonal method based on copy-number variation during S phase and independent of cell synchronization (Müller et al, 2014), thus validating our pipeline (see full genome in Appendix Fig. S1). Replication profiles in WT and *cdc6-1* cells were almost identical at 25 °C, with early/efficient and late/inefficient origins producing high and low BrdU peaks overlapping known ARSs, respectively (Figs. 1F left and EV1C). This confirms the proficiency of *cdc6-1* cells at 25 °C and the reproducibility of our method. Strikingly, this replication landscape was highly eroded in the first S phase after shifting *cdc6-1* cells to 30 °C (Figs. 1F right and EV1D), indicating a stark decrease in the firing of early/efficient origins consistent with the overall 50% reduction in origin density measured by combing (Fig. 1C). Unexpectedly, the late/inefficient origins fired more frequently in HU-arrested *cdc6-1* cells at 30 °C, possibly because the intra-S checkpoint is less active (Mocanu et al, 2022; Shimada et al, 2002), because limiting firing factors are now available for less-efficient origins (Mantiero et al, 2011), or simply because inefficient origins now have more time to fire because they are not inactivated by incoming forks (Vujcic et al, 1999). The population-level loss of origin preference affected all chromosomes (Appendix Fig. S2) and was also evident in the *cdt1-21* origin-licensing mutant grown at semi-permissive temperature (Fig. EV1D). To quantify the effect, we measured the area under the curve for early and late-replicating domains, which shows that the former incorporate less BrdU, and the latter more, in early S of under-licensed cells (Fig. EV1E). These data indicate that reduced origin licensing in G_1_ not only extends S phase, but also alters the stereotypic replication profiles seen in populations of WT cells.

### *cdc6-1* cells enter mitosis with incompletely duplicated chromosomes

**Figure 2:**
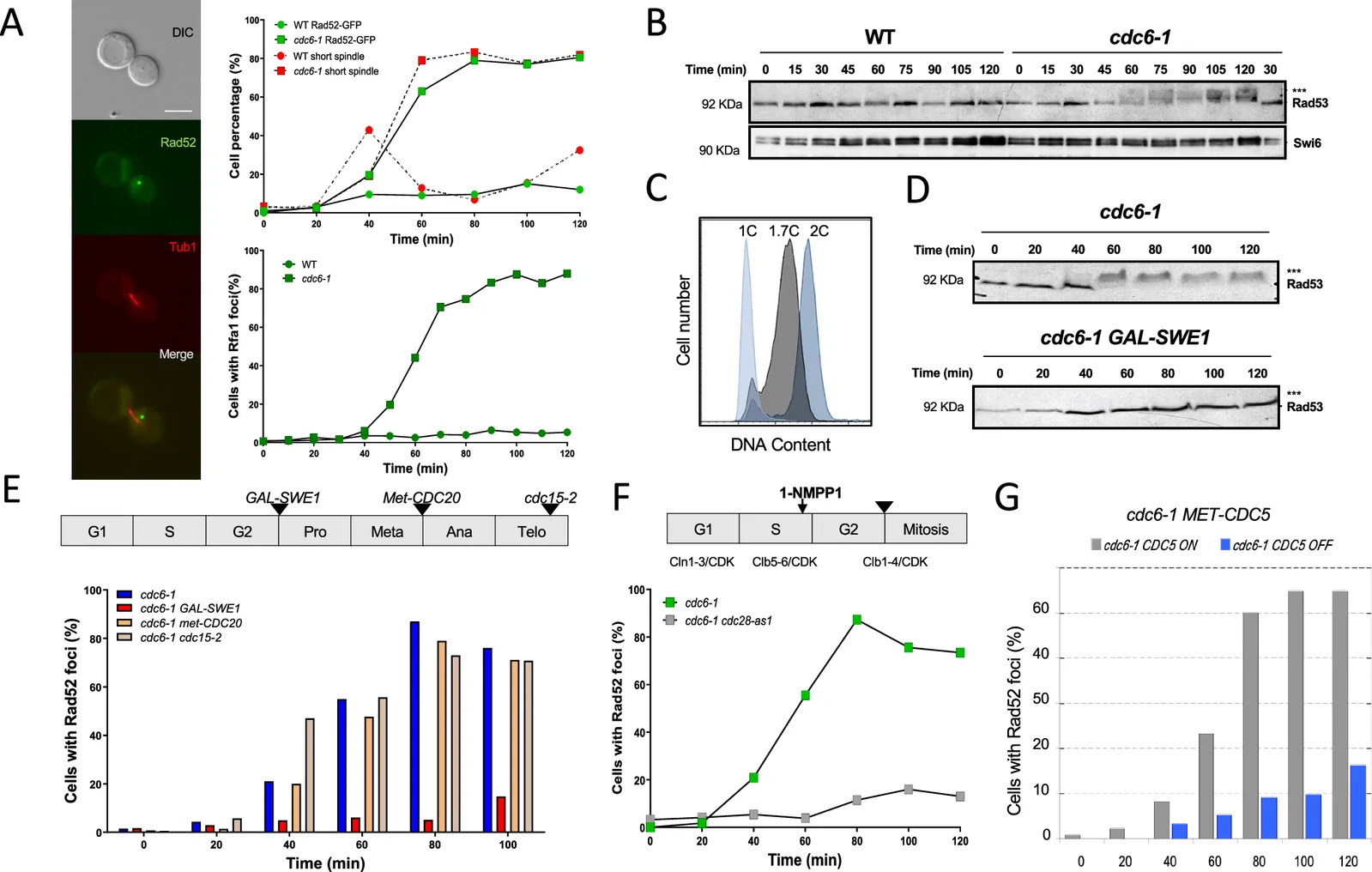
The DNA damage checkpoint is activated in *cdc6-1* cells, but only after mitotic entry. (**A**) Rfa1 and Rad52 form persistent foci in *cdc6-1* cells. WT and *cdc6-1* cells containing Rfa1-CFP or Rad52-GFP and Tub1-mCherry were released from G_1_ as before and analyzed by microscopy. Left, representative image of a *cdc6-1 RAD52-GFP TUB1-mCherry* cell 80 min after release (bar, 5 µm). Right, fraction of WT and *cdc6-1* cells containing Rad52 foci or metaphase spindles (top) and Rfa1 foci (bottom) at different times after G_1_ release. (**B**) Rad53 is hyperphosphorylated in *cdc6-1* cells. WT and *cdc6-1* cells were synchronized with α-factor at 30 °C, and Rad53 hyperphosphorylation (***) was monitored by western blot at different times after G_1_ release (Swi6, loading control). (**C**) FACS overlay comparing DNA content of WT (blue) and *cdc6-1* (gray) cells grown at 30 °C, 60 min after release. (**D**–**F**) DDR activation in *cdc6-1* cells depends on mitotic entry, not anaphase or cytokinesis. (**D**) Late G_2_ arrest by Swe1 overexpression prevents Rad53 hyperphosphorylation in *cdc6-1* cells. (**E**) Rad52 foci formation is prevented in *cdc6-1* cells arrested in late G_2_ (*GAL-SWE1*, YPGal 30 °C), but not in metaphase *(MET-CDC20*, +Met 30 °C) or in telophase (*cdc15-2*, YPD 37 °C). (F) *cdc6-1* and *cdc6-1 cdc28-as1* cells were synchronized with α-factor at 30 °C, released in YPD at 30 °C and 1-NMPP1 (2 µM) was added 30 min after release to prevent M-CDK activation. Rad52-GFP foci were scored by microscopy. (**G**) Percent cells with Rad52 foci in *cdc6-1* cells released from G_1_ arrest and expressing (gray) or not Cdc5 (blue). Source data are available online for this figure.

Next, we asked whether the extended S phase of *cdc6-1* cells caused replication stress and activated checkpoint responses. Indeed, the majority of *cdc6-1* cells grown at 30 °C accumulated Rfa1/RPA and Rad52 nuclear foci (Fig. 2A), indicative of ssDNA accumulation and DNA repair/recombination activity, respectively (Lisby et al, 2004). These cells also hyper-phosphorylated Rad53 that mediates the cellular DNA damage response (Fig. 2B). Crucially, these replication stress markers appeared only at 60 min after G_1_ release (Fig. 2A,B), not at 20 or 40 min when *cdc6-1* cells are fully engaged in DNA synthesis (Fig. 1D). We conclude that a slow S phase initiated from fewer origins does not activate a strong checkpoint response per se, but that another event occurring around 60 min after G_1_ release induces the DDR, at a time when *cdc6-1* cells grown at 30 °C have replicated ~70% of their DNA (Fig. 2C).

Yeast cells grown at 30 °C turn on mitotic cyclin-dependent kinases (M-CDK) 45 min (Clb3,4-Cdc28) and 60 min (Clb1,2-Cdc28) after G_1_ release, to promote spindle formation and elongation (Amon et al, 1993; Fitch et al, 1992; Surana et al, 1993). The conversion from short to long spindles did not take place in *cdc6-1* cells, which accumulated metaphase-sized spindles reminiscent of DNA damage-induced separase inhibition (Cohen-Fix and Koshland, 1997) (Fig. 2A). To test whether M-CDK activation or the tension exerted by spindles on partly-replicated chromosomes was responsible for DDR activation in *cdc6-1* cells, we prevented these cells either (i) to enter mitosis by overexpressing Swe1 (the ortholog of WEE1 kinase inhibiting M-CDK, not S-CDK; (Hu and Aparicio, 2005)), (ii) to undergo anaphase by depleting Cdc20 (regulator of the anaphase-promoting complex), or iii) to divide by inhibiting Cdc15 (the master kinase of the mitotic exit network). The results clearly showed that only the late G_2_ arrest induced by Swe1 overexpression suppressed Rad52 foci formation and Rad53 hyperphosphorylation in *cdc6-1* cells (Fig. 2D,E). Therefore, it is mitotic entry, not bipolar spindle formation or chromosome segregation, which is responsible for the strong DDR activation seen in *cdc6-1* cells at 30 °C.

**Figure EV2:**
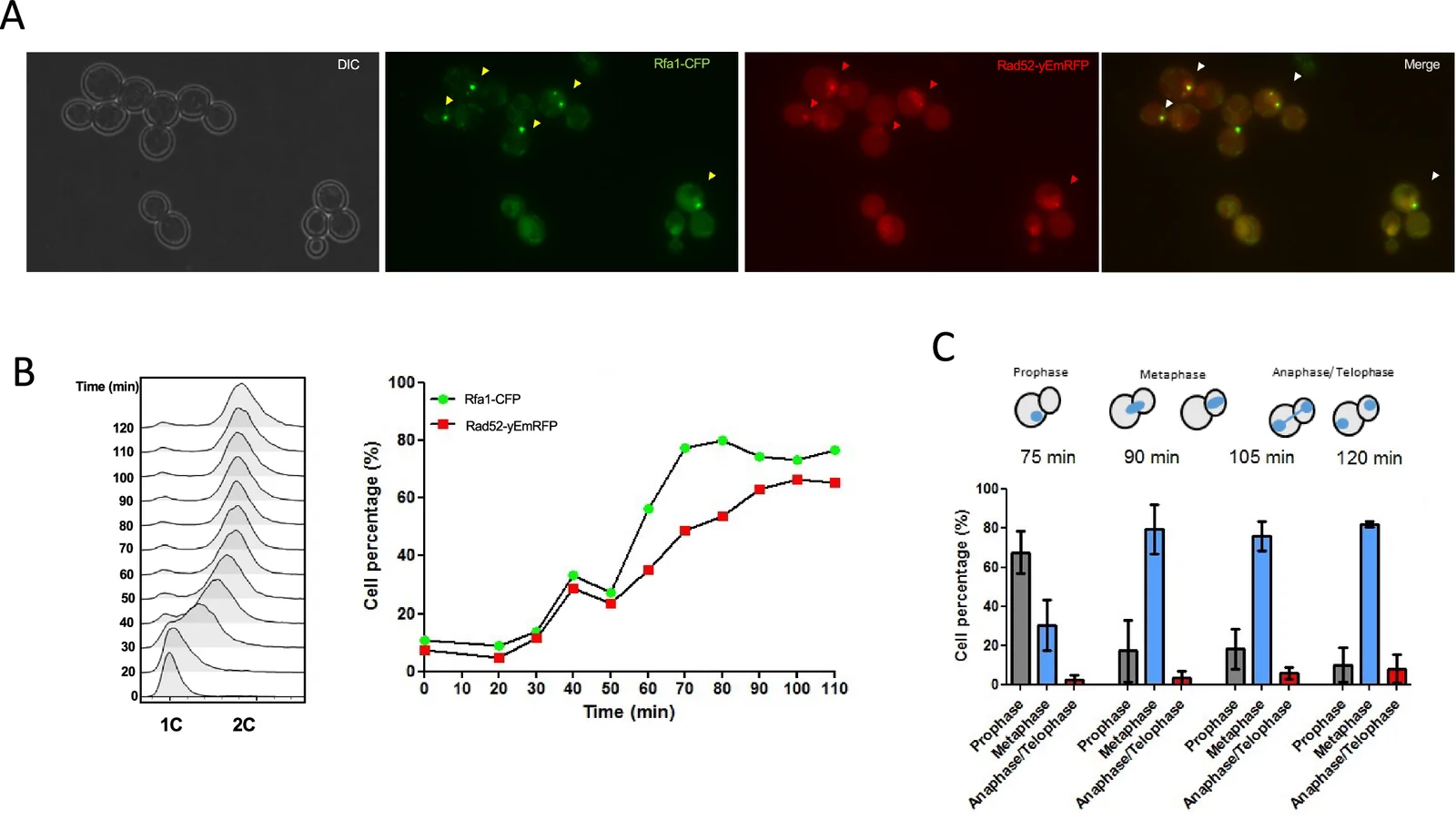
Spatial and temporal correlation of Rfa1 and Rad52 foci formation in *cdc6-1* cells (related to Fig. 2). (**A**) Representative images of asynchronous *cdc6-1 RFA1-CFP RAD52-yEmRFP* cells grown at 30 °C for 2 h (DIC, differential interference contrast; scale bar, 5 μm; arrowheads indicate foci). (**B**) Kinetics of Rfa1 and Rad52 foci formation in *cdc6-1* cells synchronized and released from G_1_ at 30 °C. DNA content by flow cytometry (left), fraction of cells containing Rfa1 or Rad52 foci (right; *n* = 2, >100 cells counted). (**C**) Accumulation of *cdc6-1* cells in metaphase. (Top) Schematic representing nucleus position relative to bud neck and daughter cell to classify cells in prophase, metaphase, ana/telophase. (Bottom) Fraction of cells in each class at the indicated time after G_1_ release *n* = 3, mean ± SD, >100 cells).

To test directly whether M-CDK was responsible for DDR activation in *cdc6-1* cells, we inactivated Cdk1 after S-phase entry (30 min) specifically with 1-NMPP1 using the *cdc28-as1* allele (Bishop et al, 2000). This suppressed Rad52 foci formation as well (Fig. 2F), demonstrating that M-CDK activity is required for DDR activation in *cdc6-1* cells. This assumption was confirmed by depleting Clb2 in *cdc6-1 clb1∆,3∆,4∆* cells. Depleting the Cdc5 polo-like kinase also impaired Rad52 foci formation in *cdc6-1* cells (Fig. 2G). We conclude that cells replicating from fewer active origins do not activate the DNA replication checkpoint strongly enough to prevent mitotic entry and thus enter mitosis with under-replicated DNA. M-CDK and Cdc5 activities then lead to DDR activation, possibly by causing fork collapse (Deng et al, 2019). Fixed and live-cell imaging indicated that Rfa1 and Rad52 foci appear simultaneously in *cdc6-1* cells, shortly before short spindles form, and persist for 20–60 min in metaphase-arrested cells displaying rapid spindle oscillations across the bud neck (Fig. EV2A,B; Movie EV1). Rad52 focus dissolution always preceded spindle elongation and mitotic exit in *cdc6-1* cells grown at 30 °C, which divided at a larger size and recapitulated the same scenario in subsequent cell cycles. In contrast, *cdc6-1* cells grown at the restrictive temperature of 34 °C, in which only 30% of early origins are active, failed to disassemble Rad52 foci, did not undergo anaphase, and eventually died at a very large size (Movie EV2). Thus, the survival of haploid yeast cells suffering from origin under-licensing is only possible when a substantial fraction of the genome has been duplicated before mitotic entry.

### Under-licensing causes MiDAS and chromosome rearrangements via Pol32-dependent mechanisms

**Figure 3:**
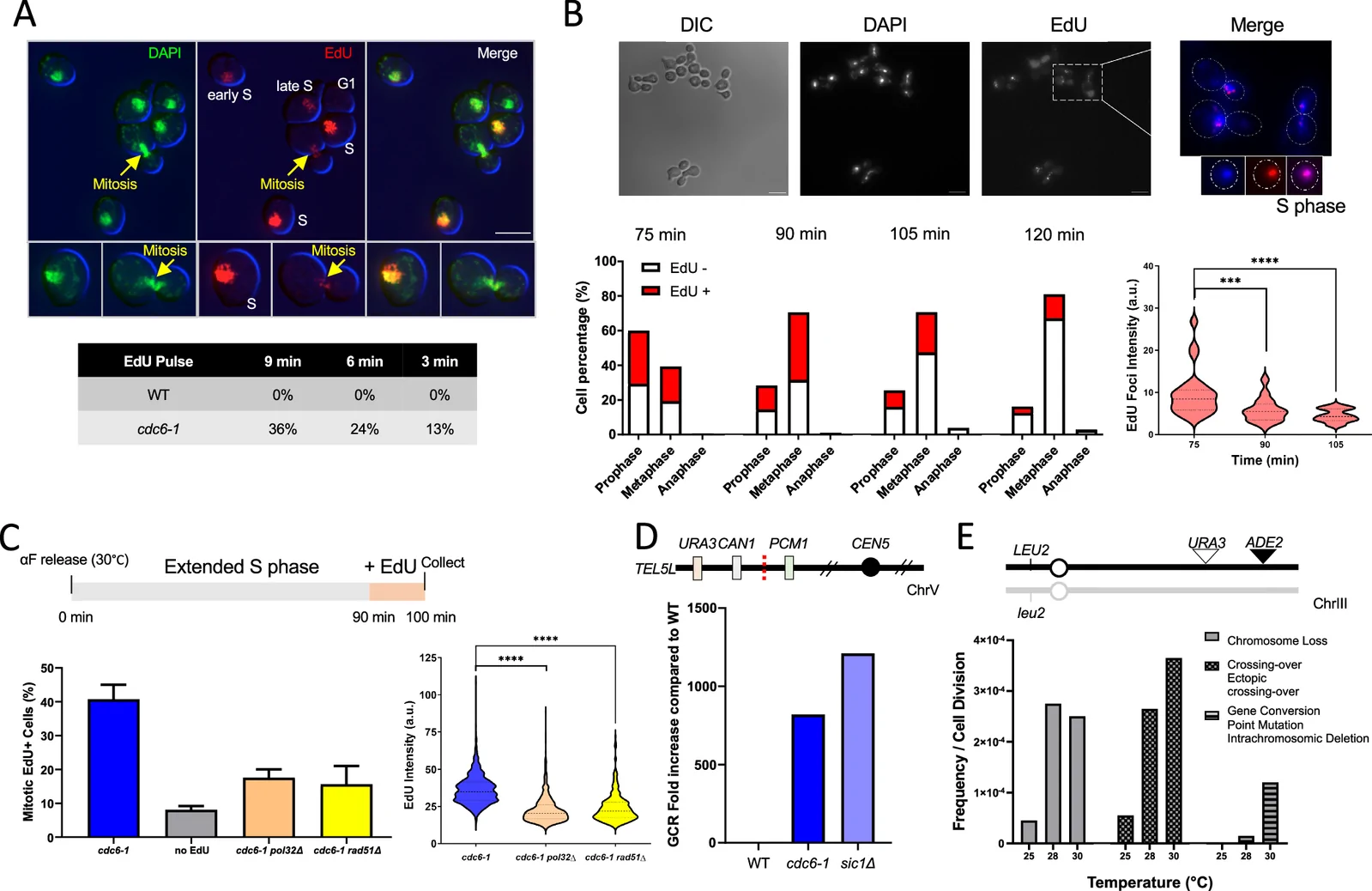
Cells with extended S phase undergo MiDAS and chromosome rearrangements. (**A**) Mitotic DNA synthesis in asynchronous *cdc6-1* cells grown at 30 °C for 1 h, pulsed with 10 µM EdU for 6 min and stained for DNA (DAPI, green) and EdU (Cy5-azide, red), along with cell contour (DIC, blue). Cells with stretched DAPI staining at the bud neck are considered in metaphase. Early, mid, and late S are classified according to bud size and pan-nuclear EdU staining (scale bar, 10 μm). Bottom, percentage of metaphase cells that are EdU positive (MiDAS) after pulses of 3, 6, or 9 min. (**B**) MiDAS in synchronized *cdc6-1* cells. Representative image of EdU signals in *cdc6-1* cells at 90 min after release from G_1_ (scale bar, 10 µm). Dashed circular borders indicate cell boundaries. Fraction of EdU^+^ and EdU^-^ *cdc6-1* cells at the indicated time after G_1_ release (10 µM EdU, 3 min, *n* = 2). Cells are categorized as prophase, metaphase, and anaphase based on the criteria described in Fig. EV2C. EdU signal intensity in *cdc6-1* cells at the indicated time after G_1_ release (median, first-quartile, and third-quartile represented with dashed lines, **** 75 min vs 90 min *p* value = 0.0004485, *** 75 min vs 105 min *p* value = 5.918e-07, statistical analysis by Mann–Whitney test, at least 25 cells were counted). (**C**) Quantitative Image-based Cytometry (QIBC) of MiDAS in cells synchronized with α-factor at 30 °C exposed for 10 min to 50 µM EdU or vehicle (no EdU) 90 min after G_1_ release. (Left) Percentage (Mean ± s.e.m.) of MiDAS-positive cells in *cdc6-1 rad51∆* and *cdc6-1 pol32∆* relative to *cdc6-1* (Right) Quantification of EdU foci intensity colocalizing with DAPI in the same strains (median, first-quartile and third-quartile represented with dashed lines; *cdc6-1* vs *cdc6-1 pol32∆* **** *p* values <2.2e-16, *cdc6-1* vs *cdc6-1 rad51∆* ****p value <2.2e-16; Statistical analysis by Mann–Whitney test). At least 2000 cells were analyzed in two biological replicates for each strain. (**D**) GCR rates (based on the loss of *URA3* and *CAN1* markers placed in a non-essential region near TEL5L) in haploid WT, *cdc6-1* and *sic1∆* cells grown at 30 °C. (**E**) Loss of heterozygosity (LOH) assay in diploid *cdc6-1* cells carrying heteroalleles of *LEU2*, *URA3*, and *ADE2* genes on ChrIII homologs. Rates of chromosome loss (5-FoA^r^ Leu^-^ Ade^-^), translocations and crossing over (5-FoA^r^ Leu^+^ Ade^-^), and intra-chromosomal deletion or gene conversion (5-FoA^r^ Leu^+^ Ade^+^) events were calculated after growing *cdc6-1* cells for 24 h at the indicated temperatures. Source data are available online for this figure.

Since *cdc6-1* cells grown at 30 °C enter mitosis with only 70% of their genome duplicated, yet survive, we wondered if DNA synthesis could be completed in mitosis, akin to MiDAS for late/difficult to replicate common fragile sites (CFS) in mammalian cells (Bhowmick et al, 2023; Minocherhomji et al, 2015). We first analyzed asynchronous WT and *cdc6-1* cells grown for 1 h at 30 °C to limit origin licensing in the latter, and scored by microscopy cells having their DNA stretching the bud neck (early mitosis) and EdU incorporation after a brief pulse. While strong EdU signals were present in S-phase cells in both WT and *cdc6-1* cells as expected, weaker EdU staining colocalizing with DNA in the bud neck was detected only in *cdc6-1* cells (Fig. 3A). The fraction of mitotic *cdc6-1* cells showing EdU signal varied between 13 and 36% according to the pulse duration (3 to 9 min), consistent with MiDAS proceeding asynchronously in different cells. The intensity of MiDAS signals in *cdc6-1* cells was roughly similar to cells labeled with EdU at the very beginning or very end of S phase (Fig. 3A, middle), but its localization was clearly distinguishable (foci at bud neck in cells with a large bud for MiDAS; pan-nuclear within mother cell for early S (no bud) or late S (medium-sized bud). Hence, MiDAS signals in early mitotic *cdc6-1* cells had an intensity commensurate with the amount of DNA still to replicate (30%), much stronger than expected for DNA repair. No EdU signal was detected in G_1_ and anaphase-telophase cells, neither WT nor *cdc6-1*. We conclude that MiDAS is prominent in under-licensed *cdc6-1* cells, but undetectable in WT yeast cells using our imaging conditions.

We repeated the experiment in synchronized *cdc6-1* cells pulsed with EdU for 3 min at 75, 90, 105, or 120 min after G_1_ release, to limit EdU signals stemming from late S-phase cells and to further explore the mitotic stage at which MiDAS takes place. The fraction of mitotic cells displaying EdU foci was highest (60%) at 90 min after release, mainly in cells with DNA close to the bud neck (prophase-metaphase), decreased thereafter (105, 120 min), and null in anaphase (Fig. 3B). The latter is concordant with Rad52 foci disappearing from *cdc6-1* cells just before anaphase (Movie EV1). Mean nuclear EdU intensity also decreased between 75 and 105 min. We conclude that MiDAS in *cdc6-1* cells starts in prophase, peaks in metaphase and declines during the prolonged metaphase arrest, possibly reflecting the gradual completion of genome replication.

**Figure EV3:**
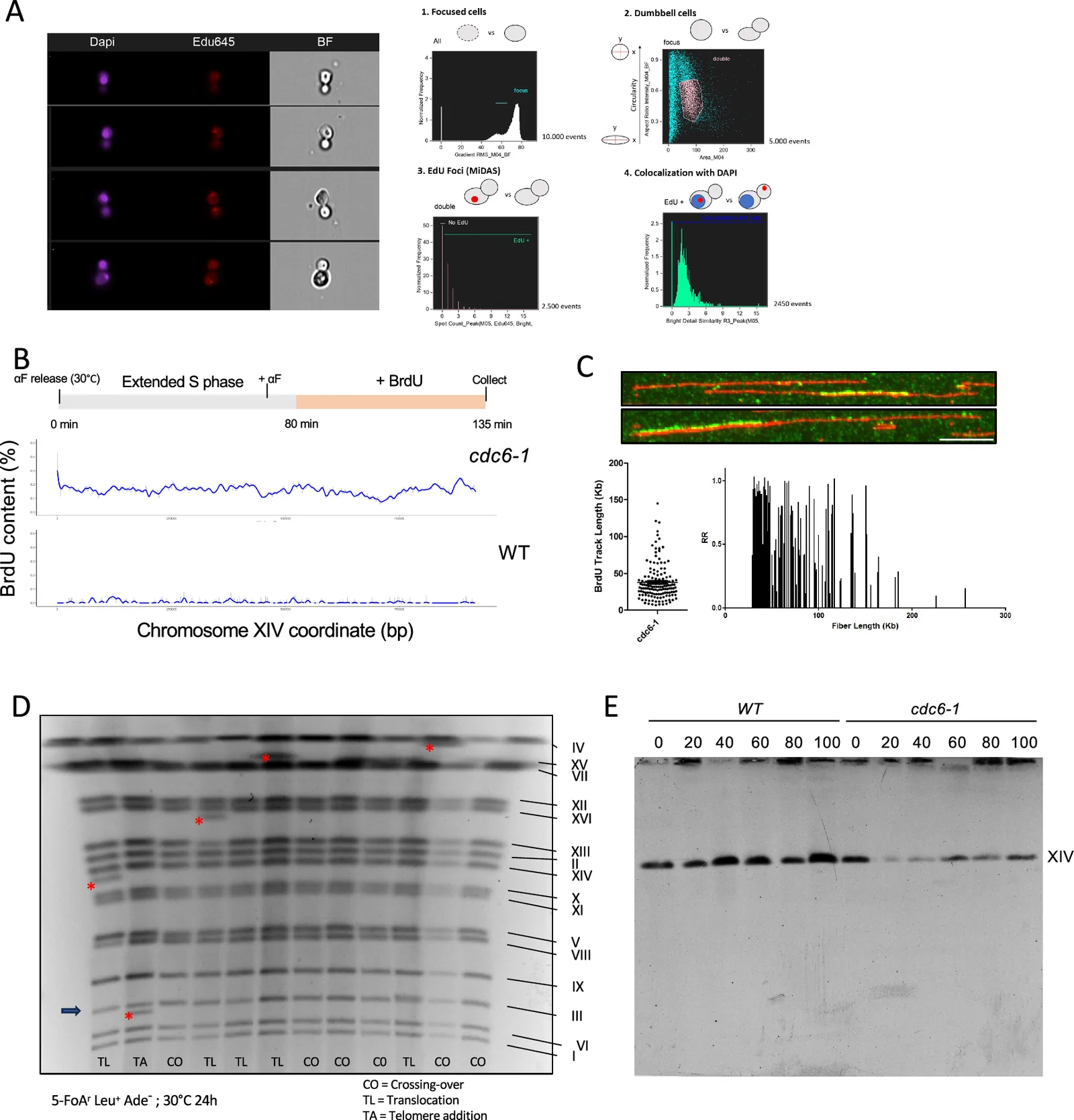
Mitotic DNA synthesis and chromosome instability in *cdc6-1* cells (related to Fig. 3). (**A**) QIBC for MiDAS detection in yeast. Representative images of MiDAS-negative, MiDAS-positive cell with single or multiple foci, false-positive cell with extranuclear EdU (left, top to bottom), and QIBC analysis workflow to identify MiDAS cells (right). (**B**) Pan-chromosome XIV mitotic DNA synthesis in *cdc6-1* cells pulsed with BrdU during mitosis (from 80 to 135 min after G_1_ release; α-factor re-added at 60 min), relative to WT control. (**C**) Detection of MiDAS BrdU tracts (green) on single DNA molecules (red) stretched by DNA combing (top; bar 10 µM). BrdU tract length for fibers ≥30 kb (left) and BrdU replacement rate (RR) on these fibers. BrdU-labeled DNA fibers are almost fully substituted up to 150 kb. (**D**) PFGE karyotype analysis of spontaneous 5-FoAr Leu+ Ade- clones isolated from diploid *cdc6-1* cells grown at 30 °C for 24 h. Translocations (TL) are told apart from equal crossing-overs (CO) by the decrease in ChrIII band intensity (arrow) and the appearance of a new chromosome band (asterisk). See Hiraoka et al (2000) for details. (**E**) Lack of detectable DSB in *cdc6-1* cells released from G_1_ arrest at 30 °C and undergoing MiDAS. PFGE-Southern blot analysis with *POL1* radioactive probe labeling ChrXIV.

**Figure EV4:**
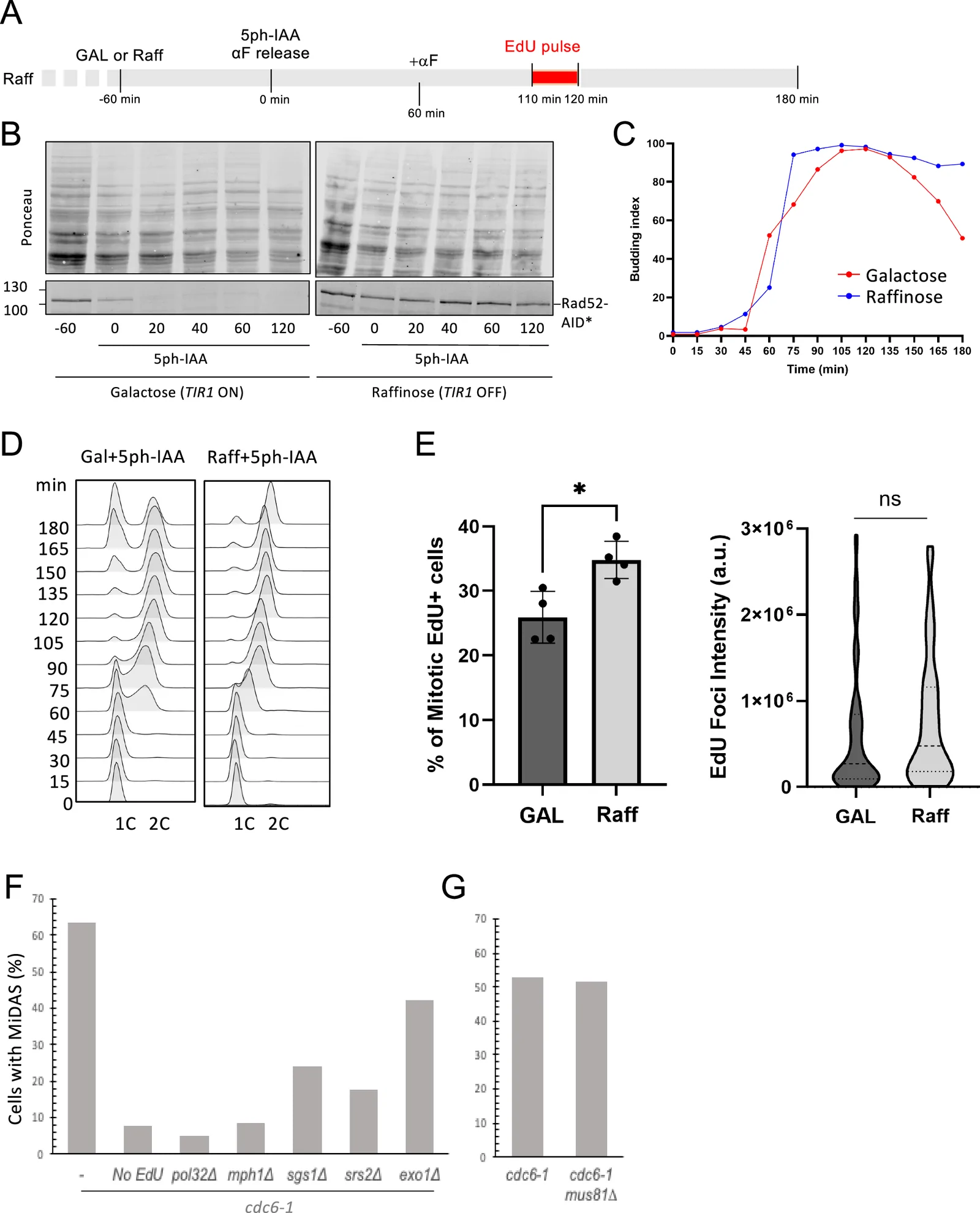
MiDAS in *cdc6-1* cells is partly dependent on Rad52 and independent of *MUS81* (related to Fig. 3). (**A**) *cdc6-1, RAD52-AID2*, and* GAL-OsTIR1* cells were depleted of Rad52, or not, by auxin addition in the presence (Gal) or absence (Raff) of OsTIR1 before release from G_1_ arrest. Cells were pulsed for 10 min with EdU at 110 min to detect MiDAS, and α-Factor was added back to prevent cells from entering a subsequent S phase. (**B**) Western blot analysis of Rad52-AID depletion. (**C**,** D**) Budding index and FACS data showing that *cdc6-1* cells exit mitosis precociously in the absence of Rad52 (Gal). (**E**) Percent cells undergoing MiDAS (left, *n* = 4 mean ± SD, >100 cells in each replicate, *p value = 0.02857, statistical analysis by Mann–Whitney test) in the absence (Gal) or presence (Raff) of Rad52, along with EdU foci intensities (right, at least 160 cells were counted, mean ± SD, n.s. *p* value = 0.9986, statistical analysis by Mann–Whitney test). (**F**) QIBC scoring of MiDAS in *cdc6-1* cells deleted for the indicated genes. (**G**) MiDAS scored by fluorescence microscopy in *cdc6-1* cells lacking or not *MUS81*.

To explore which factors are required for MiDAS in under-licensed cells, we set up quantitative image-based cytometry in yeast (QIBC; Fig. EV3A) to score at high-throughput the fraction of cells displaying MiDAS and the intensity of EdU foci in *cdc6-1* cells deleted for different recombination/repair genes. Rad51 and Pol32 (an accessory subunit of Pol ∂) are important for homologous recombination (HR) and break-induced replication (BIR) or microhomology/microsatellite-induced replication (MMIR), respectively (Lydeard et al, 2007; Ogawa et al, 1993; Payen et al, 2008). Deleting either of these genes in *cdc6-1* cells led to a strong reduction of both the fraction of cells undergoing MiDAS and EdU foci intensity (Figs. 3C and EV4F). MiDAS was also reduced in *cdc6-1* cells lacking Mph1, Srs2, or Sgs1, but surprisingly only moderately in cells lacking Rad52 and not at all in those lacking Mus81 (Fig. EV4E,G). Since we failed to detect DSBs by pulse-field gel electrophoresis (PFGE; Fig. EV3E) in *cdc6-1* cells, we propose that MiDAS induced by origin under-licensing occurs via a combination of Pol32-dependent, Rad52-dependent and independent recombination-dependent replication mechanisms that may not require an initial DSB, such as BIR-related fork stalling and template switching (FoSTeS) or MMIR using microhomologies to restart a fork on an ectopic template (Epum and Haber, 2022; Lee et al, 2007; Payen et al, 2008).

Next, we asked if MiDAS takes place at specific genomic loci in yeast, as for common fragile sites (CFS) in humans or at sites of transcription-replication conflicts (TRC) in BRCA2^−/−^ cells (Groelly et al, 2022; Macheret et al, 2020; Minocherhomji et al, 2015). We used our nanopore BrdU detection pipeline, but this time by adding BrdU to *cdc6-1* cells during mitosis (from 80 to 135 min after G_1_ release), while adding back α-factor to prevent entry into the next S phase (Fig. EV3B). BrdU was detected at significant levels only in *cdc6-1*, not WT cells, and along the entire length of all chromosomes, with some shallow peaks and valleys (Appendix Fig. S3). We conclude that MiDAS in *cdc6-1* cells is not restricted to specific loci, as expected from the genome-wide reduction in origin firing, its stochastic nature, the duration of the BrdU pulse and the processivity of BIR mechanisms. Indeed, using DNA combing, we detected BrdU staining of long distance (mean 45 kb, max 150 kb) along single DNA molecules (Fig. EV3C), consistent with the processivity of BIR (Davis and Symington, 2004; Wu and Malkova, 2021).

Since recombination-dependent replication can invade heterologous chromosomes, we used two genetic assays to detect chromosome rearrangements in *cdc6-1* cells grown at 30 °C. The GCR assay determines the frequency at which haploid cells break and lose the distal part of ChrV-L, yet remain viable either by telomere addition or by invading and copying a heterologous chromosome until its telomere, generating a non-reciprocal translocation (Chen and Kolodner, 1999). *cdc6-1* cells grown at 30 °C displayed an 800-fold increased GCR rate relative to WT, comparable to *sic1∆* cells activating S-CDK precociously also suffering from incomplete origin licensing (Fig. 3D) or to *rad52∆* or *mre11∆* cells with inactive recombinational repair (Chen and Kolodner, 1999; Lengronne and Schwob, 2002). The second assay, which scores loss of heterozygosity (LOH) of three markers on ChrIII, assesses the rate of chromosome loss, crossing over and gene conversion in diploids. We scored recombination rates per cell division in *cdc6-1/cdc6-1* cells grown at 25, 28, or 30 °C for 24 h, and found that all three types of events were increased at 28° and 30 °C, with a 31-fold increase per cell division in gene conversion or deletion of the internal *URA3* marker (Fig. 3E). Complete ChrIII loss was increased fivefold and crossovers sixfold per cell division at 30 °C compared to 25 °C. For crossovers, pulse-field gel electrophoresis (PFGE) revealed single or multiple ectopic recombination events involving other chromosomes (Fig. EV3D) (Hiraoka et al, 2000). We conclude that chromosome instability is prominent in cells that cannot fully license their replication origins, due to a failure to complete chromosome duplication before mitotic entry. This then promotes recombination-dependent replication during prophase/metaphase using Pol32-dependent BIR-related mechanisms.

### Polα phosphorylation by M-CDK is essential for the viability of cells with an extended S phase

**Figure 4:**
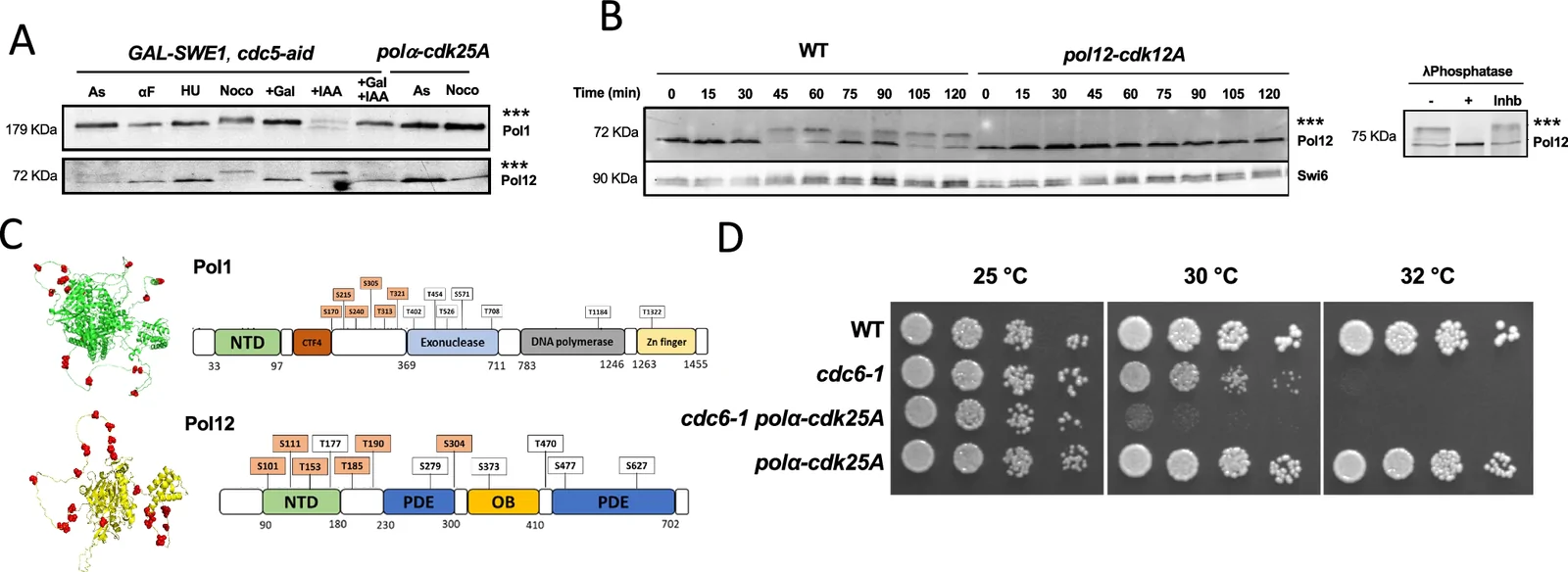
Cells with extended S phase require mitotic DNA Polα phosphorylation for survival. (**A**) DNA Polα is phosphorylated in mitosis in a CDK-dependent manner. Pol1 and Pol12 western blot mobility shift assay in asynchronous (As), α-factor (αF), hydroxyurea (HU), or nocodazole (Noco) arrested *GAL-SWE1*, *cdc5-aid* cells. Galactose (+Gal), auxin (+IAA) or both (+Gal, +IAA) were added to nocodazole-arrested cells to test for M-CDK and Cdc5-dependence of the mobility shift. *polα-cdk25A* is a strain in which all 13 and 12 putative Cdk phospho-acceptor sites in *POL1* and *POL12* were mutated to alanine, respectively (Appendix Figs. S4 and S5). (**B**) Pol12 mobility shift is due to phosphorylation. (Left) Pol12 phosphorylation kinetics in synchronous WT and *pola-cdk25A* cells after release from G_1_. (***, phospho-forms; Swi6, loading control). (Right) Pol12 immuno-precipitates from nocodazole-arrested cells were exposed to vehicle (−), λ Phosphatase (+) or the latter plus 10 mM sodium o-vanadate (Inhib) (***, phospho-forms). (**C**) Mitotic Pol1 and Pol12 phosphorylation sites identified by phospho-mass spectrometry. (Left) AlphaFold-predicted structures of Pol1 and Pol12 with identified phosphorylation sites (red balls). (Right) Schematic representations of Pol1 and Pol12, with corresponding domains (NTD N-terminal domain, PDE phosphodiesterase, OB oligonucleotide binding fold). Putative CDK sites (S/T-P) are indicated, with those confirmed by phospho-MS highlighted in orange. (**D**) Viability assay of WT, *cdc6-1, cdc6-1 polα-cdk25A*, and *polα-cdk25A* cells. Source data are available online for this figure.

M-CDK activity being required for Rad52/Rfa1 foci formation and Rad53 hyper-phosphorylation in under-licensed cells (Fig. 2D–F), we reasoned that DDR activation might stem from fork collapse and therefore searched for replisome components that are phosphorylated by CDK in G_2_ or mitosis (Holt et al, 2009). The two largest subunits of DNA Polα, Pol1/POLA1 catalytic- and Pol12/POLA2 B-subunit, are hyper-phosphorylated by CDK in yeast and human cells, causing Polα eviction from chromatin and its inability to initiate DNA synthesis on the SV40 genome, respectively (Desdouets et al, 1998; Foiani et al, 1995; Voitenleitner et al, 1999). We confirmed that both Pol1 and Pol12 undergo a mobility shift in mitosis (Noco), but not in G_1_ (αF) or S phase (HU). This shift is suppressed by Swe1 overexpression and is thus likely M-CDK dependent (Fig. 4A). Indeed, in synchronized WT cells, Pol12 undergoes a strong mobility shift at 45 min after G_1_ release, when Clb3,4-CDKs become active, and until 75 min as cells undergo anaphase and exit mitosis. The Pol12 mobility shift depends on phosphorylation and is lost when its 12 putative CDK sites are mutated to alanine (*pol12-cdk12A*; Fig. 4A,B). This demonstrates that Pol12, and likely Pol1, are phosphorylated by CDK at mitotic entry.

To identify the sites phosphorylated in vivo, we analyzed by immunoprecipitation and tandem mass spectrometry (IP-MS/MS) the phosphorylation status of both Pol1 and Pol12 during mitosis in *cdc6-1* cells, after an extended S phase. We identified multiple high-confidence phosphorylation sites in both Pol1 and Pol12 (Table EV1), which were clustered in the disordered N-ter domains of both proteins (Fig. 4C). Those include several of the putative CDK phospho-acceptor sites in Pol1 and Pol12 that we mutated to Ala in the *pol1-cdk13A, pol12-cdk12A* strain (hereafter named *polα-cdk25A;* Appendix Figs. S4 and S5). Other non-CDK sites were also found phosphorylated, some predicted to be targets of Cdc5/Plk1, consistent with our observation of reduced Rad52 foci formation upon Cdc5 depletion in *cdc6-1* cells (Fig. 2G). We conclude that Pol1 and Pol12 are phosphorylated at CDK and Cdc5 sites in vivo in mitosis after an extended S phase in *cdc6-1* cells, which makes DNA Polα a prime candidate for being a key target responsible for remodeling replication forks at mitotic entry, which leads to DDR activation, illustrated by Rfa1/Rad52 foci and Rad53 phosphorylation.

**Figure EV5:**
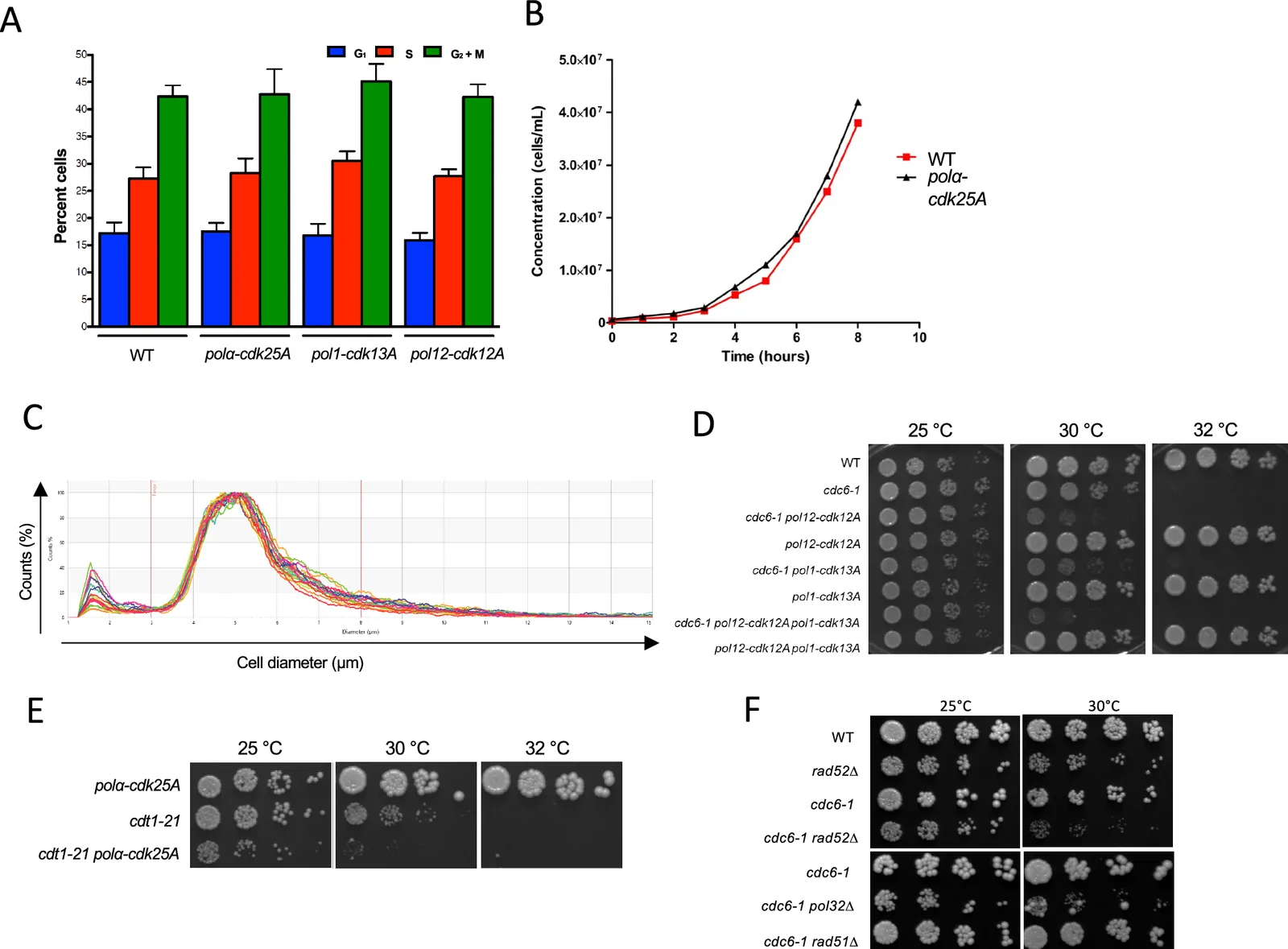
Polα-cdk25A is proficient for canonical DNA replication, but lethal in hypo-licensed cells (related to Fig. 4). (**A**) Cell cycle phases duration of WT, *pol1-cdk13A, pol12-cdk12A*, and *polα-cdk25A* mutants calculated from EdU-PI bivariate FACS and cell doubling time (mean ± SEM, *n* = 3 biological replicates). (**B**) Growth curve of WT and polα-cdk25A mutant. (**C**) Cell size distribution of WT and polα-cdk25A cells. (**D**) WT, *pol1-cdk13A, pol12-cdk12A*, and *polα-cdk25A* cells containing or not *cdc6-1* were spotted in fivefold serial dilutions on rich medium and grown at the indicated temperatures. (**E**) Synthetic lethality of *polα-cdk25A* and *cdt1-21* mutations. Cells were spotted in fivefold serial dilutions and grown for 2 days at the indicated temperatures. (**F**) *cdc6-1* shows co-lethality with *rad52∆* and *pol32∆*, but not with *rad51∆*.

A prediction of this model is that non-phosphorylable DNA Polα mutants should be defective in checkpoint activation after an extended S phase, and possibly lethal in combination with *cdc6-1* cells at semi-permissive temperature. In agreement with this hypothesis, we found that *cdc6-1 polα-cdk25A* double mutants lost viability at 30 °C, unlike the *cdc6-1* and *polα-cdk25A* single mutants (Fig. 4D). Importantly, the viability of the *polα-cdk25A* mutant at any temperature in cells with normal licensing indicates that DNA Polα phosphorylation is dispensable for canonical DNA replication. Indeed, the duration of S phase, the doubling time and cell size distribution of *pol1-cdk13A*, *pol12-cdk12A* and *polα-cdk25A* cells were indistinguishable from WT (Fig. EV5A–C), demonstrating that non-phosphorylable Polα-cdk25A is fully proficient for genome duplication in cells with normal origin licensing. Both *pol1-cdk13A* and *pol12-cdk12A* contributed to the lethality of *cdc6-1* cells at 30 °C, and their effect were additive (Fig. EV5D). Finally, *polα-cdk25A* was also synthetic lethal with the *cdt1-21* replication licensing mutant (Jacobson et al, 2001) (Fig. EV5E). This demonstrates that DNA Polα phosphorylation at CDK sites is dispensable for normal S phase, but is essential for cells that have an extended S phase due to origin under-licensing.

### Polα phosphorylation triggers the DDR and promotes MiDAS after an extended S phase

**Figure 5:**
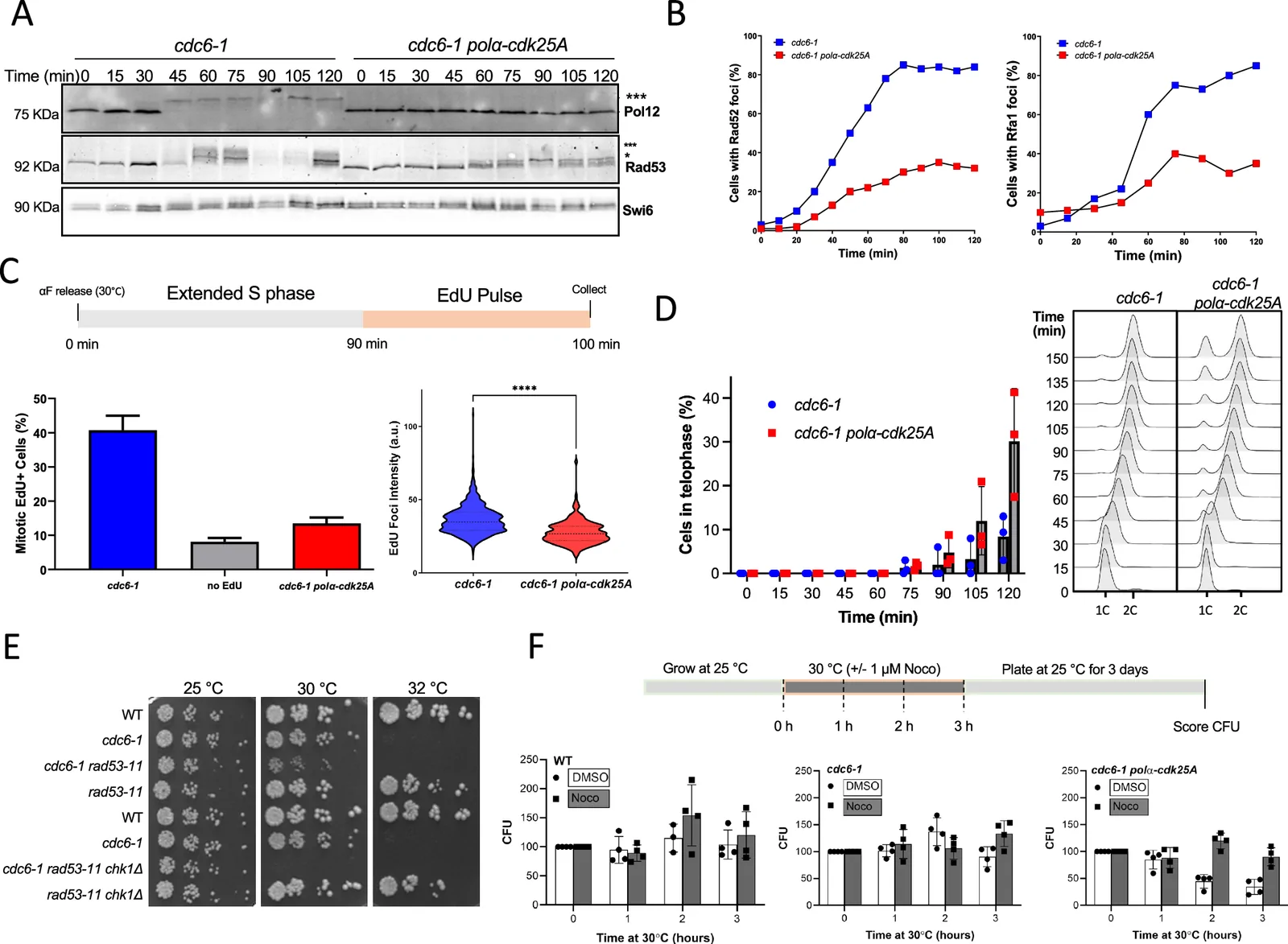
Polα phosphorylation is required for cell cycle arrest and proficient MiDAS in cells with extended S phase. (**A**) Rad53 and Pol12 phosphorylation in *cdc6-1* and *cdc6-1 polα-cdk25A* cells after α-factor release at 30 °C (hyper-phosphorylated Pol12 and Rad53 forms are marked by ***; Swi6, loading control). (**B**) Rad52-GFP and Rfa1-CFP foci formation in *cdc6-1* and *cdc6-1 polα-cdk25A* cells after α-factor release at 30 °C (*n* = 3). (**C**) QIBC analysis of MiDAS in *cdc6-1* and *cdc6-1 polα-cdk25A* cells exposed for 10 min with vehicle (DMSO; no EdU) or 50 µM EdU at 90 min after α-factor release at 30 °C. Fraction of MiDAS-positive in *cdc6-1 polα-cdk25A* relative to *cdc6-1* (mean ± s.e.m.) (left) and EdU foci intensity (right; *cdc6-1* vs *cdc6-1 polα-cdk25A* ****p value <2.2e-16, statistical analysis by Mann–Whitney test). At least 2000 cells were analyzed in two biological replicates. (**D**) Polα CDK mutants exit mitosis prematurely after extended S phase. *cdc6-1* and *cdc6-1 polα-cdk25A* cells were released from α-factor arrest in SC medium at 30 °C and telophase cells were scored by microscopy (two separated DNA masses) (left, *n* = 3), and DNA content analyzed by flow cytometry (right). (**E**) *cdc6-1* cells grown at semi-permissive temperature require Rad53 and Chk1 for viability. (**F**) Delaying anaphase rescues *cdc6-1 polα-cdk25A* lethality. Cells were shifted at 30 °C for the indicated times in presence of DMSO (vehicle) or a sub-cytostatic concentration of nocodazole (1 µM) to delay anaphase, then plated on YPD at 25 °C for 3 days to count colony-forming units (CFU, normalized at 100 for 0 h), (mean ±  s.e.m., *n* = 3). Source data are available online for this figure.

To test if DNA Polα phosphorylation is required for checkpoint activation when cells containing under-replicated DNA enter mitosis, we monitored Rad53 phosphorylation as well as Rad52 and Rfa1 focus formation in synchronized *cdc6-1* and *cdc6-1 polα-cdk25A* cells. In the former strain, Pol12 was phosphorylated 45 min after G_1_ release, shortly before Rad53 was phosphorylated. Neither Pol12 nor Rad53 proteins underwent the same mobility shift in the *cdc6-1 polα-cdk25A* strain (Fig. 5A; the residual Rad53 doublet is due to metaphase arrest, not checkpoint activation; see (Diani et al, 2009; Magiera et al, 2014). Rad52 or Rfa1 foci formation decreased strongly in *cdc6-1 polα-cdk25A* cells (Fig. 5B), as well as the intensity of these foci. Crucially, the fraction of *cdc6-1* cells displaying MiDAS EdU foci, and their intensity, decreased significantly in the presence of non-phosphorylable Polα (Fig. 5C). Together, these data demonstrate that DNA Polα phosphorylation by M-CDK is instrumental for a robust DDR activation in cells that enter mitosis with ongoing replication forks. We propose that this checkpoint activation and metaphase arrest is, in turn, essential for a productive MiDAS.

**Figure EV6:**
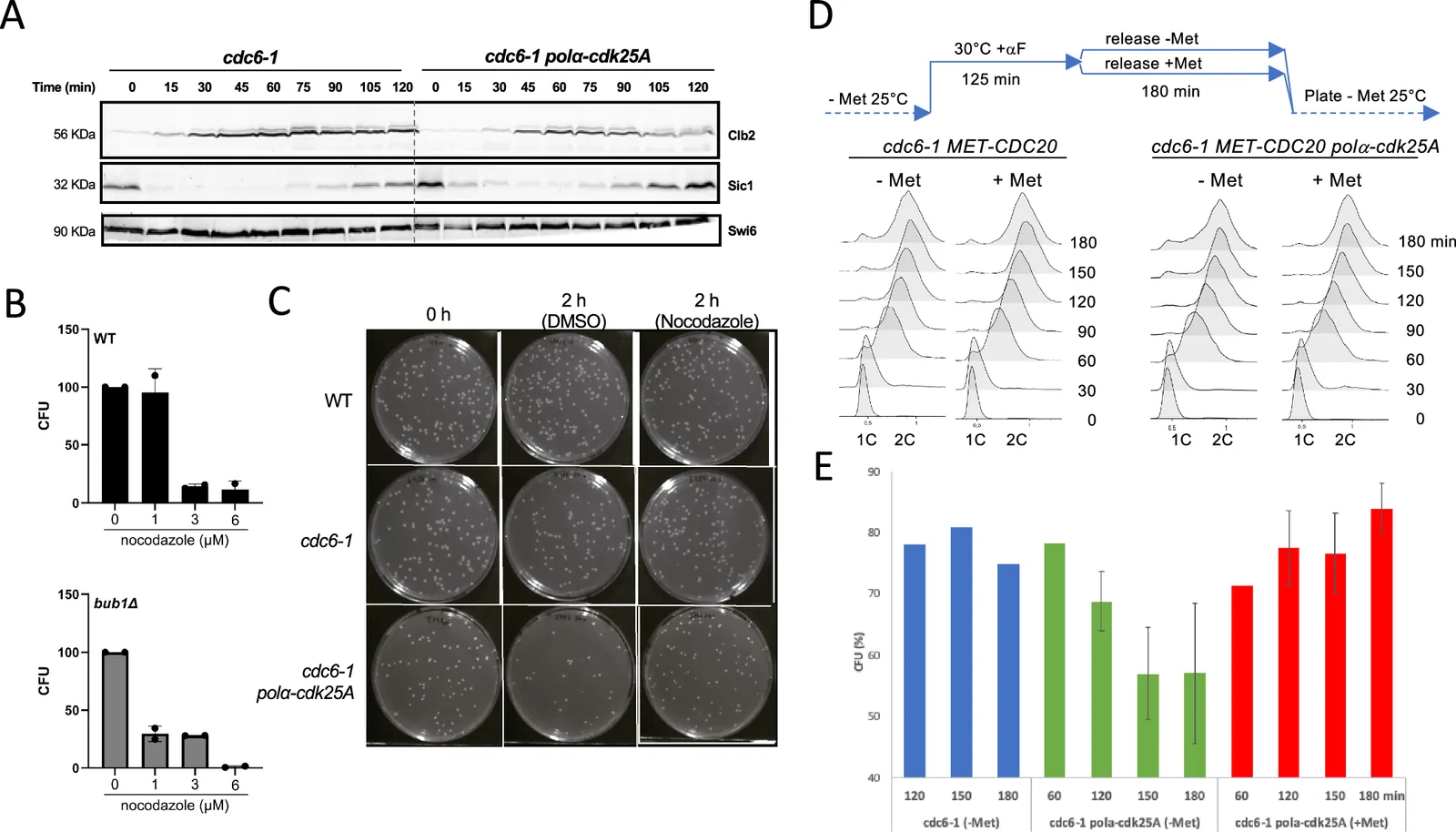
Polα phosphorylation is required for prolonged metaphase arrest in *cdc6-1* cells (related to Fig. 5). (**A**) Western blot analysis monitoring Sic1 and Clb2 levels in *cdc6-1* and *cdc6-1 polα-cdk25A* cells after release from G_1_ at 30 °C (Swi6, loading control). (**B**) Low dose nocodazole (1 µM) slows down mitosis without affecting viability of WT cells. Colony formation assay of WT and *bub1Δ* cells after 4 h incubation with nocodazole at the indicated concentrations. CFU values are normalized to timepoint 0 h set at CFU = 100 (mean ± SEM, *n* = 3). (**C**) Colony-forming assay for WT, *cdc6-1* and *cdc6-1 polα-cdk25A* cells grown at 30 °C for 0 or 2 h in presence of DMSO (vehicle) or nocodazole before plating. (**D**) *cdc6-1 MET-CDC20* and *cdc6-1 MET-CDC20 polα-cdk25A* cells were synchronized in G_1_ at 30 °C and released in the presence (-Met) or absence (+Met) of *CDC20* synthesis. Cell cycle progression by cytometry of DNA content. At different times after release, cells were plated on –Met 25 °C to score for viability. (**E**) Colony-forming units (CFU) for cells spotted at different times after G_1_ release +/−Met. The drop in viability seen after 120 min in the *cdc6-1 pola-cdk25A* cells (−Met) is suppressed by transient metaphase arrest induced by Cdc20 depletion (+Met). Mean ± SD for 120, 150, and 180 min timepoints (*n* = 3, technical triplicate; 2 biological duplicates).

The fact that the DDR (measured by Rad53 hyperphosphorylation, Rfa1 and Rad52 foci formation) fails to be activated properly in *cdc6-1 polα-cdk25A* cells is corroborated by their faster mitotic exit and re-entry into G_1_, compared to *cdc6-1* cells (Figs. 5D and EV6A). Unexpectedly, *cdc6-1* cells depleted of Rad52 also exited mitosis precociously and lost viability (Figs. EV4A–D and EV5F), indicating that Rad52 presence is necessary to maintain DDR activation, as proposed previously (Lee et al, 2003). This strengthens our hypothesis that the completion of DNA synthesis in mitosis requires a prolonged metaphase arrest. Consistently, we find that *cdc6-1* cells also rely on *RAD53* and *CHK1* for viability at 30 °C (Fig. 5E).

DNA Polα phosphorylation being required for checkpoint activation and a prolonged metaphase arrest does not rule out a direct role in promoting MiDAS. To test this possibility, we reasoned that if the sole function of DNA Polα phosphorylation was to activate the checkpoint (providing time in metaphase for MiDAS to take place), then granting more time in metaphase by another means should rescue the lethality of *cdc6-1 polα-cdk25A* cells. Hence, we exposed WT, *cdc6-1*, and *cdc6-1 polα-cdk25A* cells at 30 °C for 0 to 3 h to a sub-cytostatic concentration of nocodazole, which transiently activates the spindle-assembly checkpoint, causing a metaphase delay with no incidence on cell viability (Wang and Burke, 1995) (Fig. EV6B). Cells were then plated at 25 °C without nocodazole and scored for viability. While the metaphase delay induced by low-dose nocodazole had no effect on the viability of WT and *cdc6-1* cells, it did suppress the viability loss of the *cdc6-1 polα-cdk25A* double mutant at 30 °C (Figs. 5F and EV6C). This result was confirmed by imposing a metaphase delay via Cdc20 depletion instead of nocodazole, to provide *cdc6-1 polα-cdk25A* cells with extra time to complete DNA synthesis without activating the spindle-assembly checkpoint (Fig. EV6D,E). This suggests that the primary role of Polα phosphorylation is to trigger the DDR when cells enter mitosis with under-replicated chromosomes, and that MiDAS can occur, yet at a slower pace and perhaps using redundant mechanisms, independently of Polα phosphorylation. This does not rule out a role of Polα for second-strand BIR synthesis, as shown by others (Donnianni et al, 2019).

### Phospho-Polα is evicted from chromatin at mitosis, remodeling the replisome

**Figure 6:**
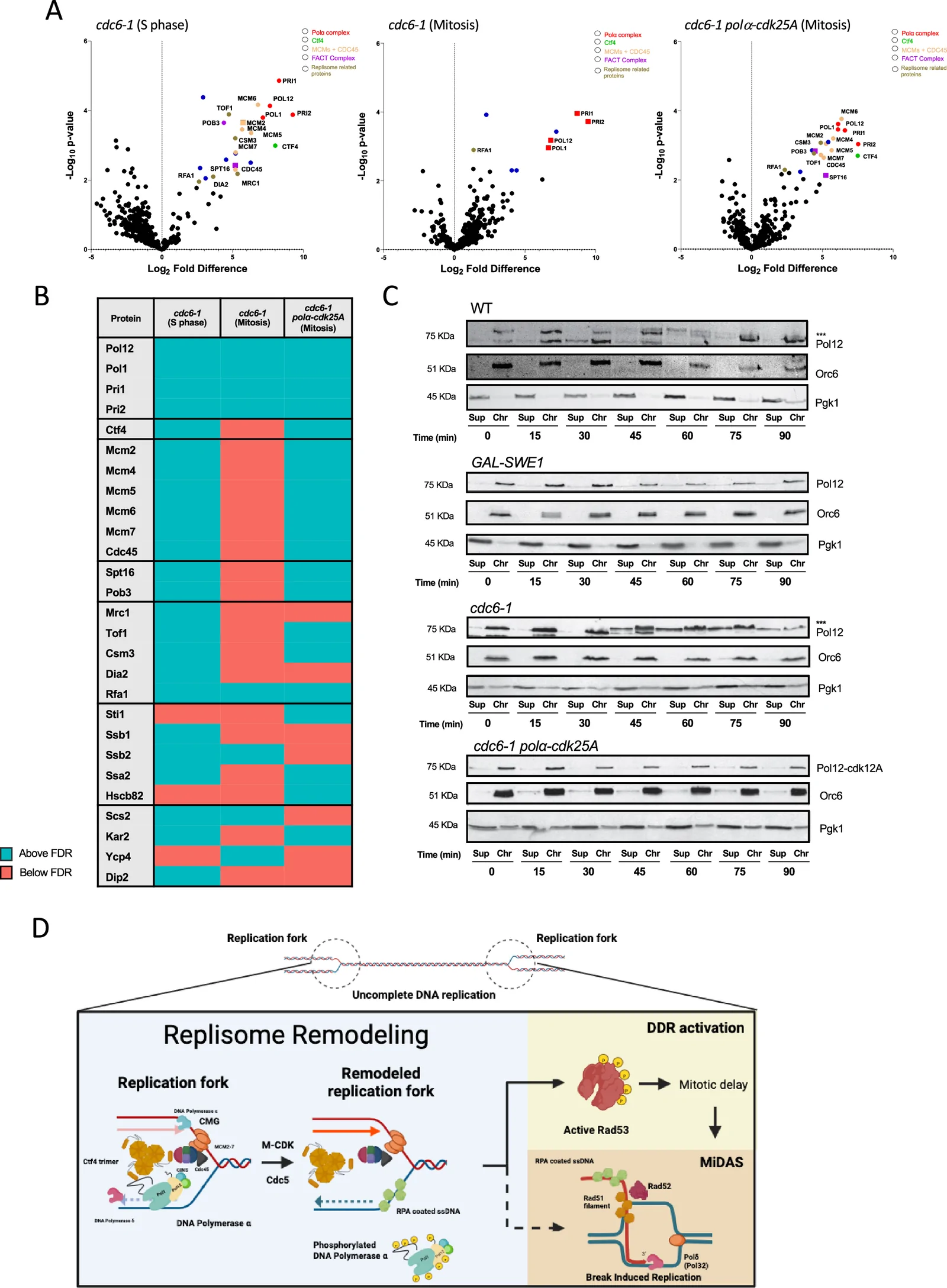
Phosphorylation evicts Polα from chromatin and dissociates it from CMG. (**A**) Quantitative mass spectrometry of Pol1 interactome in *cdc6-1* and *cdc6-1 polα-cdk25A* cells at 30 min (S phase) or 80 min (mitosis) after α-factor release at 30 °C. Volcano plots comparing the immuno-precipitated Pol1 interactome of tagged versus untagged samples in S phase (left) and mitosis (middle), and in mitosis for Pol1-cdk13A (right). The −log10 Benjamini–Hochberg corrected *P* value is plotted against the log2 (fold change: tagged/untagged samples). Colored dots represent statistically significantly enriched proteins with FDR <0.05 (*n* = 3; Raw *p* values were calculated using statistical analysis by *t*-tests; replisome components are indicated). (**B**) Summary of the interactomes of Pol1 and Pol1-cdk13A in S phase and mitosis (FDR <0.01; *n* = 3). (**C**) Pol12 chromatin-binding assay in WT, *GAL-SWE1*, *cdc6-1* and *cdc6-1 polα-cdk25A* cells after release from α-factor at 30 °C (Chr chromatin-bound fraction, Sup soluble fraction). Orc6 and Pgk1 are controls for chromatin and soluble fractions, respectively. (**D**) Model: under-licensed cells or regions poor in origins (e.g., common fragile sites) will still have a small number of active replication forks at the time of mitotic entry. Mitosis is not prevented because these forks are progressing unhindered without activating the DNA replication or DNA damage checkpoint. M-CDK and Cdc5 phosphorylate and evict Polα from the replisome, triggering the DDR and cell cycle arrest in metaphase. This provides time for completion of DNA synthesis by recombination-dependent replication (BIR/MMIR/FoSTeS) evidenced as MiDAS, at the expense of genome stability. Source data are available online for this figure.

To investigate how DNA Polα phosphorylation triggers the DDR, we identified by quantitative MS/MS the interactome of Pol1 during S phase and mitosis in *cdc6-1* and *cdc6-1 polα-cdk25A* cells grown at 30 °C (Fig. 6A). The Polα-primase complex (Pol1, Pol12, Pri1, and Pri2) remained as a holo-enzyme in all conditions. During S phase, Polα bound tightly Ctf4, the Mcm2-7 complex, Cdc45, the FACT complex (Spt16 and Pob3) and the fork protection complex (Mrc1, Tof1, and Csm3), as expected (Gambus et al, 2009; Jones et al, 2023; Simon et al, 2014). Most of these proteins lost their interaction with Polα in mitosis, i.e., when Polα is phosphorylated, but not in cells containing non-phosphorylable Polα-cdk25A (Fig. 6A,B). This indicates that M-CDKs remodel replication forks, causing the dissociation of Polα from the MCM helicase and associated proteins. Crucially, preventing Polα phosphorylation by M-CDK was sufficient to preserve these interactions in mitosis, which demonstrates that a critical role of M-CDK is regulating the association of DNA Polα to CMG and the replisome.

It has been shown that DNA Polα is bound to chromatin only during G_1_ and S, with M-CDK potentially releasing it in mitosis, but no clear function was linked to this regulation (Desdouets et al, 1998). We tested the binding of DNA Polα to chromatin using cellular fractionation in synchronized WT, *GAL-SWE1*, *cdc6-1*, and *cdc6-1 polα-cdk25A* cells. As predicted, Pol12 was released from chromatin concomitantly with its mobility shift (45 min after G_1_ release) in WT cells (Fig. 6C). In cells arrested in G_2_ by overexpression of *SWE1*, Pol12 was neither upshifted nor evicted from chromatin. In *cdc6-1* cells, Pol12 was upshifted and released from chromatin during the long metaphase arrest. Finally, Pol12-cdk12A was never upshifted nor released from chromatin, regardless of the cell cycle phase (Fig. 6C). Altogether, we conclude that DNA Polα is released from chromatin during prophase-metaphase, in a manner completely dependent on its phosphorylation by M-CDK. This then leads to uncoupling from CMG, exposure of ssDNA, RPA and Rad52 binding, checkpoint activation and prolonged metaphase arrest. This long arrest then allows cells to complete DNA synthesis by MiDAS using a BIR-related recombination-dependent mechanism (Fig. 6D).

## Discussion

In this study, we provide a mechanistic understanding of why and how late-replicating loci evolve faster than regions replicated early during S phase. We show that yeast cells with under-licensed origins undergo an extended S phase, not eliciting checkpoints and progress into mitosis with incompletely replicated chromosomes. M-CDK then triggers Polα phosphorylation, its detachment from CMG, ssDNA-RPA accumulation, potent DDR activation and prolonged metaphase arrest during which cells switch from canonical to recombination-dependent DNA synthesis using a Pol32- and Rad51-dependent BIR-related mechanisms. This salvage mode of DNA replication takes place in early mitosis and is relatively efficient as it can replicate a substantial fraction of the genome (30% in our study), yet slowly and at the expense of genome stability. We uncovered this mechanism by extending S phase globally in all cells, but we consider that the same may occur in normal cells at a low frequency due to the stochastic nature of origin firing, or more frequently in cells that have an accelerated G_1_ transit, including cancer cells, and licensing defects (Ekholm-Reed et al, 2004; Lengronne and Schwob, 2002; Tanaka & Diffley, 2002). Allowing the duplication in early mitosis of late-replicating loci using mutagenic processes might be the physiological role of recombination-dependent replication and MiDAS, as well as a driving force for SV in genomes (Epum and Haber, 2022; Wu and Malkova, 2021).

### Origin firing efficiency shapes chromosome replication timing profiles

We have performed a quantitative and global assessment, using single-molecule analysis by DNA combing and nanopore-BrdU sequencing, of the consequences for DNA replication of partial inactivation of licensing factors in yeast. An unexpected finding is that origin under-licensing flattens the replication timing profiles shared by most cells in the population (Fig. 1F) (Müller et al, 2014; Raghuraman et al, 2001). Partial Cdc6 inactivation lowers the activation rate of efficient origins (high peaks), and consequently increases the firing probability of inefficient origins (lower peaks) in early S. We surmise that the increased distance between active origins, due to under-licensing, gives more time and increases the probability of firing for late/dormant origins, independently of any checkpoint effect, as suggested previously by deleting specific origins (Vujcic et al, 1999). Redistribution of firing events between rDNA and single-copy origins has also been observed in *sir2* and *rpd3* mutants, illustrating the high plasticity of origin usage (Yoshida et al, 2014). Similar erosions of replication timing profiles have been observed in yeast cells lacking Rif1, containing an Orc4 subunit unable to bind the ARS-consensus sequence (ACS), or containing humanized histones (Hayano et al, 2012; Lazar-Stefanita et al, 2026; Lee et al, 2021). This indicates that replication timing profiles are sensitive to chromatin context and the ability to bind ORC or load the pre-RC at preferred sites in the genome. The depth and high S/N ratio of our nanopore BrdU sequencing method revealed that so-called ‘late’ origins in fact do fire at low frequency in early S phase (HU-arrested cells; Fig. EV1A), suggesting that they are not late by nature but simply inefficient and thus require more time to show up in bulk experiments. We propose that origin firing efficiency is a key determinant of replication timing profiles in populations, consistent with single-cell measurements (Massey and Koren, 2022). Since replication timing domains overlap with A/B genome compartmentalization and transcriptional activity (Pope et al, 2014), it would be interesting to see if the loss of coherence in genome replication timing in under-licensed cells affects gene expression and developmental processes.

### Origin under-licensing: a stealth inducer of genome instability

The overwhelming majority of studies on the maintenance of genome integrity were performed by inflicting various kinds of DNA damage or impediments to fork progression. All these treatments slow or stop replication forks, uncouple DNA polymerases from CMG helicase, and generate extended stretches of RPA-coated ssDNA that recruit and activate a potent ATR-CHK1 (Mec1-Rad53 in yeast) replication stress response. This in turn promotes protection and repair strategies while preventing CDK1 activation and mitotic entry until the problem is fixed (Byun et al, 2005; Saldivar et al, 2017). Unperturbed DNA replication also delays mitosis in yeast and human cells (Lemmens et al, 2018; Magiera et al, 2014; Saldivar et al, 2018), but this is signaled differently to stressed forks and requires a threshold of active forks (Mocanu et al, 2022; Shimada et al, 2002). In contrast, origin under-licensing extends S phase by increasing the distance between active origins (Devault et al, 2002; Lengronne and Schwob, 2002) and lowers the number of normally progressing forks below the threshold needed to delay mitosis, particularly towards the end of S phase or in G_2_. This allows cells to enter mitosis with partly unreplicated DNA, undetected by checkpoints, with mitotic kinases causing replisome disassembly and chromosome pulverization (Crasta et al, 2012; Deng et al, 2019), as showcased by CFS that are very-late-replicating sequences stemming from origin paucity (Letessier et al, 2011). Interestingly, yeast cells lacking the main S-phase cyclin Clb5 and thus replicating their genome from only a subset of origins display a 700-fold increase in large segmental duplications mediated by mechanisms similar to those described here (Payen et al, 2008). That *clb5∆*, *cdc6-1*, and *sic1∆* cells with extended S phase have high rates of SD/GCR, while *clb5,6∆* cells with delayed S phase but normal number of forks do not (Fig. 3D; Lengronne and Schwob, 2002), is consistent with the notion that completing genome duplication with a few active forks escapes detection by surveillance mechanisms. We conclude that origin under-licensing jeopardizes genome integrity by allowing cells to activate M-CDK and enter mitosis with under-replicated chromosomes.

### Polα phosphorylation leads to DDR activation and provides time for a change

We show here that M-CDKs phosphorylate DNA Polα on its two large subunits (Pol1, Pol12), which causes its physical dissociation from the CMG helicase (Fig. 6A) and potent DDR activation in cells that have not yet completed chromosome replication (Figs. 2B and 5A). Polα is the main or sole target of M-CDKs in this pathway since non-phosphorylable Polα mutants remain bound to CMG in mitosis (Fig. 6B) and do not activate the DDR (Fig. 5A). Our study uncovers a new role for Polα phosphorylation, which is to trigger the DDR by its eviction from the replisome at the G_2_/M transition, thus providing time for cells to switch to a recombination-dependent mode of replication (BIR/FoSteS) and finish chromosome replication in early mitosis (MiDAS) before anaphase (Movie EV1). Importantly, non-phosphorylable Polα mutants are viable and proficient for canonical replication in otherwise WT cells, but lethal in under-licensed cells that rely on MiDAS for the completion of genome duplication. It is interesting that Polα phosphorylation causes DDR activation, as seen for stalled forks during S phase, but in this case only after mitotic entry and without first requiring fork stalling. A key difference is that DNA polymerases remain bound to chromatin at stalled forks during S phase and are only transiently uncoupled from CMG, to allow fork restart after resolution of the problem (Pabba et al, 2023). In contrast, Polα is physically evicted from chromatin/CMG when cells enter mitosis with under-replicated DNA, allowing the switch to a salvage mode of replication. Although we cannot fully rule out a direct role of Polα phosphorylation and eviction from the replisome in promoting BIR-related DNA synthesis, the fact that the lethality of *cdc6-1 polα-cdk25A* cells can be suppressed by delaying anaphase (Figs. 5F and EV6D) argues for DDR activation being a primary role of Polα phosphorylation (Fig. 6D).

### Evolutionary conservation and role of Polα phosphorylation

Most Pol1 and Pol12 phosphorylation sites reside within their N-terminal intrinsically disordered regions (IDR). Genetic dissection and cryo-EM structural analysis of the replisome indicate that the Pol1 and Pol12 IDRs are in proximity and may interact with Ctf4 (human AND-1) and Mcm3, tethering Polα to the replisome (Jones et al, 2023; Villa et al, 2016). We propose that Polα phosphorylation loosens its interaction with Ctf4 and MCM, causing its eviction from CMG and activation of the DDR by generating ssDNA on the lagging strand. This regulation may be evolutionarily conserved since the two largest subunits of Polα from human and plants are also phosphorylated within their N-ter IDRs. It was shown that phosphorylation of human POLA1 and POLA2 does not affect polymerase and primase activities in vitro, but that phosphorylation by CycA-Cdk2 or CycA-Cdk1 (not CycE-Cdk2) in G_2_ strongly inhibits the ability of Polα to initiate SV40 DNA replication (Schub et al, 2001; Voitenleitner et al, 1997), by preventing its interaction with SV40 large T antigen via phosphorylation of three CDK sites on POLA1 and four sites on POLA2 (Dehde et al, 2001). The same authors also show that hypo-phosphorylated Polα colocalizes with and binds Mcm2, but not when it is hyper-phosphorylated. Thus, it is likely that phosphorylation of the IDRs of POLA1 and POLA2 disrupts their interaction both with T Ag and CMG helicases, possibly by using Ctf4/AND-1 or other interfaces.

### Polα phosphorylation and CMG disassembly

The active MCM hexamer encircles the leading strand ssDNA template and remains stably bound to ensure fork restart and faithful genome replication. CMG is unloaded only upon termination of replicon synthesis when two converging forks pass each other, which allows Mcm7 ubiquitination by SCF^Dia2^ (CRL^Lrr1^ in *Xenopus*, *C. elegans* and humans) and CMG disassembly by the p97/Cdc48 segregase (Maric et al, 2014). A second ubiquitin ligase system, TRAIP, can compensate for the absence of CRL^Lrr1^ in animal cells. In *Xenopus* egg extracts, high CycB-Cdk1 activity causes CMG unloading via TRAIP at persistently stalled forks, creating DSBs, abnormal replication products and deletions via MMEJ independently of Rad51 (Deng et al, 2019). In *C. elegans*, TRAIP unloads CMG at terminated forks in prophase if CRL^Lrr1^ failed to do so at the end of S phase. We find unlikely that Polα phosphorylation and TRAIP serve redundant functions for CMG disassembly, for several reasons. First, Polα and CMG behave independently since the latter remains bound to chromatin and continues unwinding DNA when the levels of Polα are reduced, or its activity is inhibited, which results in uncoupling of Polα and CMG (Byun et al, 2005; Ercilla et al, 2020; Hoffman et al, 2021; Walter and Newport, 2000). Second, to our knowledge, TRAIP has not been shown to function on normally progressing replication forks. Third, CMG unloading via Mcm7 ubiquitylation is not conserved in plants, and evolved several times independently, unlike Polα phosphorylation which is conserved. Fourth, Mcm7 ubiquitylation and CMG unloading are not essential for mitotic exit and viability in yeast, since CMG complexes left on DNA from the previous cell cycle in *dia2∆* or *mcm7-10R* mutants are removed by Pif1/Rrm3 helicases in the subsequent S phase (Polo Rivera et al, 2024). In contrast, Polα phosphorylation and eviction from CMG is essential for cells with an extended S phase and necessary for replication completion by BIR/FoSTeS, which causes rapid genome evolution and favors adaptation.

In summary, we show that origin under-licensing is a key determinant of genome instability as it causes late DNA replication undetected by checkpoints, mitotic entry with under-replicated DNA, and completion of DNA synthesis in prophase using mutagenic BIR-related mechanisms. Polα phosphorylation plays a key role as it stops canonical DNA replication and triggers a strong DDR that provides time for launching recombination-dependent replication. We envision that cancer cells, which often display a truncated G_1_ and potentially impaired origin licensing, may utilize Polα phosphorylation and MiDAS/BIR as drivers of their genome instability. Our study also provides a mechanistic framework explaining the natural hyper-mutability of late-replicating loci and their role in evolution.

### Limitations of the study

We have pre-emptively mutated all S/T-P sites to alanine in Pol1 (13 sites) and Pol12 (12 sites), and showed that this mutated version of Polα is fully proficient, at endogenous levels, for canonical replication in wild-type cells, but unable to activate the DDR and sustain a metaphase arrest in cells replicating their DNA very late due to origin under-licensing. We found that many but not all of these sites are phosphorylated in vivo in mitosis, and that other sites are also phosphorylated. We failed so far to identify the minimal set of Polα phospho-residues required for DDR activation. Although we make analogies with human CFS and MiDAS, the situation is clearly more complex in mammals than in yeast, and we note discrepancies such as the apparent absence of DSBs in under-licensed yeast cells performing MiDAS and their independence on Mus81-Mms4 nuclease for DDR activation. Further studies are required to test the role of Polα phosphorylation in human cells.

## Methods

**Table:** Reagents and tools table

Reagent/resource	Reference or source	Identifier or catalog number
**Experimental models**		
*Saccharomyces cerevisiae*	This study (Table EV2)	W303 *RAD5* (if not stated otherwise)
**Recombinant DNA**		
*pol1-cdk13A*	This study—Appendix Fig. 4	D1969/pML151
*pol12- cdk12A*	This study—Appendix Fig. 5	D2233 (in pRS306)
Cas9-KanMX guide plasmid	G. Zhao and B. Futcher	D2234
**Antibodies**		
Mouse anti-ssDNA	Chemicon	MAB3034 (1:300)
Rat anti-BrdU	AbCys	OBT0030 (1:20)
Anti-mouse Alexa 546	Molecular Probes	A-11030 (1:50)
Anti-rat Alexa 488	Molecular Probes	A-11006 (1:50)
Rabbit anti-Pol12 mAb	Marco Foiani	Clone 6D2 (1:1000)
Mouse anti-Rad53 mAb	Marco Foiani	Clone EL7 (1:20)
Rabbit anti-Swi6 Ab	Kim Nasmyth	R12 (1:100,000)
Rabbit anti-Sic1 Ab	Mike Tyers	(1:5000)
Rabbit anti-Clb2 Ab	Kim Nasmyth	R98.12 (1:2000)
Mouse anti-Orc6 mAb	Steve Bell, John Diffley	Clone SB49 (1:1000)
Mouse anti-PK mAb	Serotec	MCA1360 (1:2000)
Mouse anti-Pgk1 mAb	Invitrogen	459250 (1:2500)
Anti-mouse IgG-HRP	Sigma	A9044 (1:25,000)
Anti-rabbit IgG-HRP	Sigma	A9169 (1:30,000)
Anti-mouse IgG-IR680	Thermo	35518 (1:20,000)
Anti-rabbit IgG-IR800	Thermo	35571 (1:20,000)
**Oligonucleotides and other sequence-based reagents**		
**Chemicals, enzymes and other reagents**		
Alpha-factor pheromone	Genescript	WHWLQLKPGQPMY
Pronase	Calbiochem	53702
RNaseA	Sigma	R5000
Proteinase K	Euromedex	EU0090
Sytox Green	Life Technologies	S-7020
EdU	Carbosynth	NE08701
BrdU	Sigma	B-5002
DY-530 azide	Dyomics	530-10
Cy5 azide	Lumiprobe	A3030
Ascorbic acid	Sigma	A4544
Propidium iodide	Sigma	P4864
DAPI	Sigma	D9542
Hydroxyurea (HU)	US Biologicals	NB-42-01417
Zymolyase	Roth	9324.3
Protease inhibitor cocktail	Roche-Sigma	04693132001
Benzonase	Thermo	88702
Magnetic beads	Dynabeads	MAN0015803
Lambda phosphatase	NEB	P0753S
All other standard chemicals	Sigma	
**Software**		
Minimap2	https://github.com/lh3/minimap2	
DNAscent	Jones et al, 2025	v3.1.2
**Other**		
Nanopore sequencing	Oxford Nanopore Technologies	FLO-MIN106D, FLO-MIN114D
Rapid Barcoding kit	Oxford Nanopore Technologies	SQK-RBK114.24
Genomic DNA purification	Qiagen 20 G	10223

### *POL1, POL12* tagging, and mutagenesis

The *POL1*, *pol1-cdk13A*, *POL12*, and *pol12*-*cdk12A* (promoter, ORF and terminator) were cloned into a pRS316 plasmid. The 3xPK was inserted in frame at the 3’end of the ORF of each gene. Each construct was introduced at the *POL1* or *POL12* locus by co-transforming E5729 (*POL1*-6HA::KanMX4) or E5739 (*POL12*-6HA::KanMX4) with a *LEU2* selectable plasmid encoding both Cas9 and gRNA against the KanMX sequence (kindly provided by G. Zhao and B. Futcher) and a PCR fragment spanning the *POL1* and *POL12* constructs. Transformants were selected on -Leu SC medium. Transformants were sequenced for the correct introduction of the 3xPK sequence and the designed mutations and the preservation of the remaining part of the targeted gene. *POL1* and *POL12* sequences with their mutations of consensus CDK sites and diagnostic restriction sites are shown in Appendix Figs. S4 and S5.

### Yeast strains and cultures

All strains used in this study are listed in Table EV2 and were constructed using standard genetic methods. YEP and SC liquid media were supplemented with 2% glucose unless otherwise indicated. For cell cycle experiments, asynchronous cells were grown at 25 °C in YPD or SC medium to 2–3 × 10^6^ cells/mL and α-factor (2 μg/mL) was added for 1 h and 5 min twice. This step was done at 30 °C in the *cdc6-1* mutant to ensure partial inactivation of the Cdc6 protein during the licensing step in G_1_. Then, cells were released into the cell cycle by removing α-factor either by centrifugation (1500 rpm for 3 min) and resuspension in α-factor-free media (small cultures) or by adding pronase (50 μg/mL, large cultures). For the experiments scoring mitotic exit, α-factor (10 μg/mL) was added to the cell culture 45 min after release to arrest cells in the following G_1_.

### DNA content analysis by flow cytometry

Cellular DNA content was measured by FACS as described in (Barba Tena et al, 2022). Briefly, cells were collected at different times after release from G_1_, fixed with 10% EtOH and permeabilized with 70% EtOH. Afterward, cells were treated with 0.1 mg/ml RNAseA and 0.2 mg/ml Proteinase K, stained with 0.5 μM Sytox Green and read using a 488 nm excitation laser and 530/30 BP filter on a Novocyte Acea.

### In vivo EdU incorporation and click-it reaction

Genetically-engineered yeast overexpressing hENT1 (human Equilibrative Nucleoside Transporter) and TK (Thymidine Kinase) were grown in SC media, pulsed with 10 µM EdU for 3 to 10 min, immediately fixed in 2% PFA, and then permeabilized with 70% EtOH. Cells were incubated in PBS–1% BSA, washed in PBS, resuspended in 38.2 µl of Click-iT reaction mix (2 mM CuSO_4_, 20 µM DY-530 or 10 µM Cy5 azide, and fresh 10 mM ascorbic acid in PBS) and left for 30 min in the dark. Cells were washed three times with 10% ethanol in PBS and incubated with 0.5 µg/ml DAPI for microscopy analysis or with 50 µg/ml propidium iodide for bivariate-FACS analysis (Talarek et al, 2015).

### Measurement of cell cycle phase duration

The duration of different cell cycle phases was determined as described in (Barba Tena et al, 2022; Talarek et al, 2015). Briefly, cells growing exponentially were used to measure their doubling time (DT), and pulse-labeled for 5 min with 10 μM EdU. The fraction (F) of cells in G_1_, S and G_2_ + M phases was determined using bivariate PI-EdU FACS. The duration of each phase was calculated by DT x F.

### MiDAS detection by quantitative image-based cytometry

Synchronized *cdc6-1* single and double mutant cells grown at 30 °C were labeled 90 min after G_1_ release for 10 min with 50 μM EdU (or DMSO), fixed, permeabilized and a click reaction was performed as described above using Cy5-azide. Cells were then stained with 0.5 µg/ml DAPI, sonicated and analyzed on an ImageStream X MKII instrument (operated by the ISX software) with a magnification of 60X, taking images of Brightfield, DAPI and EdU using the lasers 405 nm (Violet—DAPI) and 640 nm (red—EdU/Azide). Every set of mutants was treated and acquired the same day to avoid technical variability, with 10,000 cells acquired per sample. Image analysis was performed using the IDEAS software. The percentage of MiDAS-positive cells and intensity of EdU signal per nucleus was extracted and analyzed with GraphPad Prism and RStudio. The following workflow was used to identify MiDAS-positive cells (Fig. EV3A): (i) focused cells were selected based on the Gradient_MS_BF parameter; (ii) mitotic cells (dumbbell cells) were selected based on circularity, selecting only “elongated cells” using the parameters Area_BF and Aspect_Ratio_Intensity_BF; (iii) EdU/MiDAS-positive cells were identified based on the presence of EdU foci, using the “peak mask” and “spot counting feature” that calculate the difference between a pixel and those nearby; (iv) to discard false positives, only cells where EdU signal colocalizes completely with DAPI signal were selected, thus excluding cells with extranuclear EdU foci (e.g., mitochondria), using the Bright_Detail_Similarity_R3_EdU_DAPI parameter.

### Fluorescence microscopy

Microscopy analysis was performed as described in (Magiera et al, 2014). Briefly, cells were grown in SC medium, centrifuged, resuspended in 90 μL PBS, and fixed by the addition of 10 μL 80% EtOH. If required, nuclear staining was performed by incubating cells with 0.5 μg/mL DAPI for 30 min in the dark and washing once with PBS. Then, the cell suspension was briefly sonicated and concentrated to the desired density. 1.7 μL cell suspension was put on a microscope slide and observed using Leica Thunder or Zeiss AxioImager microscopes (operated with LasX or Zen software) using a 100X objective (Apochromat 0.7–1.4 NA oil) with the adequate lasers and filters (Brightfield, EdU—Cy3, DAPI—Hoechst, Rad52-GFP, RPA—GFP, Tubulin—Texas Red). Image analysis was performed using the FIJI or ImageJ software. A minimum of 100 cells were scored per timepoint after sample anonymization and repeated by two different experimenters.

### Live-cell imaging

Long-term video-microscopy was performed on a CSU-X1 Andor Spinning Disk instrument equipped with 488 nm (60 mW) and 561 nm (50 mW) solid-state lasers and an iXon Ultra emCCD camera, as described in (Montecchi and Schwob, 2018). Briefly, diploid cells were grown in SC-D medium and placed on a 35 mm FluoroDish under a preheated SC-D agar pad supplemented with 10 mM Tris pH 7.5 to preserve GFP stability, and immediately imaged with 100X/1.45 objective at the desired temperature with the minimal laser power required to see Tub1-mCherry and Rad52-GFP. 11 z-stacks with 0.67 µm steps were taken every 1 or 2 min for 4–12 h and images processed using Fiji with Pure Denoise.

### DNA combing analysis

For DNA combing, yeast strains expressing thymidine kinase (TK) and human nucleoside transporter (hENT1) were grown in YPD at 25 °C and arrested for 2 h with α-factor at different temperatures. 50 μM BrdU and 200 mM HU were added 30 min prior to release. Cells were then filtered to eliminate α-factor and resuspended in YPD (50 μM BrdU, 200 mM HU) at different temperatures. Cells were collected at the indicated times and treated for genomic DNA purification and DNA combing as described (Schwob et al, 2009). DNA was counterstained with a mouse anti-ssDNA antibody (MAB3034, Chemicon, 1:300) and BrdU detected with a rat anti-BrdU antibody (OBT0030, AbCys, 1:20), revealed respectively with anti-mouse Alexa 546 and anti-rat Alexa 488 (Molecular Probes, 1:50). DNA fiber images were acquired on a Leica DMRA microscope equipped with a Photometrics CoolSnap CCD camera, and analyzed using MetaMorph and GraphPad Prism software. More than 200 fibers longer than 100 kb were analyzed on average for each timepoint, and the experiment was repeated three times by two different researchers.

### Protein extraction and Western blot

About 10–15 μg whole-cell extracts were run on 8–15% acryl–bisacrylamide gels. Proteins were transferred to nitrocellulose membranes (Protran; Whatman) using wet blotting and revealed using standard immunoblotting and ECL procedures. Primary antibodies were rabbit monoclonal anti-DNA Polα B subunit (Pol12; 1:1000; clone 6D2; obtained from Marco Foiani), mouse monoclonal anti-Rad53 (clone EL7; 1:20; obtained from Marco Foiani), rabbit polyclonal anti-Swi6 (R12; 1:100,000), mouse monoclonal anti-PK (1:2000, Biorad-Serotec MCA1360), rabbit polyclonal anti-Sic1 (1:5000 gift from Mike Tyers), rabbit polyclonal anti-Clb2 (1:2000 from Kim Nasmyth’s lab, R98.12), mouse monoclonal anti-Orc6 (1:1000, clone SB49 from Steve Bell and John Diffley labs), mouse monoclonal anti-Pgk1 (1:2500, Invitrogen 459250). Secondary antibodies were anti-mouse IgG-HRP (1:25,000, Sigma A9044), anti-rabbit IgG-HRP (1:30,000, Sigma A9169), anti-rabbit IgG-IR800 (1:20,000, Thermo SA5-35571) and anti-mouse IgG-IR680 (1:20,000, Thermo 35518).

### Cellular fractionation assay

The procedure for chromatin purification is based on the method described by Desdouets et al, 1998. Briefly, cells were arrested with α-factor at 2–3 × 10^6^ cells/mL, released into the cell cycle and aliquoted at different timepoints. Cells were resuspended sequentially in Solution I (100 mM Pipes-KOH pH 9.4, 10 mM DTT for 10 min at 30 °C), Solution II (50 mM KPO4, pH 7.5, 0.6 M sorbitol, 10 mM DTT, 0.5 μg/μL Zymolyase for 30 min at 30 °C) and Solution III (0.4 M Sorbitol, 150 mM potassium acetate, 2 mM magnesium acetate, 20 mM Pipes-KOH, pH 6.8, Protease Inhibitor 1X, 1% Triton X-100). The chromatin-enriched fraction was isolated after centrifugation for 15 min at 15,000×*g* on a 30% sucrose cushion, and the supernatant was carefully removed (soluble fraction).

### DNA Polα immunoprecipitation

Native protein extract was performed as previously described in (Gambus et al, 2006) with small variations. For each sample, 10^10^ cells were harvested and washed twice with 20 mM HEPES pH 7.9 and once with Lysis Buffer (100 mM HEPES, 50 mM Potassium Acetate, 10 mM Magnesium Acetate and 2 mM EDTA) and resuspended in Supplemented Lysis Buffer (Lysis Buffer containing 2 mM Sodium Fluoride, 60 mM β-glycerophosphate, 1 mM DTT, 1% Protease Inhibitor Cocktail and 1 mM o-vanadate) in proportion of 1 mL per gram of material and snap-frozen in liquid nitrogen. Yeast popcorn was broken using a Freezer Mill 6770 (15 CPS, 1.5 min, two cycles) and supplemented with 25% of the total volume with Glycerol Mix (100 mM HEPES, 300 mM potassium acetate, 10 mM magnesium, 50% glycerol, 0.5% IGEPAL, 81 mM β-glycerophosphate, 1 mM DTT, 1% Protease Inhibitor Cocktail, 1 mM o-vanadate, 40U Benzonase) for 30 min at 4 °C. Finally, two clarifications were performed by centrifugation at 25,000×*g* for 30 min at 4 °C.

For Pol1-3PK and Pol12-3PK immunoprecipitation, native protein extracts were incubated with magnetic beads (Dynabeads, MAN0015803) coupled to anti-PK antibody (Biorad-Serotec MCA1360) for 2 h at 4 °C. Beads were washed four times with 2 mL Wash Buffer (100 mM HEPES, 100 mM Potassium Acetate, 10 mM Magnesium Acetate, 0.1% IGEPAL, 2 mM sodium fluoride, 81 mM β-glycerophosphate, 1% Protease Inhibitor Cocktail, and 1 mM o-Vanadate) and eluted differently depending on the assay. For phosphosite mapping, beads were eluted by adding 50 µL 1X Laemmli buffer and boiled 5 min at 95 °C. For interactome analysis, beads were incubated with 0.74 mg/mL V5 peptide during 30 min and the supernatant was boiled with 1X Laemmli Buffer 5 min at 95 °C. For phosphatase assays, beads were subjected to λ phosphatase reaction (3 μL PMP Buffer, 3 μL λ PPase, and 3 μL MnCl_2_, NEB P0753S) for 30 min and the supernatant was boiled with 1X Laemmli Buffer for 5 min at 95 °C.

### Mass spectrometry analysis

Immunoprecipitated samples were separated by gradient SDS-PAGE, and bands of interest were cut out and resuspended in 10 μL Buffer A (0.1% formic acid). About 2 μL were injected in an HPLC (RSLC U3000, Thermo Fisher Scientific) coupled to a mass spectrometer equipped with a nanoelectrospray (Q Exactive HF, Thermo Fisher Scientific). Peptides were separated in a capillary column (0.075 mm × 500 mm, Acclaim Pepmap 100, Thermo Fisher Scientific) followed by a gradient separation in buffer B (range 2–40%, 0.1% formic acid, and 80% acetonitrile) at 300 nL/min. The MS spectra were registered using Xcalibur 4.1 software with 128LowQtt.meth protocol and analyzed using MaxQuant. Two databases were used to determine contaminants and yeast proteins (contaminants_fpp_180320.fasta, RefProteome _YEASTcano_2020_02_ UP000002311.fasta). For phosphorylation mapping, all analyses from phospho-peptide to phospho-site identification were done with a fixed value of 1% FDR. For the interactome, peptide identification was done with a fixed value of 1% FDR and graphs and statistical tests were done with Perseus Software. Immunoprecipitation from a non-PK tagged strain was used as a negative control and analysis were performed on three biological replicas per sample (total of 12 samples).

### Direct BrdU-nanopore sequencing

Yeast strains expressing TK and hENT1 were grown in YPD, synchronized with α-factor and released into the cell cycle. For replication profiles, 100 µM BrdU and 200 mM HU were added 15 min prior to release, cells were washed 50 min after release and resuspended in YPD media containing 200 µM thymidine for chase and 15 µg/mL nocodazole to arrest cells in mitosis. For MiDAS profiles, cells were released from α-factor arrest and 100 µM BrdU was added only after cells had entered mitosis (80 min after αF release) and collected at 135 min. Genomic DNA was purified using Qiagen 20G yeast genomic DNA kit following the manufacturer’s instructions. Sequencing libraries were prepared using the Rapid Barcoding kit (ONT, SQK-RBK114.24) and sequenced with R9.4.1 or R10.4.1 flow cells (FLO-MIN106D and FLO-MIN114D). A minimum of 700 Mb (60x coverage) was sequenced per sample. Reads were mapped to our reference genome using Minimap2, sorted using Samtools and BrdU content was calculated using DNAscent v3.1.2 (Jones et al, 2025). The genome was then divided into 500 bp bins, and BrdU content per bin was calculated using the following formula:

$${BrdU}\,{content}=\,\frac{\sum {Positive}\,{reads}}{\sum {Total}\,{reads}}$$

Positive read if more than 50% of k-mers in a window are BrdU positive.

Kmer positive if BrdU% >0.5.

All reagents, providers and references are indicated in the Reagents and Tools table.

## Supplementary information

Appendix

Table EV1

Table EV2

Peer Review File

Movie EV1

Movie EV2

Source data Fig. 1

Source data Fig. 2

Source data Fig. 3

Source data Fig. 4

Source data Fig. 5

Source data Fig. 6

Expanded View Figures

## Data Availability

Nanopore sequencing data generated in this study have been deposited in the ENA database (https://www.ebi.ac.uk/ena/browser/view/PRJEB92099). Mass spectrometry data generated in this study have been deposited to the PRIDE database https://proteomecentral.proteomexchange.org/ui?pxid=PXD065889 for the IP-MS of Pol1 and https://proteomecentral.proteomexchange.org/ui?pxid=PXD065886 for the phospho-MS of Pol1 and Pol12. Microscopy images are deposited on https://www.ebi.ac.uk/biostudies/studies/S-BSST3223. Custom scripts used in this study to analyse the nanopore sequencing data and generate the figures can be found on https://src.koda.cnrs.fr/TDef/ONT_brdu_dnascent_scripts. The source data of this paper are collected in the following database record: biostudies:S-SCDT-10_1038-S44318-026-00919-5.
