# Supplementary material for: Switch from canonical to recombination-dependent DNA synthesis by M-CDK phosphorylation of DNA Polα
Source: EMBO J. 2026 Oct 10;45(21):7621–46. doi: 10.1038/s44318-026-00919-5 (PMC13728163; doi:10.1038/s44318-026-00919-5)
Supplement: Supplementary file 1 [file 44318_2026_919_MOESM13_ESM.pdf]

Expanded View Figures

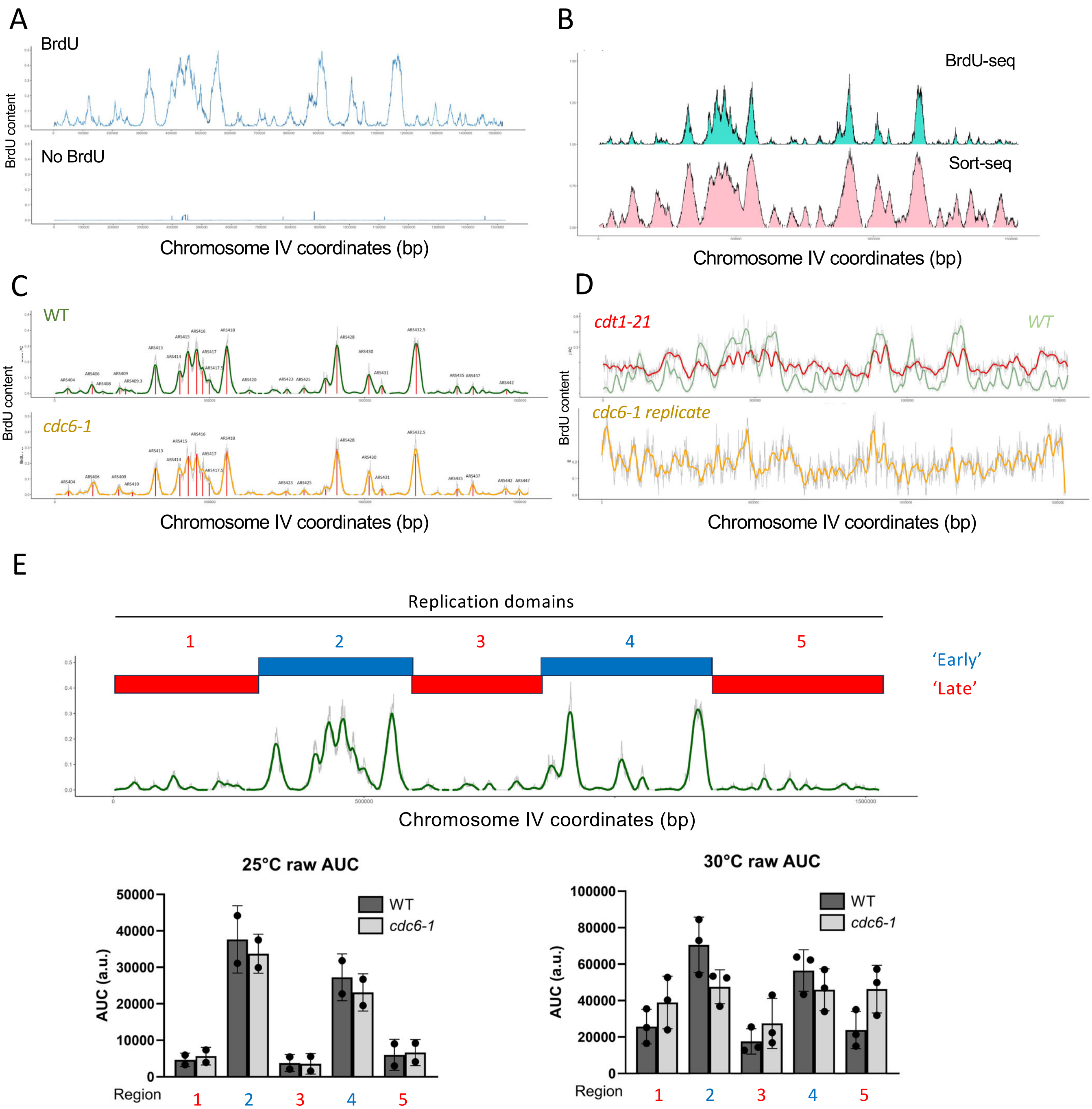

Figure EV1. Validation of nanopore BrdU-seq pipeline (related to Fig. 1).

(**A**) ChrIV replication profiles of WT cells grown in the presence (top) or absence (bottom) of BrdU, using our nanopore BrdU-seq detection pipeline. (**B**) Comparison of ChrIV replication profiles of WT cells obtained either by nanopore BrdU-seq or by copy-number-based Sort-seq method (Müller et al, 2014). Note that late-firing origins are detected in the BrdU-seq method despite using HU, yet at a lower level than in asynchronous cells not exposed to HU, as expected. (**C**) Replication origins identified on ChrIV in WT (green) or *cdc6-1* (orange) cells grown at 25 °C. Origins were called based on the position of BrdU profile peaks (local maxima) and the position of known ARS (OriDB Database). (**D**) ChrIV replication profiles of WT (green) and *cdt1-21* cells (red) grown at 30 °C (top), and a biological replicate of *cdc6-1* cells grown at 30 °C. (**E**) Quantification of BrdU incorporation within early and late replication domains on ChrIV. Profile (top) is WT at 25 °C. Area under the curve (AUC, bottom, mean ± SD) for the shown early and late domains of WT and *cdc6-1* cells grown at 25 °C (left, $n = 2$) or 30 °C (right, $n = 3$).

 © The Author(s)

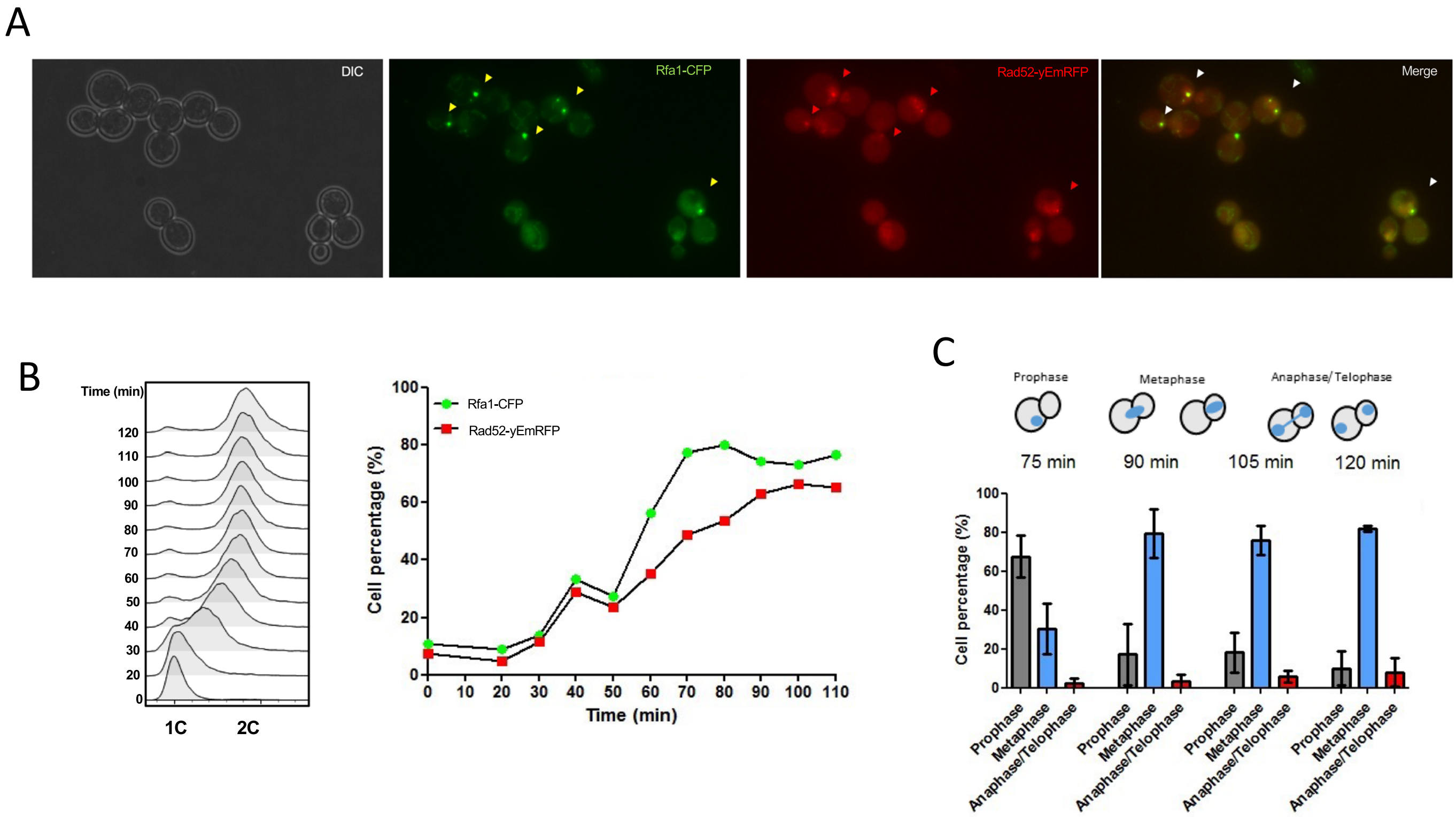

Figure EV2. Spatial and temporal correlation of Rfa1 and Rad52 foci formation in *cdc6-1* cells (related to Fig. 2).

(**A**) Representative images of asynchronous *cdc6-1 RFA1-CFP RAD52-yEmRFP* cells grown at 30 °C for 2 h (DIC, differential interference contrast; scale bar, 5 μm; arrowheads indicate foci). (**B**) Kinetics of Rfa1 and Rad52 foci formation in *cdc6-1* cells synchronized and released from G_1 at 30 °C. DNA content by flow cytometry (left), fraction of cells containing Rfa1 or Rad52 foci (right; $n = 2$, >100 cells counted). (**C**) Accumulation of *cdc6-1* cells in metaphase. (Top) Schematic representing nucleus position relative to bud neck and daughter cell to classify cells in prophase, metaphase, ana/telophase. (Bottom) Fraction of cells in each class at the indicated time after G_1 release $n = 3$, mean ± SD, >100 cells).

© The Author(s)

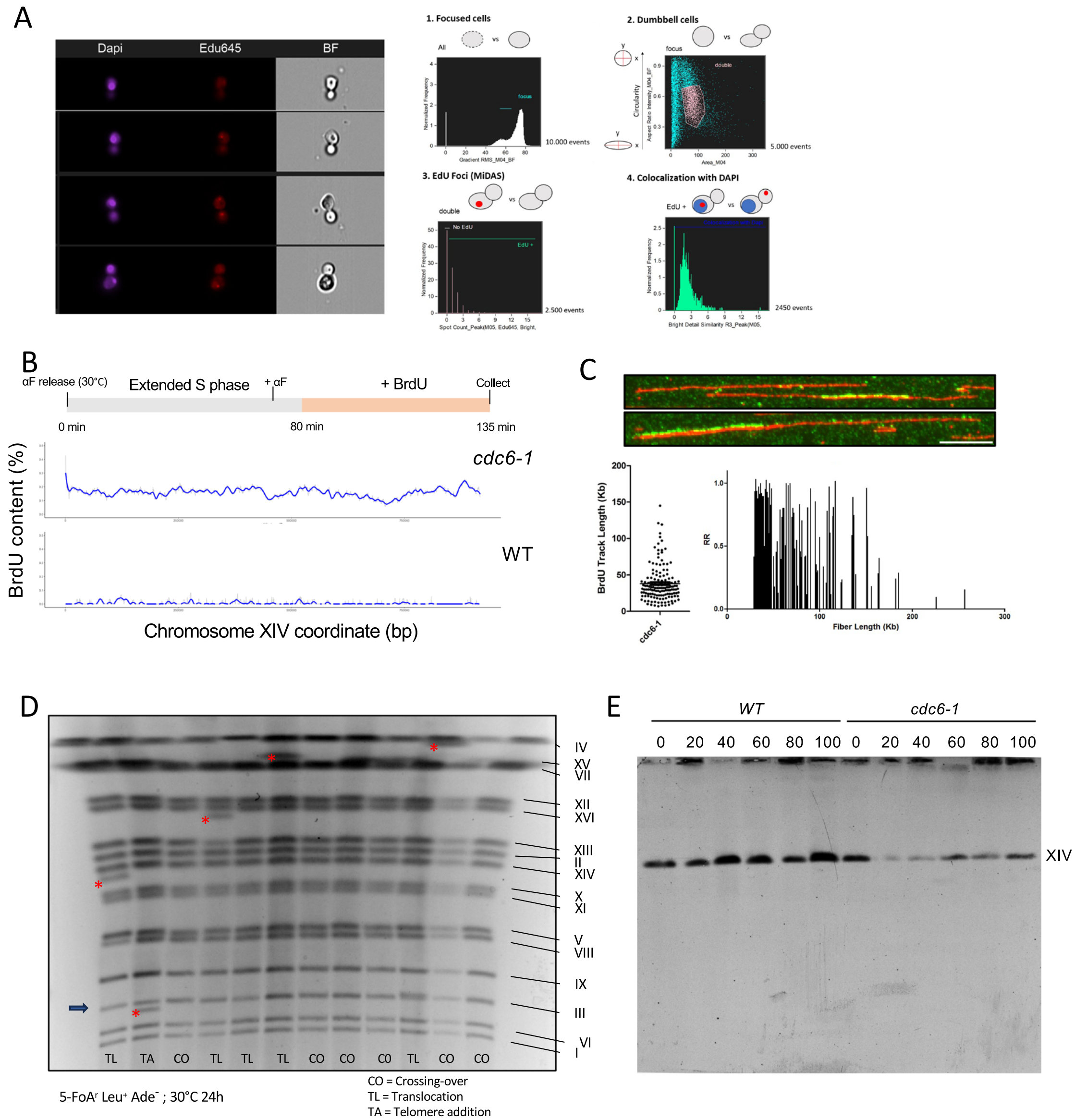

Figure EV3. Mitotic DNA synthesis and chromosome instability in *cdc6-1* cells (related to Fig. 3).

(**A**) QIBC for MiDAS detection in yeast. Representative images of MiDAS-negative, MiDAS-positive cell with single or multiple foci, false-positive cell with extranuclear EdU (left, top to bottom), and QIBC analysis workflow to identify MiDAS cells (right). (**B**) Pan-chromosome XIV mitotic DNA synthesis in *cdc6-1* cells pulsed with BrdU during mitosis (from 80 to 135 min after G_1 release; α-factor re-added at 60 min), relative to WT control. (**C**) Detection of MiDAS BrdU tracts (green) on single DNA molecules (red) stretched by DNA combing (top; bar 10 μM). BrdU tract length for fibers ≥30 kb (left) and BrdU replacement rate (RR) on these fibers. BrdU-labeled DNA fibers are almost fully substituted up to 150 kb. (**D**) PFGE karyotype analysis of spontaneous 5-FoAr Leu+ Ade- clones isolated from diploid *cdc6-1* cells grown at 30 °C for 24 h. Translocations (TL) are told apart from equal crossing-overs (CO) by the decrease in ChrIII band intensity (arrow) and the appearance of a new chromosome band (asterisk). See Hiraoka et al (2000) for details. (**E**) Lack of detectable DSB in *cdc6-1* cells released from G_1 arrest at 30 °C and undergoing MiDAS. PFGE-Southern blot analysis with *POL1* radioactive probe labeling ChrXIV.

 © The Author(s)

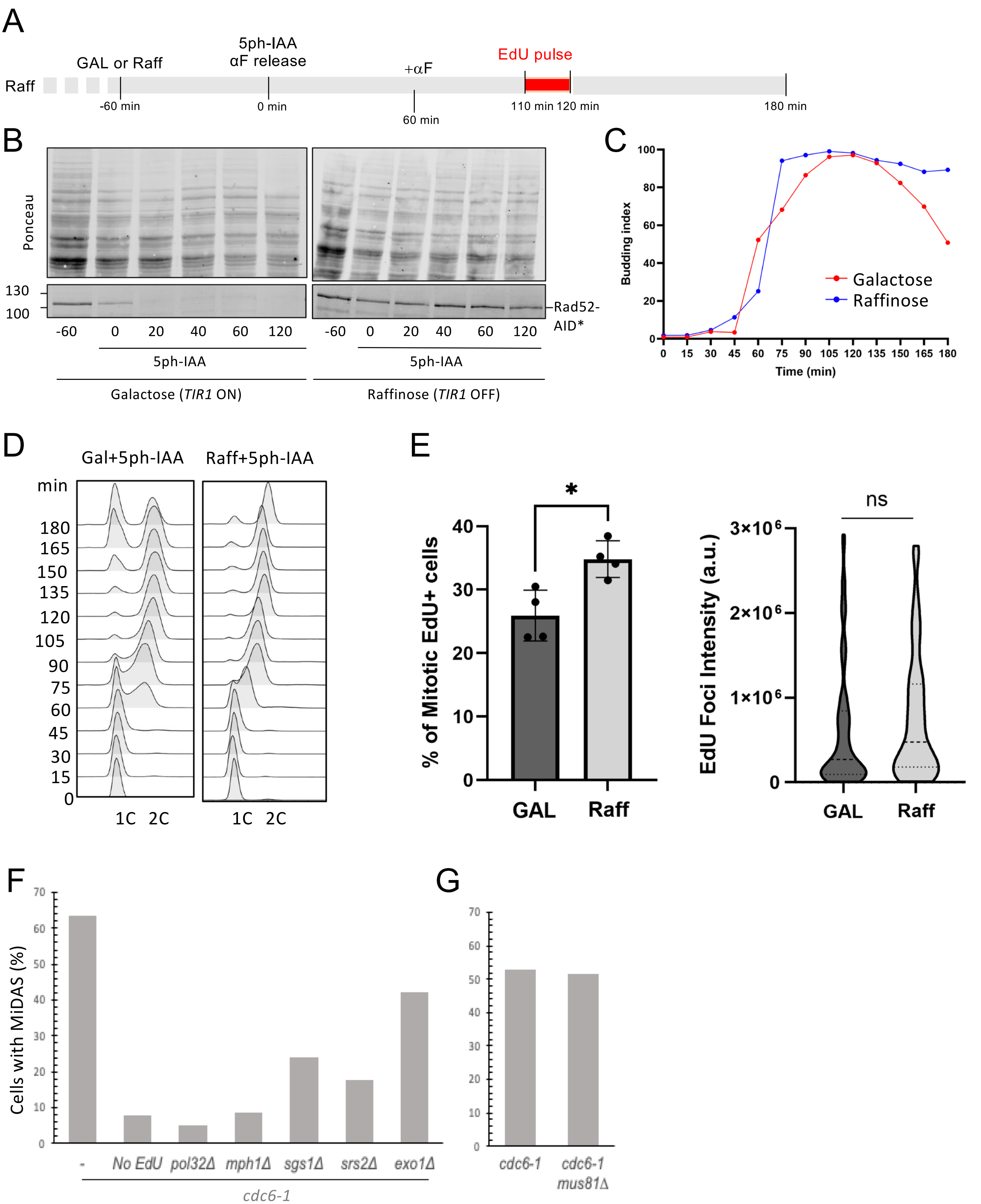
A
Raff
GAL or Raff
5ph-IAA
αF release
+αF
EdU pulse
-60 min
0 min
60 min
110 min 120 min
180 min
B
Ponceau
130
100
Rad52-AID*
-60 0 20 40 60 120
5ph-IAA
Galactose (TIR1 ON)
Raffinose (TIR1 OFF)
C
Budding index
Time (min)
Galactose
Raffinose
D
Gal+5ph-IAA
Raff+5ph-IAA
min
1C 2C
E
% of Mitotic EdU+ cells
EdU Foci Intensity (a.u.)
ns
GAL
Raff
F
Cells with MiDAS (%)
-
No EdU
pol32Δ
mph1Δ
sgs1Δ
srs2Δ
exo1Δ
cdc6-1
G
cdc6-1
cdc6-1 mus81Δ

Figure EV4. MiDAS in *cdc6-1* cells is partly dependent on Rad52 and independent of *MUS81* (related to Fig. 3).

(**A**) *cdc6-1, RAD52-AID2*, and *GAL-OsTIR1* cells were depleted of Rad52, or not, by auxin addition in the presence (Gal) or absence (Raff) of OsTIR1 before release from G_1 arrest. Cells were pulsed for 10 min with EdU at 110 min to detect MiDAS, and α-Factor was added back to prevent cells from entering a subsequent S phase. (**B**) Western blot analysis of Rad52-AID depletion. (**C**, **D**) Budding index and FACS data showing that *cdc6-1* cells exit mitosis precociously in the absence of Rad52 (Gal). (**E**) Percent cells undergoing MiDAS (left, $n = 4$ mean ± SD, >100 cells in each replicate, *p value = 0.02857, statistical analysis by Mann–Whitney test) in the absence (Gal) or presence (Raff) of Rad52, along with EdU foci intensities (right, at least 160 cells were counted, mean ± SD, n.s. p value = 0.9986, statistical analysis by Mann–Whitney test). (**F**) QIBC scoring of MiDAS in *cdc6-1* cells deleted for the indicated genes. (**G**) MiDAS scored by fluorescence microscopy in *cdc6-1* cells lacking or not *MUS81*.

 © The Author(s)

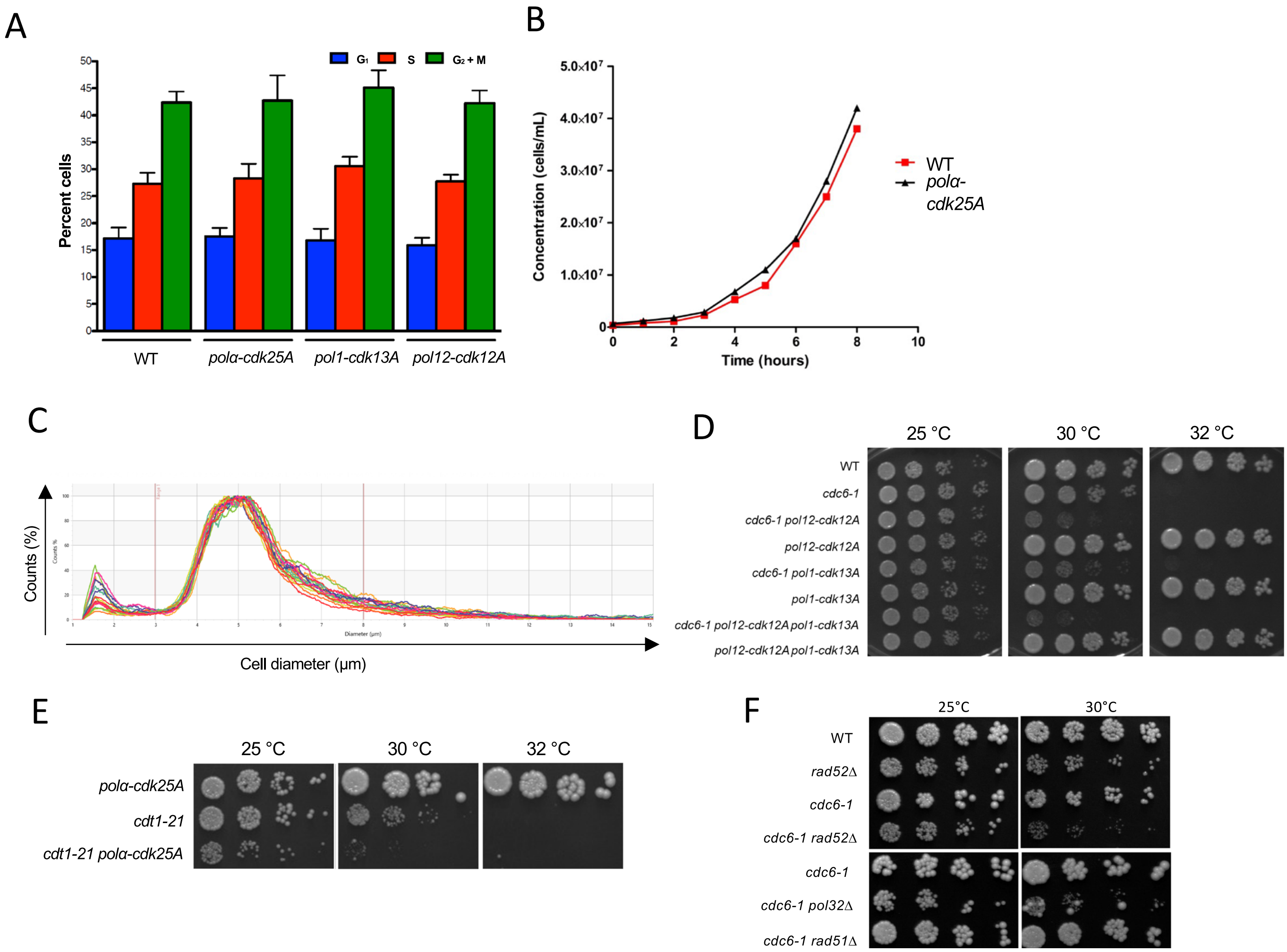

Figure EV5. Polα-cdk25A is proficient for canonical DNA replication, but lethal in hypo-licensed cells (related to Fig. 4).

(**A**) Cell cycle phases duration of WT, *pol1-cdk13A*, *pol12-cdk12A*, and *polα-cdk25A* mutants calculated from EdU-PI bivariate FACS and cell doubling time (mean ± SEM, $n = 3$ biological replicates). (**B**) Growth curve of WT and polα-cdk25A mutant. (**C**) Cell size distribution of WT and polα-cdk25A cells. (**D**) WT, *pol1-cdk13A*, *pol12-cdk12A*, and *polα-cdk25A* cells containing or not *cdc6-1* were spotted in fivefold serial dilutions on rich medium and grown at the indicated temperatures. (**E**) Synthetic lethality of *polα-cdk25A* and *cdt1-21* mutations. Cells were spotted in fivefold serial dilutions and grown for 2 days at the indicated temperatures. (**F**) *cdc6-1* shows co-lethality with *rad52Δ* and *pol32Δ*, but not with *rad51Δ*.

© The Author(s)

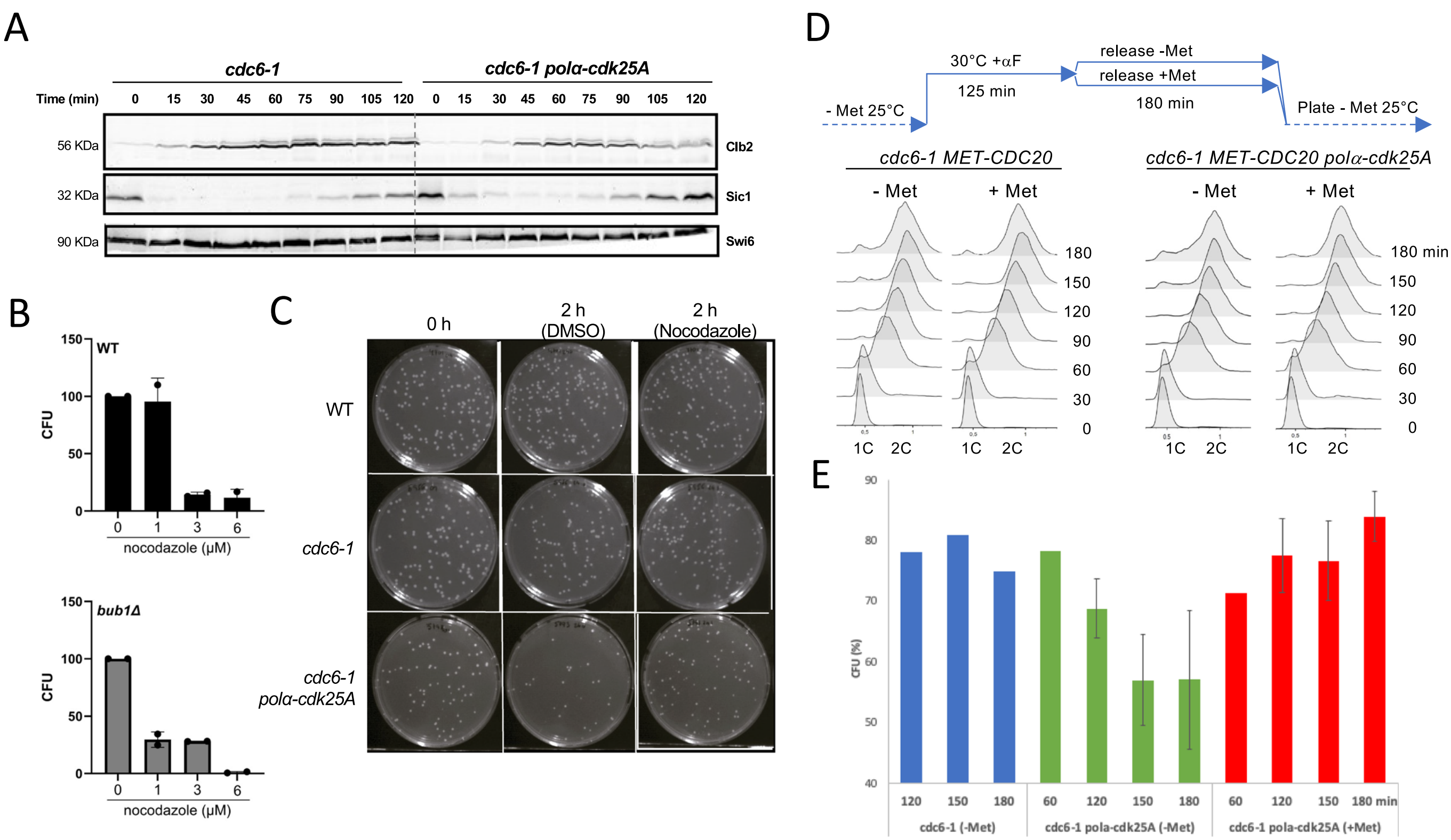

Figure EV6. Polα phosphorylation is required for prolonged metaphase arrest in *cdc6-1* cells (related to Fig. 5).

(**A**) Western blot analysis monitoring Sic1 and Clb2 levels in *cdc6-1* and *cdc6-1 polα-cdk25A* cells after release from G_1 at 30 °C (Swi6, loading control). (**B**) Low dose nocodazole (1 μM) slows down mitosis without affecting viability of WT cells. Colony formation assay of WT and *bub1Δ* cells after 4 h incubation with nocodazole at the indicated concentrations. CFU values are normalized to timepoint 0 h set at CFU = 100 (mean ± SEM, $n = 3$). (**C**) Colony-forming assay for WT, *cdc6-1* and *cdc6-1 polα-cdk25A* cells grown at 30 °C for 0 or 2 h in presence of DMSO (vehicle) or nocodazole before plating. (**D**) *cdc6-1 MET-CDC20* and *cdc6-1 MET-CDC20 polα-cdk25A* cells were synchronized in G_1 at 30 °C and released in the presence (-Met) or absence (+Met) of *CDC20* synthesis. Cell cycle progression by cytometry of DNA content. At different times after release, cells were plated on –Met 25 °C to score for viability. (**E**) Colony-forming units (CFU) for cells spotted at different times after G_1 release +/−Met. The drop in viability seen after 120 min in the *cdc6-1 polα-cdk25A* cells (−Met) is suppressed by transient metaphase arrest induced by Cdc20 depletion (+Met). Mean ± SD for 120, 150, and 180 min timepoints ($n = 3$, technical triplicate; 2 biological duplicates).

 © The Author(s)
