# Supplementary material for: Switch from canonical to recombination-dependent DNA synthesis by M-CDK phosphorylation of DNA Polα
Source: EMBO J. 2026 Oct 10;45(21):7621–46. doi: 10.1038/s44318-026-00919-5 (PMC13728163; doi:10.1038/s44318-026-00919-5)
Supplement: Supplementary file 2 [file 44318_2026_919_MOESM1_ESM.pdf]

# Appendix

to

“**Switch from canonical to recombination-dependent DNA synthesis by M-CDK phosphorylation of DNA Polα**“

by JD Barba Tena et al.


Page 2: Appendix Figure S1: Genome-wide chromosome replication profiles generated by BrdU-nanopore sequencing of WT and *cdc6-1* cells at 25°C.

Page 3: Appendix Figure S2: Genome-wide chromosome replication profiles generated by BrdU-nanopore sequencing of WT and *cdc6-1* cells at 30°C.

Page 4: Appendix Figure S3: Genome-wide MiDAS profiles created by BrdU-nanopore sequencing of WT and *cdc6-1* cells at 30°C.

Page 5: Appendix Figure S4: *POL1* sequence with *cdk13A* mutations and diagnostic restriction sites.

Page 12: Appendix Figure S5: *POL12* sequence with *cdk12A* mutations and diagnostic restriction sites.

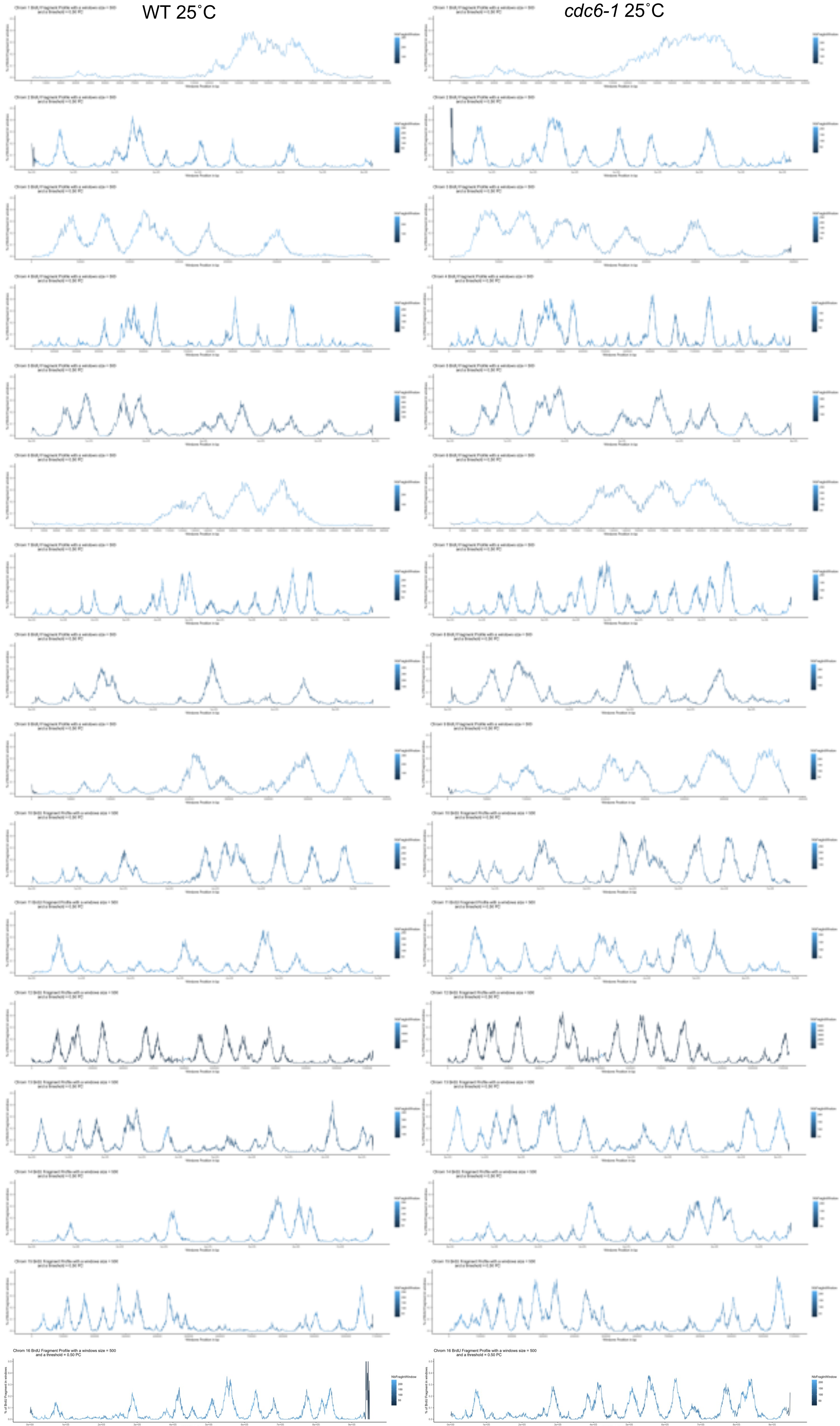


Appendix Figure S1: Genome-wide chromosome replication profiles generated by BrdU-nanopore sequencing of WT and *cdc6-1* cells at 25°C.

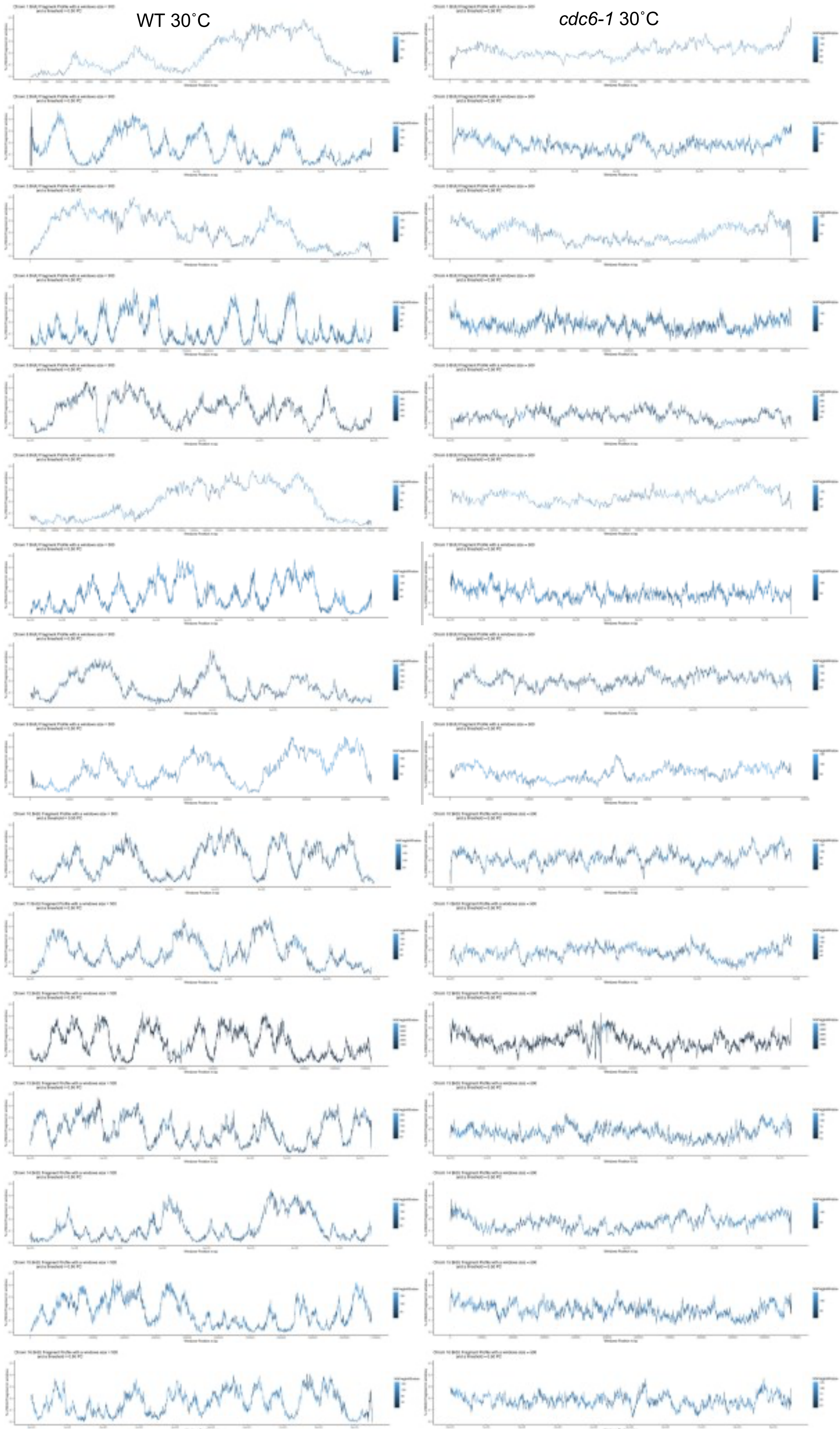


Appendix Figure S2: Genome-wide chromosome replication profiles generated by BrdU-nanopore sequencing of WT and *cdc6-1* cells at 30°C.

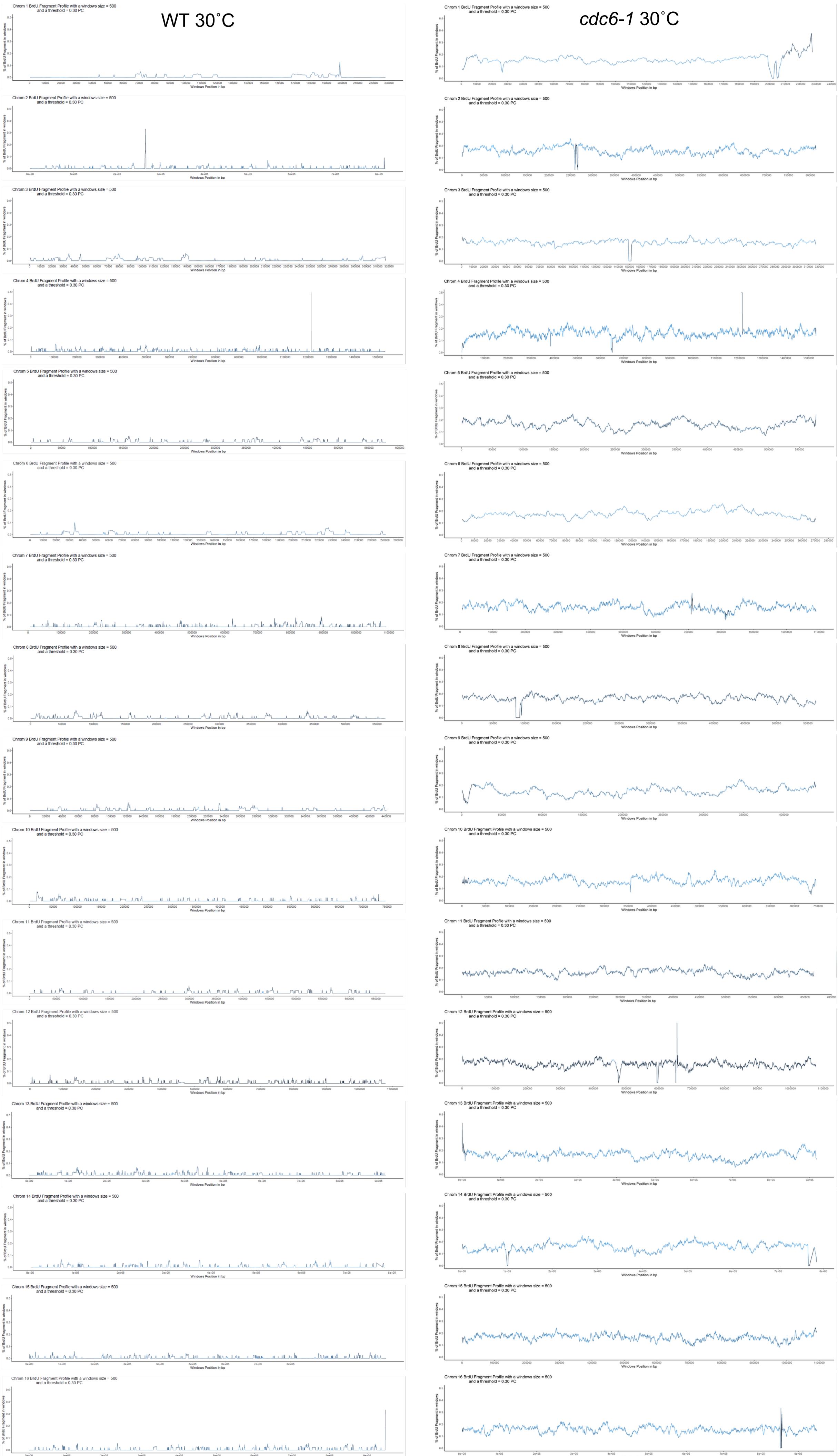


Appendix Figure S3: Genome-wide MiDAS profiles created by BrdU-nanopore sequencing of WT and *cdc6-1* cells at 30°C.

```
Alignment of Sequence_1: [POL1] with Sequence_2: [pol1-13A sequenced]

Similarity: 4358/4407 (98.89 %)

              M  S  S  K  S  E  K  L  E  K  L  R  K  L  Q  A  A  R  N  G
Seq_1  1      ATGTCTTCTAAAAGTGAAAAATTAGAAAAATTGAGAAAGCTGCAAGCCGCTCGAAATGGT  60
              ||||||||||||||||||||||||||||||||||||||||||||||||||||||||||||
Seq_2  1      ATGTCTTCTAAAAGTGAAAAATTAGAAAAATTGAGAAAGCTGCAAGCCGCTCGAAATGGT  60
              M  S  S  K  S  E  K  L  E  K  L  R  K  L  Q  A  A  R  N  G

              T  S  I  D  D  Y  E  G  D  E  S  D  G  D  R  I  Y  D  E  I
Seq_1  61     ACATCCATCGATGATTACGAAGGTGACGAATCAGATGGTGACAGAATATACGACGAAATC  120
              ||||||||||||||||||||||||||||||||||||||||||||||||||||||||||||
Seq_2  61     ACATCCATCGATGATTACGAAGGTGACGAATCAGATGGTGACAGAATATACGACGAAATC  120
              T  S  I  D  D  Y  E  G  D  E  S  D  G  D  R  I  Y  D  E  I

              D  E  K  E  Y  R  A  R  K  R  Q  E  L  L  H  D  D  F  V  V
Seq_1  121    GACGAAAAAGAATACAGGGCCAGAAAGCGCCAGGAATTGCTGCATGACGATTTTGTTGTA  180
              ||||||||||||||||||||||||||||||||||||||||||||||||||||||||||||
Seq_2  121    GACGAAAAAGAATACAGGGCCAGAAAGCGCCAGGAATTGCTGCATGACGATTTTGTTGTA  180
              D  E  K  E  Y  R  A  R  K  R  Q  E  L  L  H  D  D  F  V  V

              D  D  D  G  V  G  Y  V  D  R  G  V  E  E  D  W  R  E  V  D
Seq_1  181    GATGACGATGGTGTAGGCTACGTCGATCGTGGTGTCGAAGAAGATTGGAGGGAAGTTGAT  240
              ||||||||||||||||||||||||||||||||||||||||||||||||||||||||||||
Seq_2  181    GATGACGATGGTGTAGGCTACGTCGATCGTGGTGTCGAAGAAGATTGGAGGGAAGTTGAT  240
              D  D  D  G  V  G  Y  V  D  R  G  V  E  E  D  W  R  E  V  D

              N  S  S  S  D  E  D  T  G  N  L  A  S  K  D  S  K  R  K  K
Seq_1  241    AACAGTTCAAGTGATGAAGACACTGGAAACCTTGCTAGCAAGGATTCTAAAAGGAAGAAG  300
              ||||||||||||||||||||||||||||||||||||||||||||||||||||||||||||
Seq_2  241    AACAGTTCAAGTGATGAAGACACTGGAAACCTTGCTAGCAAGGATTCTAAAAGGAAGAAG  300
              N  S  S  S  D  E  D  T  G  N  L  A  S  K  D  S  K  R  K  K

              N  I  K  R  E  K  D  H  Q  I  T  D  M  L  R  T  Q  H  S  K
Seq_1  301    AATATAAAAAGGGAGAAAGACCACCAGATTACAGATATGCTGCGAACTCAACATTCCAAA  360
              ||||||||||||||||||||||||||||||||||||||||||||||||||||||||||||
Seq_2  301    AATATAAAAAGGGAGAAAGACCACCAGATTACAGATATGCTGCGAACTCAACATTCCAAA  360
              N  I  K  R  E  K  D  H  Q  I  T  D  M  L  R  T  Q  H  S  K

              S  T  L  L  A  H  A  K  K  S  Q  K  K  S  I  P  I  D  N  F
Seq_1  361    TCAACACTGCTAGCACATGCAAAAAAAATCCCAAAAAAAAAGTATCCCCATCGATAATTTT  420
              ||||||||||||||||||||||||||||||||||||||||||||||||||||||||||||
Seq_2  361    TCAACACTGCTAGCACATGCAAAAAAAATCCCAAAAAAAAAGTATCCCCATCGATAATTTT  420
              S  T  L  L  A  H  A  K  K  S  Q  K  K  S  I  P  I  D  N  F

              D  D  I  L  G  E  F  E  S  G  E  V  E  K  P  N  I  L  L  P
Seq_1  421    GATGACATTCTTGGTGAGTTTGAATCTGGTGAAGTAGAAAAACCCAATATTTTACTGCCA  480
              ||||||||||||||||||||||||||||||||||||||||||||||||||||||||||||
Seq_2  421    GATGACATTCTTGGTGAGTTTGAATCTGGTGAAGTAGAAAAACCCAATATTTTACTGCCA  480
              D  D  I  L  G  E  F  E  S  G  E  V  E  K  P  N  I  L  L  P

                                                           ApoI
              S  K  L  R  E  N  L  N  S  S  P  T  S  E  F  K  S  S  I  K
Seq_1  481    TCCAAATTAAGGGAAAATTTAAATTCTTCGCCGACTAGTGAATTTAAATCGTCAATAAAG  540
              ||||||||||||||||||||||||||  | |||||||| |||||||| ||||||||||||
Seq_2  481    TCCAAATTAAGGGAAAATTTAAATTCGGCACCGACTAGCGAATTTAAGTCGTCAATAAAG  540
              S  K  L  R  E  N  L  N  S  A  P  T  S  E  F  K  S  S  I  K

              R  V  N  G  N  D  E  S  S  H  D  A  G  I  S  K  K  V  K  I
Seq_1  541    AGAGTTAACGGAAACGATGAGTCAAGTCATGATGCCGGTATTTCTAAAAAAGTCAAGATT  600
              ||||||||||||||||||||||||||||||||||||||||||||||||||||||||||||
Seq_2  541    AGAGTTAACGGAAACGATGAGTCAAGTCATGATGCCGGTATTTCTAAAAAAGTCAAGATT  600
              R  V  N  G  N  D  E  S  S  H  D  A  G  I  S  K  K  V  K  I
```

```
              D  P  D  S  S  T  D  K  Y  L  E  I  E   S  S  P  L  K  L  Q
Seq_1   601   GATCCAGATTCAAGTACTGATAAATATTTAGAAATCGAA--TCATCACCGTTAAAATTAC   658
              |||||||||||||||||||||||||||||||||||||||  |   |||||||||||||||
Seq_2   601   GATCCAGATTCAAGTACTGATAAATATTTAGAAATCGAAAGTG--CACCGTTAAAATTAC   658
              D  P  D  S  S  T  D  K  Y  L  E  I  E  S  A     P  L  K  L  Q
                                                        ApaLI

               S  R  K  L  R  Y  A  N  D  V  Q  D  L  L  D  D  V  E  N  S
Seq_1   659   AAAGCAGGAAACTGCGCTACGCAAATGATGTACAGGATTTATTGGATGATGTGGAAAATT   718
              |||||||||||||||||||||||||||||||||| ||| ||||||||||||||||||||
Seq_2   659   AAAGCAGGAAACTGCGCTACGCAAATGATGTACAAGATCTATTGGATGATGTGGAAAATG   718
               S  R  K  L  R  Y  A  N  D  V  Q  D  L  L  D  D  V  E  N  A
                                                 BglII

               P  V  V  A  T  K  R  Q  N  V  L  Q  D  T  L  L  A  N  P  P
Seq_1   719   CCCCAGTAGTAGCCACAAAGAGACAGAATGTACTTCAAGATACGTTACTAGCAAATCCAC   778
              |||| |||||||||||||||||||||||||||||||||||||||||||||||||||||||
Seq_2   719   CCCCGGTAGTAGCCACAAAGAGACAGAATGTACTTCAAGATACGTTACTAGCAAATCCAC   778
               P  V  V  A  T  K  R  Q  N  V  L  Q  D  T  L  L  A  N  P  P

               S  A  Q  S  L  A  D  E  E  D  D  E  D  S  D  E  D  I  I  L
Seq_1   779   CATCCGCTCAAAGTTTGGCTGATGAAGAAGATGATGAAGATAGTGATGAAGATATTATTC   838
              ||||||||||||||||||||||||||||||||||||||||||||||||||||||||||||
Seq_2   779   CATCCGCTCAAAGTTTGGCTGATGAAGAAGATGATGAAGATAGTGATGAAGATATTATTC   838
               S  A  Q  S  L  A  D  E  E  D  D  E  D  S  D  E  D  I  I  L

               K  R  R  T  M  R   S  V  T  T  T  R  R  V  N  I  D  S  R
Seq_1   839   TGAAAAGAAGAACTATGAGA-AGTGTGACAACAACTAGACGCGTGAATATAGACTCAAGA   897
              ||||||||||||||||||||    || || ||||||||||||||||||||||||||||||
Seq_2   839   TGAAAAGAAGAACTATGAGATC-GGTTACCACAACTAGACGCGTGAATATAGACTCAAGA   897
               K  R  R  T  M  R  S   V  T  T  T  R  R  V  N  I  D  S  R

              S  N  P  S  T  S  P  F  V  T  A  P  G  T  P  I  G  I  K  G
Seq_1   898   AGTAACCCTTCAACTTCACCGTTTGTCACAGCACCCGGAACGCCAATCGGTATTAAAGGA   957
              ||||||||||||||  ||||||||| ||||||||||||| | |||||||||||||||||
Seq_2   898   AGTAACCCTTCAACGGCACCGTTTGGCACAGCACCCGGAGCTCCAATCGGTATTAAAGGG   957
              S  N  P  S  T  A  P  F  G  T  A  P  G  A  P  I  G  I  K  G
                                                   SacI

              L  T  P  S  K  S  L  Q  S  N  T  D  V  A  T  L  A  V  N  V
Seq_1   958   CTAACTCCAAGTAAATCTTTGCAGAGCAACACCGATGTTGCTACACTTGCGGTTAATGTC   1017
              ||| ||||||||||||||||||||||||||||||||||||||||||||||||||||||||
Seq_2   958   CTAGCTCCAAGTAAATCTTTGCAGAGCAACACCGATGTTGCTACACTTGCGGTTAATGTC   1017
              L  A  P  S  K  S  L  Q  S  N  T  D  V  A  T  L  A  V  N  V
              NheI

              K  K  E  D  V  V  D  P  E  T  D  T  F  Q  M  F  W  L  D  Y
Seq_1   1018  AAAAAGGAAGATGTGGTAGATCCGGAAACTGATACTTTCCAAATGTTTTGGTTGGATTAC   1077
              ||||||||||||||||||||||||||||||||||||||||||||||||||||||||||||
Seq_2   1018  AAAAAGGAAGATGTGGTAGATCCGGAAACTGATACTTTCCAAATGTTTTGGTTGGATTAC   1077
              K  K  E  D  V  V  D  P  E  T  D  T  F  Q  M  F  W  L  D  Y

              C  E  V  N  N  T  L  I  L  F  G  K  V  K  L  K  D  D  N  C
Seq_1   1078  TGTGAAGTGAACAACACTTTGATTTTATTTGGTAAGGTTAAACTAAAGGATGATAATTGT   1137
              ||||||||||||||||||||||||||||||||||||||||||||||||||||||||||||
Seq_2   1078  TGTGAAGTGAACAACACTTTGATTTTATTTGGTAAGGTTAAACTAAAGGATGATAATTGT   1137
              C  E  V  N  N  T  L  I  L  F  G  K  V  K  L  K  D  D  N  C

              V  S  A  M  V  Q  I  N  G  L  C  R  E  L  F  F  L  P  R  E
Seq_1   1138  GTGTCAGCCATGGTTCAAATCAACGGTCTCTGTAGAGAGCTATTTTTCCTTCCAAGAGAG   1197
              ||||||||||||||||||||||||||||||||||||||||||||||||||||||||||||
Seq_2   1138  GTGTCAGCCATGGTTCAAATCAACGGTCTCTGTAGAGAGCTATTTTTCCTTCCAAGAGAG   1197
              V  S  A  M  V  Q  I  N  G  L  C  R  E  L  F  F  L  P  R  E

              G  K  T  P  T  D  I  H  E  E  I  I  P  L  L  M  D  K  Y  G
Seq_1   1198  GGTAAAACACCTACCGATATACACGAGGAAATAATTCCGTTACTGATGGATAAATATGGA   1257
              |||||  | |||||||||||||||||||||||||||||||||||||||||||||||||||
Seq_2   1198  GGTAAGGCCCCTACCGATATACACGAGGAAATAATTCCGTTACTGATGGATAAATATGGA   1257
              G  K  A  P  T  D  I  H  E  E  I  I  P  L  L  M  D  K  Y  G
```

```
             L  D  N  I  R  A  K  P  Q  K  M  K  Y  S  F  E  L  P  D  I
Seq_1  1258  CTGGATAATATCCGCGCTAAACCTCAAAAAATGAAATATTCGTTTGAGCTTCCTGACATT  1317
             |||||||||||||||||||| |||||||||||||||||||||||||||||||||||||||
Seq_2  1258  CTGGATAATATCCGCGCTAAGCCTCAAAAAATGAAATATTCGTTTGAGCTTCCTGACATT  1317
             L  D  N  I  R  A  K  P  Q  K  M  K  Y  S  F  E  L  P  D  I

             P  S  E  S  D  Y  L  K  V  L  L  P  Y  Q  T  P  K  S  S  R
Seq_1  1318  CCATCTGAAAGTGATTATTTGAAAGTCTTACTTCCATACCAAACACCGAAGTCCAGTCGA  1377
             |||||||||||||||||||||||||||||||||||||||||  | |||||||||||||||
Seq_2  1318  CCATCTGAAAGTGATTATTTGAAAGTCTTACTTCCATACCAGGCTCCGAAGTCCAGTCGA  1377
             P  S  E  S  D  Y  L  K  V  L  L  P  Y  Q  A  P  K  S  S  R

             D  T  I  P  S  D  L  S  S  D  T  F  Y  H  V  F  G  G  N  S
Seq_1  1378  GACACCATTCCATCGGATTTATCTAGCGATACCTTTTATCATGTTTTTGGTGGGAATTCC  1437
             ||||||||||||||| ||||||||||||||||||||||||||||||||||||||||||||
Seq_2  1378  GACACCATTCCATCGAATTTATCTAGCGATACCTTTTATCATGTTTTTGGTGGGAATTCC  1437
             D  T  I  P  S  N  L  S  S  D  T  F  Y  H  V  F  G  G  N  S

             N  I  F  E  S  F  V  I  Q  N  R  I  M  G  P  C  W  L  D  I
Seq_1  1438  AATATTTTTGAAAGTTTTGTTATTCAAAATAGGATTATGGGACCATGCTGGTTAGATATA  1497
             ||||||||||||||||||||||||||||||||||||||||||||||||||||||||||||
Seq_2  1438  AATATTTTTGAAAGTTTTGTTATTCAAAATAGGATTATGGGACCATGCTGGTTAGATATA  1497
             N  I  F  E  S  F  V  I  Q  N  R  I  M  G  P  C  W  L  D  I

             K  G  A  D  F  N  S  I  R  N  A  S  H  C  A  V  E  V  S  V
Seq_1  1498  AAGGGAGCAGATTTCAATTCAATTCGTAATGCTTCTCACTGTGCGGTTGAAGTTTCGGTA  1557
             ||||||||||||||||||||||| ||||||||||||||||||||||||||||||||||||
Seq_2  1498  AAGGGAGCAGATTTCAATTCAATACGTAATGCTTCTCACTGTGCGGTTGAAGTTTCGGTA  1557
             K  G  A  D  F  N  S  I  R  N  A  S  H  C  A  V  E  V  S  V
                                   SnaBI

             D  K  P  Q  N  I  T  P  T  T  T  K  T  M  P  N  L  R  C  L
Seq_1  1558  GATAAGCCTCAGAATATCACGCCTACTACAACAAAGACAATGCCTAATCTGAGATGTCTA  1617
             |||||||||||||||||| |||||||||||||||||||||||||||||||||||||||||
Seq_2  1558  GATAAGCCTCAGAATATCGCGCCTACTACAACAAAGACAATGCCTAATCTGAGATGTCTA  1617
             D  K  P  Q  N  I  A  P  T  T  T  K  T  M  P  N  L  R  C  L

             S  L  S  I  Q  T  L  M  N  P  K  E  N  K  Q  E  I  V  S  I
Seq_1  1618  TCTTTATCGATTCAGACGTTAATGAATCCCAAGGAAAATAAGCAAGAGATTGTTTCGATA  1677
             ||||||||||||||||||||||||||||||||||||||||||||||||||||||||||||
Seq_2  1618  TCTTTATCGATTCAGACGTTAATGAATCCCAAGGAAAATAAGCAAGAGATTGTTTCGATA  1677
             S  L  S  I  Q  T  L  M  N  P  K  E  N  K  Q  E  I  V  S  I

             T  L  S  A  Y  R  N  I  S  L  D  S    P  I  P  E  N  I  K  P
Seq_1  1678  ACGTTATCAGCATATAGAAATATCTCGTTGGATTCA--CCGATACCTGAAAATATAAAAC  1735
             || |||   |||||||||||||||||||||||  |   ||||||||||||||||||||||
Seq_2  1678  ACCTTAAGTGCATATAGAAATATCTCGTTGGA--CGCCCCGATACCTGAAAATATAAAAC  1735
             T  L  S  A  Y  R  N  I  S  L  D    A  P  I  P  E  N  I  K  P
               AflII

               D  D  L  C  T  L  V  R  P  P  Q  S  T  S  F  P  L  G  L  A
Seq_1  1736  CAGACGACTTGTGTACTTTAGTAAGGCCGCCTCAGTCAACTAGTTTCCCATTAGGTTTAG  1795
             ||||||||||||||||||||||||||||||||||||||||||||||||||||||||||||
Seq_2  1736  CAGACGACTTGTGTACTTTAGTAAGGCCGCCTCAGTCAACTAGTTTCCCATTAGGTTTAG  1795
               D  D  L  C  T  L  V  R  P  P  Q  S  T  S  F  P  L  G  L  A

               A  L  A  K  Q  K  L  P  G  R  V  R  L  F  N  N  E  K  A  M
Seq_1  1796  CTGCACTGGCAAAACAGAAACTACCCGGTAGAGTCAGGTTGTTCAACAACGAAAAGGCCA  1855
             ||||||||||||||||||||||||||||||||||||||||||||||||||||||||||||
Seq_2  1796  CTGCACTGGCAAAACAGAAACTACCCGGTAGAGTCAGGTTGTTCAACAACGAAAAGGCCA  1855
               A  L  A  K  Q  K  L  P  G  R  V  R  L  F  N  N  E  K  A  M
```

```
                L  S  C  F  C  A  M  L  K  V  E  D  P  D  V  I  I  G  H  R
Seq_1  1856  TGCTATCTTGTTTTTGTGCTATGTTAAAAGTGGAAGACCCAGATGTTATCATAGGCCATC  1915
             ||||||||||||||||||||||||||||||||||||||||||||||||||||||||||||
Seq_2  1856  TGCTATCTTGTTTTTGTGCTATGTTAAAAGTGGAAGACCCAGATGTTATCATAGGCCATC  1915
                L  S  C  F  C  A  M  L  K  V  E  D  P  D  V  I  I  G  H  R

                L  Q  N  V  Y  L  D  V  L  A  H  R  M  H  D  L  N  I  P  T
Seq_1  1916  GTTTGCAAAATGTCTACTTAGATGTTTTAGCTCATAGAATGCATGATCTCAACATTCCAA  1975
             ||||||||||||||||||||||||||||||||||||||||||||||||||||||||||||
Seq_2  1916  GTTTGCAAAATGTCTACTTAGATGTTTTAGCTCATAGAATGCATGATCTCAACATTCCAA  1975
                L  Q  N  V  Y  L  D  V  L  A  H  R  M  H  D  L  N  I  P  T

                F  S  S  I  G  R  R  L  R  R  T  W  P  E  K  F  G  R  G  N
Seq_1  1976  CGTTCAGCTCTATTGGCCGTCGTTTAAGAAGGACTTGGCCTGAAAAATTCGGTAGAGGAA  2035
             ||||||||||||||||||||||||||||||||||||||||||||||||||||||||||||
Seq_2  1976  CGTTCAGCTCTATTGGCCGTCGTTTAAGAAGGACTTGGCCTGAAAAATTCGGTAGAGGAA  2035
                F  S  S  I  G  R  R  L  R  R  T  W  P  E  K  F  G  R  G  N

                S  N  M  N  H  F  F  I  S  D  I  C  S  G  R  L  I  C  D  I
Seq_1  2036  ATTCGAATATGAACCATTTTTTTATTAGTGACATTTGCTCTGGTAGGCTGATATGTGATA  2095
             ||||||||||||||||||||||||||||||||||||||||||||||||||||||||||||
Seq_2  2036  ATTCGAATATGAACCATTTTTTTATTAGTGACATTTGCTCTGGTAGGCTGATATGTGATA  2095
                S  N  M  N  H  F  F  I  S  D  I  C  S  G  R  L  I  C  D  I

                A  N  E  M  G  Q  S  L  T  P  K  C  Q  S  W  D  L  S  E  M
Seq_1  2096  TCGCCAATGAAATGGGTCAATCATTAACACCCAAATGTCAAAGTTGGGATCTTTCAGAGA  2155
             |||||||||||||||||||||| ||   | ||||||||||||||||||||||||||||||
Seq_2  2096  TCGCCAATGAAATGGGTCAATCCTTGGCTCCCAAATGTCAAAGTTGGGATCTTTCAGAGA  2155
                A  N  E  M  G  Q  S  L  A  P  K  C  Q  S  W  D  L  S  E  M

                Y  Q  V  T  C  E  K  E  H  K  P  L  D  I  D  Y  Q  N  P  Q
Seq_1  2156  TGTATCAAGTTACATGTGAAAAGGAGCATAAGCCGTTAGATATTGATTATCAAAATCCAC  2215
             ||||||||||||||||||||||||||||||||||||||||||||||||||||||||||||
Seq_2  2156  TGTATCAAGTTACATGTGAAAAGGAGCATAAGCCGTTAGATATTGATTATCAAAATCCAC  2215
                Y  Q  V  T  C  E  K  E  H  K  P  L  D  I  D  Y  Q  N  P  Q

                Y  Q  N  D  V  N  S  M  T  M  A  L  Q  E  N  I  T  N  C  M
Seq_1  2216  AATACCAAAATGATGTAAATAGTATGACAATGGCCCTACAAGAGAATATTACTAATTGTA  2275
             ||||||||||||||||||||||||||||||||||||||||||||||||||||||||||||
Seq_2  2216  AATACCAAAATGATGTAAATAGTATGACAATGGCCCTACAAGAGAATATTACTAATTGTA  2275
                Y  Q  N  D  V  N  S  M  T  M  A  L  Q  E  N  I  T  N  C  M

                I  S  A  E  V  S  Y  R  I  Q  L  L  T  L  T  K  Q  L  T  N
Seq_1  2276  TGATTTCTGCTGAGGTGTCTTATAGAATTCAATTATTAACCTTGACTAAACAGTTGACAA  2335
             ||||||||||||||||||||||||||||||||||||||||||||||||||||||||||||
Seq_2  2276  TGATTTCTGCTGAGGTGTCTTATAGAATTCAATTATTAACCTTGACTAAACAGTTGACAA  2335
                I  S  A  E  V  S  Y  R  I  Q  L  L  T  L  T  K  Q  L  T  N

                L  A  G  N  A  W  A  Q  T  L  G  G  T  R  A  G  R  N  E  Y
Seq_1  2336  ATTTGGCTGGTAATGCATGGGCCCAAACGCTAGGCGGTACAAGAGCTGGTAGAAATGAGT  2395
             ||||||||||||||||||||||||||||||||||||||||||||||||||||||||||||
Seq_2  2336  ATTTGGCTGGTAATGCATGGGCCCAAACGCTAGGCGGTACAAGAGCTGGTAGAAATGAGT  2395
                L  A  G  N  A  W  A  Q  T  L  G  G  T  R  A  G  R  N  E  Y

                I  L  L  H  E  F  S  R  N  G  F  I  V  P  D  K  E  G  N  R
Seq_1  2396  ATATCTTACTACATGAATTTTCAAGAAATGGTTTTATTGTTCCTGACAAAGAAGGCAATA  2455
             ||||||||||||||||||||||||||||||||||||||||||||||||||||||||||||
Seq_2  2396  ATATCTTACTACATGAATTTTCAAGAAATGGTTTTATTGTTCCTGACAAAGAAGGCAATA  2455
                I  L  L  H  E  F  S  R  N  G  F  I  V  P  D  K  E  G  N  R

                S  R  A  Q  K  Q  R  Q  N  E  E  N  A  D  A  P  V  N  S  K
Seq_1  2456  GAAGTAGAGCTCAGAAACAAAGACAAAATGAAGAAAATGCTGATGCACCAGTTAATTCTA  2515
             ||||||||||||||||||||||||||||||||||||||||||||||||||||||||||||
Seq_2  2456  GAAGTAGAGCTCAGAAACAAAGACAAAATGAAGAAAATGCTGATGCACCAGTTAATTCTA  2515
                S  R  A  Q  K  Q  R  Q  N  E  E  N  A  D  A  P  V  N  S  K
```

```
               K  A  K  Y  Q  G  G  L  V  F  E  P  E  K  G  L  H  K  N  Y
Seq_1   2516  AAAAGGCAAAATATCAGGGTGGTTTAGTTTTTGAACCTGAGAAAGGTCTTCATAAGAATT  2575
              ||||||||||||||||||||||||||||||||||||||||||||||||||||||||||||
Seq_2   2516  AAAAGGCAAAATATCAGGGTGGTTTAGTTTTTGAACCTGAGAAAGGTCTTCATAAGAATT  2575
               K  A  K  Y  Q  G  G  L  V  F  E  P  E  K  G  L  H  K  N  Y

               V  L  V  M  D  F  N  S  L  Y  P  S  I  I  Q  E  F  N  I  C
Seq_1   2576  ACGTTTTAGTCATGGACTTTAATTCTTTGTATCCATCTATTATCCAGGAATTTAATATAT  2635
              ||||||||||||||||||||||||||||||||||||||||||||||||||||||||||||
Seq_2   2576  ACGTTTTAGTCATGGACTTTAATTCTTTGTATCCATCTATTATCCAGGAATTTAATATAT  2635
               V  L  V  M  D  F  N  S  L  Y  P  S  I  I  Q  E  F  N  I  C

               F  T  T  V  D  R  N  K  E  D  I  D  E  L  P  S  V  P  P  S
Seq_1   2636  GTTTTACCACCGTTGATAGAAATAAGGAAGATATCGATGAGTTACCAAGCGTACCTCCTT  2695
              ||||||||||||||||||||||||||||||||||||||||||||||||||||||||||||
Seq_2   2636  GTTTTACCACCGTTGATAGAAATAAGGAAGAtatcgatgagttaccAAGCGTACCTCCTT  2695
               F  T  T  V  D  R  N  K  E  D  I  D  E  L  P  S  V  P  P  S

               E  V  D  Q  G  V  L  P  R  L  L  A  N  L  V  D  R  R  R  E
Seq_1   2696  CAGAGGTGGATCAGGGTGTTTTACCAAGGTTATTAGCTAATCTGGTGGATCGTCGACGTG  2755
              ||||||||||||||||||||||||||||||||||||||||||||||||||||||||||||
Seq_2   2696  CAGAGGTGGATCAGGGTGTTTTACCAAGGTTATTAGCTAATCTGGTGGATCGTCGACGTG  2755
               E  V  D  Q  G  V  L  P  R  L  L  A  N  L  V  D  R  R  R  E

               V  K  K  V  M  K  T  E  T  D  P  H  K  R  V  Q  C  D  I  R
Seq_1   2756  AAGTTAAGAAGGTGATGAAAACTGAAACTGATCCCCATAAGCGAGTTCAATGTGATATTC  2815
              ||||||||||||||||||||||||||||||||||||||||||||||||||||||||||||
Seq_2   2756  AAGTTAAGAAGGTGATGAAAACTGAAACTGATCCCCATAAGCGAGTTCAATGTGATATTC  2815
               V  K  K  V  M  K  T  E  T  D  P  H  K  R  V  Q  C  D  I  R

               Q  Q  A  L  K  L  T  A  N  S  M  Y  G  C  L  G  Y  V  N  S
Seq_1   2816  GTCAACAAGCTCTAAAATTGACTGCCAATTCTATGTATGGTTGTTTGGGTTATGTTAACA  2875
              ||||||||||||||||||||||||||||||||||||||||||||||||||||||||||||
Seq_2   2816  GTCAACAAGCTCTAAAATTGACTGCCAATTCTATGTATGGTTGTTTGGGTTATGTTAACA  2875
               Q  Q  A  L  K  L  T  A  N  S  M  Y  G  C  L  G  Y  V  N  S

               R  F  Y  A  K  P  L  A  M  L  V  T  N  K  G  R  E  I  L  M
Seq_1   2876  GTAGATTTTACGCAAAGCCGCTCGCCATGTTAGTCACTAATAAGGGTCGTGAGATTTTAA  2935
              ||||||||||||||||||||||||||||||||||||||||||||||||||||||||||||
Seq_2   2876  GTAGATTTTACGCAAAGCCGCTCGCCATGTTAGTCACTAATAAGGGTCGTGAGATTTTAA  2935
               R  F  Y  A  K  P  L  A  M  L  V  T  N  K  G  R  E  I  L  M

               N  T  R  Q  L  A  E  S  M  N  L  L  V  V  Y  G  D  T  D  S
Seq_1   2936  TGAATACAAGACAACTAGCTGAAAGTATGAATCTTCTTGTAGTTTATGGTGATACAGATT  2995
              ||||||||||||||||||||||||||||||||||||||||||||||||||||||||||||
Seq_2   2936  TGAATACAAGACAACTAGCTGAAAGTATGAATCTTCTTGTAGTTTATGGTGATACAGATT  2995
               N  T  R  Q  L  A  E  S  M  N  L  L  V  V  Y  G  D  T  D  S

               V  M  I  D  T  G  C  D  N  Y  A  D  A  I  K  I  G  L  G  F
Seq_1   2996  CGGTCATGATAGATACCGGTTGTGATAATTATGCGGATGCAATTAAAATTGGCTTGGGAT  3055
              ||||||||||||||||||||||||||||||||||||||||||||||||||||||||||||
Seq_2   2996  CGGTCATGATAGATACCGGTTGTGATAATTATGCGGATGCAATTAAAATTGGCTTGGGAT  3055
               V  M  I  D  T  G  C  D  N  Y  A  D  A  I  K  I  G  L  G  F

               K  R  L  V  N  E  R  Y  R  L  L  E  I  D  I  D  N  V  F  K
Seq_1   3056  TTAAAAGGCTAGTAAATGAGCGCTATAGATTATTGGAGATTGATATTGATAATGTTTTTA  3115
              ||||||||||||||||||||||||||||||||||||||||||||||||||||||||||||
Seq_2   3056  TTAAAAGGCTAGTAAATGAGCGCTATAGATTATTGGAGATTGATATTGATAATGTTTTTA  3115
               K  R  L  V  N  E  R  Y  R  L  L  E  I  D  I  D  N  V  F  K

               K  L  L  L  H  A  K  K  K  Y  A  A  L  T  V  N  L  D  K  N
Seq_1   3116  AGAAGTTACTATTACATGCCAAGAAAAAGTACGCTGCTTTGACTGTAAATTTGGACAAAA  3175
              ||||||||||||||||||||||||||||||||||||||||||||||||||||||||||||
Seq_2   3116  AGAAGTTACTATTACATGCCAAGAAAAAGTACGCTGCTTTGACTGTAAATTTGGACAAAA  3175
               K  L  L  L  H  A  K  K  K  Y  A  A  L  T  V  N  L  D  K  N
```

```
              G  N  G  T  T  V  L  E  V  K  G  L  D  M  K  R  R  E  F  C
Seq_1  3176  ATGGTAATGGAACTACTGTTCTAGAAGTTAAAGGGTTGGACATGAAGCGTCGTGAATTTT  3235
             ||||||||||||||||||||||||||||||||||||||||||||||||||||||||||||
Seq_2  3176  ATGGTAATGGAACTACTGTTCTAGAAGTTAAAGGGTTGGACATGAAGCGTCGTGAATTTT  3235
              G  N  G  T  T  V  L  E  V  K  G  L  D  M  K  R  R  E  F  C

              P  L  S  R  D  V  S  I  H  V  L  N  T  I  L  S  D  K  D  P
Seq_1  3236  GTCCACTTTCGAGGGACGTTTCTATACATGTTTTAAACACCATCCTGTCAGATAAGGACC  3295
             ||||||||||||||||||||||||||||||||||||||||||||||||||||||||||||
Seq_2  3236  GTCCACTTTCGAGGGACGTTTCTATACATGTTTTAAACACCATCCTGTCAGATAAGGACC  3295
              P  L  S  R  D  V  S  I  H  V  L  N  T  I  L  S  D  K  D  P

              E  E  A  L  Q  E  V  Y  D  Y  L  E  D  I  R  I  K  V  E  T
Seq_1  3296  CAGAAGAAGCATTGCAAGAAGTGTATGATTACTTAGAAGACATCAGAATAAAAGTGGAAA  3355
             ||||||||||||||||||||||||||||||||||||||||||||||||||||||||||||
Seq_2  3296  CAGAAGAAGCATTGCAAGAAGTGTATGATTACTTAGAAGACATCAGAATAAAAGTGGAAA  3355
              E  E  A  L  Q  E  V  Y  D  Y  L  E  D  I  R  I  K  V  E  T

              N  N  I  R  I  D  K  Y  K  I  N  M  K  L  S  K  D  P  K  A
Seq_1  3356  CCAATAACATTAGAATTGATAAATATAAGATCAATATGAAGCTTTCAAAAGATCCCAAGG  3415
             ||||||||||||||||||||||||||||||||||||||||||||||||||||||||||||
Seq_2  3356  CCAATAACATTAGAATTGATAAATATAAGATCAATATGAAGCTTTCAAAAGATCCCAAGG  3415
              N  N  I  R  I  D  K  Y  K  I  N  M  K  L  S  K  D  P  K  A

              Y  P  G  G  K  N  M  P  A  V  Q  V  A  L  R  M  R  K  A  G
Seq_1  3416  CCTACCCAGGTGGTAAAAACATGCCTGCAGTCCAAGTAGCTCTAAGAATGCGTAAGGCTG  3475
             ||||||||||||||||||||||||||||||||||||||||||||||||||||||||||||
Seq_2  3416  CCTACCCAGGTGGTAAAAACATGCCTGCAGTCCAAGTAGCTCTAAGAATGCGTAAGGCTG  3475
              Y  P  G  G  K  N  M  P  A  V  Q  V  A  L  R  M  R  K  A  G

              R  V  V  K  A  G  S  V  I  T  F  V  I  T  K  Q  D  E  I  D
Seq_1  3476  GTAGAGTTGTTAAAGCTGGCTCTGTCATCACTTTTGTGATCACAAAGCAGGATGAAATAG  3535
             ||||||||||||||||||||||||||||||||||||||||||||||||||||||||||||
Seq_2  3476  GTAGAGTTGTTAAAGCTGGCTCTGTCATCACTTTTGTGATCACAAAGCAGGATGAAATAG  3535
              R  V  V  K  A  G  S  V  I  T  F  V  I  T  K  Q  D  E  I  D

              N  A  A  D  T  P  A  L  S  V  A  E  R  A  H  A  L  N  E  V
Seq_1  3536  ACAATGCAGCGGATACGCCGGCTCTTTCTGTGGCTGAACGTGCCCATGCATTGAATGAGG  3595
             |||||||||||||| | |||||||||||||||||||||||||||||||||||||||||||
Seq_2  3536  ACAATGCAGCGGATGCCCCGGCTCTTTCTGTGGCTGAACGTGCCCATGCATTGAATGAGG  3595
              N  A  A  D  A  P  A  L  S  V  A  E  R  A  H  A  L  N  E  V

              M  I  K  S  N  N  L  I  P  D  P  Q  Y  Y  L  E  K  Q  I  F
Seq_1  3596  TAATGATTAAAAGTAACAATTTGATACCTGATCCACAATATTATCTCGAGAAACAAATAT  3655
             ||||||||||||||||||||||||||||||||||||||||||||||||||||||||||||
Seq_2  3596  TAATGATTAAAAGTAACAATTTGATACCTGATCCACAATATTATCTCGAGAAACAAATAT  3655
              M  I  K  S  N  N  L  I  P  D  P  Q  Y  Y  L  E  K  Q  I  F

              A  P  V  E  R  L  L  E  R  I  D  S  F  N  V  V  R  L  S  E
Seq_1  3656  TCGCGCCGGTAGAAAGGTTGTTAGAAAGAATTGATAGCTTCAACGTGGTGCGTTTGAGTG  3715
             ||||||||||||||||||||||||||||||||||||||||||||||||||||||||||||
Seq_2  3656  TCGCGCCGGTAGAAAGGTTGTTAGAAAGAATTGATAGCTTCAACGTGGTGCGTTTGAGTG  3715
              A  P  V  E  R  L  L  E  R  I  D  S  F  N  V  V  R  L  S  E

              A  L  G  L  D  S  K  K  Y  F  R  R  E  G  G  N  N  N  G  E
Seq_1  3716  AAGCGCTTGGTTTAGATAGTAAAAAGTATTTTAGAAGAGAGGGCGGTAATAACAATGGAG  3775
             ||||||||||||||||||||||||||||||||||||||||||||||||||||||||||||
Seq_2  3716  AAGCGCTTGGTTTAGATAGTAAAAAGTATTTTAGAAGAGAGGGCGGTAATAACAATGGAG  3775
              A  L  G  L  D  S  K  K  Y  F  R  R  E  G  G  N  N  N  G  E

              D  I  N  N  L  Q  P  L  E  T  T  I  T  D  V  E  R  F  K  D
Seq_1  3776  AAGATATTAATAATTTGCAACCCTTAGAAACAACAATTACAGACGTTGAAAGGTTTAAGG  3835
             ||||||||||||||||||||||||||||||||||||||||||||||||||||||||||||
Seq_2  3776  AAGATATTAATAATTTGCAACCCTTAGAAACAACAATTACAGACGTTGAAAGGTTTAAGG  3835
              D  I  N  N  L  Q  P  L  E  T  T  I  T  D  V  E  R  F  K  D
```

```
                T  V  T  L  E  L  S  C  P  S  C  D  K  R  F  P  F  G  G  I
Seq_1   3836  ATACTGTAACATTAGAATTAAGTTGCCCATCATGCGATAAAAGGTTTCCATTTGGTGGTA  3895
              ||||||||||||||||||||||||||||||||||||||||||||||||||||||||||||
Seq_2   3836  ATACTGTAACATTAGAATTAAGTTGCCCATCATGCGATAAAAGGTTTCCATTTGGTGGTA  3895
                T  V  T  L  E  L  S  C  P  S  C  D  K  R  F  P  F  G  G  I

                V  S  S  N  Y  Y  R  V  S  Y  N  G  L  Q  C  K  H  C  E  Q
Seq_1   3896  TTGTATCTTCAAATTACTATCGCGTGTCATATAATGGTTTACAGTGCAAGCATTGTGAGC  3955
              ||||||||||||||||||||||||||||||||||||||||||||||||||||||||||||
Seq_2   3896  TTGTATCTTCAAATTACTATCGCGTGTCATATAATGGTTTACAGTGCAAGCATTGTGAGC  3955
                V  S  S  N  Y  Y  R  V  S  Y  N  G  L  Q  C  K  H  C  E  Q

                L  F  T  P  L  Q  L  T  S  Q  I  E  H  S  I  R  A  H  I  S
Seq_1   3956  AACTTTTTACTCCTCTTCAATTAACTAGCCAAATAGAGCATTCTATAAGGGCACACATTT  4015
              |||||||| | |||||||||||||||||||||||||||||||||||||||||||||||||
Seq_2   3956  AACTTTTTGCGCCTCTTCAATTAACTAGCCAAATAGAGCATTCTATAAGGGCACACATTT  4015
                L  F  A  P  L  Q  L  T  S  Q  I  E  H  S  I  R  A  H  I  S

                L  Y  Y  A  G  W  L  Q  C  D  D  S  T  C  G  I  V  T  R  Q
Seq_1   4016  CCTTATATTACGCAGGGTGGTTACAGTGTGATGACAGCACATGCGGTATAGTTACAAGAC  4075
              ||||||||||||||||||||||||||||||||||||||||||||||||||||||||||||
Seq_2   4016  CCTTATATTACGCAGGGTGGTTACAGTGTGATGACAGCACATGCGGTATAGTTACAAGAC  4075
                L  Y  Y  A  G  W  L  Q  C  D  D  S  T  C  G  I  V  T  R  Q

                V  S  V  F  G  K  R  C  L  N  D  G  C  T  G  V  M  R  Y  K
Seq_1   4076  AAGTCTCCGTTTTTGGTAAGCGTTGTTTGAATGACGGCTGCACGGGTGTCATGAGATACA  4135
              ||||||||||||||||||||||||||||||||||||||||||||||||||||||||||||
Seq_2   4076  AAGTCTCCGTTTTTGGTAAGCGTTGTTTGAATGACGGCTGCACGGGTGTCATGAGATACA  4135
                V  S  V  F  G  K  R  C  L  N  D  G  C  T  G  V  M  R  Y  K

                Y  S  D  K  Q  L  Y  N  Q  L  L  Y  F  D  S  L  F  D  C  E
Seq_1   4136  AATACAGTGACAAGCAATTGTACAATCAACTTTTATATTTCGATTCTTTATTCGATTGTG  4195
              ||||||||||||||||||||||||||||||||||||||||||||||||||||||||||||
Seq_2   4136  AATACAGTGACAAGCAATTGTACAATCAACTTTTATATTTCGATTCTTTATTCGATTGTG  4195
                Y  S  D  K  Q  L  Y  N  Q  L  L  Y  F  D  S  L  F  D  C  E

                K  N  K  K  Q  E  L  K  P  I  Y  L  P  D  D  L  D  Y  P  K
Seq_1   4196  AAAAAAATAAAAAGCAAGAATTGAAGCCAATATATCTACCCGATGATCTCGACTACCCCA  4255
              ||||||||||||||||||||||||||||||||||||||||||||||||||||||||||||
Seq_2   4196  AAAAAAATAAAAAGCAAGAATTGAAGCCAATATATCTACCCGATGATCTCGACTACCCCA  4255
                K  N  K  K  Q  E  L  K  P  I  Y  L  P  D  D  L  D  Y  P  K

                E  Q  L  T  E  S  S  I  K  A  L  T  E  Q  N  R  E  L  M  E
Seq_1   4256  AGGAACAGCTGACAGAATCATCTATTAAGGCTTTAACAGAACAAAACAGAGAACTAATGG  4315
              ||||||||||||||||||||||||||||||||||||||||||||||||||||||||||||
Seq_2   4256  AGGAACAGCTGACAGAATCATCTATTAAGGCTTTAACAGAACAAAACAGAGAACTAATGG  4315
                E  Q  L  T  E  S  S  I  K  A  L  T  E  Q  N  R  E  L  M  E

                T  G  R  S  V  V  Q  K  Y  L  N  D  C  G  R  R  Y  V  D  M
Seq_1   4316  AAACCGGCCGGAGCGTTGTTCAAAAATATTTGAACGATTGTGGACGTCGCTACGTTGATA  4375
              ||||||||||||||||||||||||||||||||||||||||||||||||||||||||||||
Seq_2   4316  AAACCGGCCGGAGCGTTGTTCAAAAATATTTGAACGATTGTGGACGTCGCTACGTTGATA  4375
                T  G  R  S  V  V  Q  K  Y  L  N  D  C  G  R  R  Y  V  D  M

                T  S  I  F  D  F  M  L  N  *
Seq_1   4376  TGACTAGCATATTTGATTTCATGCTAAATTAG  4407
              ||||||||||||||||||||||||||||||||
Seq_2   4376  TGACTAGCATATTTGATTTCATGCTAAATTAG  4407
                T  S  I  F  D  F  M  L  N  *
```

Appendix Figure S4: *POL1* sequence with *cdk13A* mutations and diagnostic restriction sites. WT Cdk site: **SP/TP.** Mutated Cdk site: **AP.**

```
Alignment of Sequence_1:  [POL12] with  Sequence_2: [pol12_cdk.xdna]

Similarity : 2057/2118 (97.12 %)

            M  S  G  S  I  D  V  I  T  H  F  G  P  D  A  D  K  P  E  I
Seq_1  1    ATGAGCGGATCTATCGATGTCATCACTCATTTTGGGCCTGATGCAGACAAGCCGGAAATC  60
            ||||||||||||||||||||||||||||||||||||||||||||||||||||||||||||
Seq_2  1    ATGAGCGGATCTATCGATGTCATCACTCATTTTGGGCCTGATGCAGACAAGCCGGAAATC  60
            M  S  G  S  I  D  V  I  T  H  F  G  P  D  A  D  K  P  E  I


            I  T  A  L  E  N  L  T  K  L  H  A  L  S  V  E  D  L  Y  I
Seq_1  61   ATCACTGCATTAGAGAATTTAACTAAACTGCATGCATTGTCCGTCGAGGATCTATACATT  120
            ||||||||||||||||||||||||||||||||||||||||||||||||||||||||||||
Seq_2  61   ATCACTGCATTAGAGAATTTAACTAAACTGCATGCATTGTCCGTCGAGGATCTATACATT  120
            I  T  A  L  E  N  L  T  K  L  H  A  L  S  V  E  D  L  Y  I


            K  W  E  Q  F  S  N  Q  R  R  Q  T  H  T  D  L  T  S  K  N
Seq_1  121  AAATGGGAGCAGTTCTCCAATCAAAGGCGTCAAACTCACACAGATCTGACTTCCAAGAAC  180
            ||||||||||||||||||||||||||||||||||||||||||||||||||||||||||||
Seq_2  121  AAATGGGAGCAGTTCTCCAATCAAAGGCGTCAAACTCACACAGATCTGACTTCCAAGAAC  180
            K  W  E  Q  F  S  N  Q  R  R  Q  T  H  T  D  L  T  S  K  N


            I  D  E  F  K  Q  F  L  Q  L  Q  M  E  K  R  A  N  Q  I  S
Seq_1  181  ATAGATGAGTTTAAACAATTTTTACAATTACAAATGGAAAAGCGTGCTAACCAAATTTCA  240
            ||||||||||||||||||||||||||||||||||||||||||||||||||||||||||||
Seq_2  181  ATAGATGAGTTTAAACAATTTTTACAATTACAAATGGAAAAGCGTGCTAACCAAATTTCA  240
            I  D  E  F  K  Q  F  L  Q  L  Q  M  E  K  R  A  N  Q  I  S


            S  S  S  K  V  N  T  S  T  K  K  P  V  I  K  K  S  L  N  S
Seq_1  241  TCGTCCTCTAAGGTAAACACATCCACAAAAAAACCAGTGATTAAAAAAAGTTTGAATTCA  300
            ||||||||||||||||||||||||||||||||||||||||||||||| |||||||||||
Seq_2  241  TCGTCCTCTAAGGTAAACACATCCACAAAAAAACCAGTGATTAAAAAGAGTTTGAATTCG  300
            S  S  S  K  V  N  T  S  T  K  K  P  V  I  K  K  S  L  N  S


            S  P  L  F  G  L  S  I  P  K  T  P  T  L  K  K  R  K  L  H
Seq_1  301  AGTCCCTTGTTTGGCCTTAGTATTCCAAAGACTCCGACTTTAAAAAAAAGAAAGTTACAT  360
               ||||||||||||||||||||||||||| | ||||||||||||||||||||||||||
Seq_2  301  GCGCCCTTGTTTGGCCTTAGTATTCCAAAGGCCCCGACTTTAAAAAAAAGAAAGTTACAC  360
            A  P  L  F  G  L  S  I  P  K  A  P  T  L  K  K  R  K  L  H

            G  P  F  S  L  S  D  S  K  Q  T  Y  N  V  G  S  E  A  E  T
Seq_1  361  GGTCCCTTTTCACTCAGTGATTCCAAGCAAACATATAATGTAGGCTCTGAGGCAGAAACA  420
            ||||| |||||||| |||||||||||||||||||||||||||||||||||||||||||||
Seq_2  361  GGTCCGTTTTCACTAAGTGATTCCAAGCAAACATATAATGTAGGCTCTGAGGCAGAAACA  420
            G  P  F  S  L  S  D  S  K  Q  T  Y  N  V  G  S  E  A  E  T


            N  E  K  G  N     S  S  L  K  L  E  F  T  P  G  M  A  E  D
Seq_1  421  AATGAAAAAGGAAA---TAGTTCTTTAAAATTGGAGTTTACTCCAGGCATGGCAGAAGAT  477
            ||||||||||||||    ||   |||||||||||||||| | ||||| ||||||||||||
Seq_2  421  AATGAAAAAGGAAACTCGAG---TTTAAAATTGGAGTTTGCACCAGGTATGGCAGAAGAT  477
            N  E  K  G  N  S  S     L  K  L  E  F  A  P  G  M  A  E  D
                          XhoI

            A  V  G  D  S  A  P   L  S  H  A  K  S  S  D  A  K  T  P  G
Seq_1  478  GCTGTGGGTGACAGTGCTCC-ATTGTCCCACGCTAAAAGCTCGGATGCCAAGACTCCCGG  536
            ||||||||||||||||||||   ||  ||||||||||||||||||||||||| |||||||
Seq_2  478  GCTGTGGGTGACAGTGCTCCGC-TGAGCCACGCTAAAAGCTCGGATGCCAAGGCTCCCGG  536
            A  V  G  D  S  A  P  L   S  H  A  K  S  S  D  A  K  A  P  G


             S  S  T  F  Q  T  P  T  T  N  T  P  T  T  S  R  Q  N  V  P
Seq_1  537  CTCGTCGACATTCCAAACTCCAACAACAAACACTCCTACCACATCGAGGCAAAATGTTCC  596
            ||| |||||||||||  | |||||||||||| | ||||||||||| || |||||||||||
Seq_2  537  CTCATCGACATTCCAGGCGCCAACAACAAACGCGCCTACCACATCTAGACAAAATGTTCC  596
             S  S  T  F  Q  A  P  T  T  N  A  P  T  T  S  R  Q  N  V  P
                                                       XbaI

             A  G  E  I  L  D  S  L  N  P  E  N  I  E  I  S  S  G  N  P
Seq_1  597  TGCTGGTGAAATTTTGGATTCTTTGAACCCTGAAAATATTGAAATATCATCTGGTAATCC  656
            ||||||||||||||||||||||||||||||||||||||||||||||||||||||||||||
Seq_2  597  TGCTGGTGAAATTTTGGATTCTTTGAACCCTGAAAATATTGAAATATCATCTGGTAATCC  656
             A  G  E  I  L  D  S  L  N  P  E  N  I  E  I  S  S  G  N  P
```

```
                N  V  G  L  L  S  T  E  E  P  S  Y  N  Q  V  K  V  E  P  F
Seq_1   657    AAATGTAGGCCTTCTTTCGACTGAAGAACCATCATATAACCAGGTCAAAGTAGAACCATT   716
               ||||||||||||||||||||||||||||||||||||||||||||||||||||||||||||
Seq_2   657    AAATGTAGGCCTTCTTTCGACTGAAGAACCATCATATAACCAGGTCAAAGTAGAACCATT   716
                N  V  G  L  L  S  T  E  E  P  S  Y  N  Q  V  K  V  E  P  F


                Y  D  A  K  K  Y  K  F  R  T  M  R  Q  N  L  Q  E  A  S  D
Seq_1   717    TTATGATGCTAAAAAGTATAAATTCAGAACAATGAGGCAAAACTTACAGGAAGCGTCTGA   776
               ||||||||||||||||||||||||| | || |||||||||||||||||||||||||||||
Seq_2   717    TTATGATGCTAAAAAGTATAAATTCCGTACGATGAGGCAAAACTTACAGGAAGCGTCTGA   776
                Y  D  A  K  K  Y  K  F  R  T  M  R  Q  N  L  Q  E  A  S  D
                                        BsiWI

                V  L  D  D  Q  I  E  S  F  T  K  I  I  Q  N  H  Y  K  L  S
Seq_1   777    TGTCCTCGATGATCAAATTGAATCGTTTACTAAGATTATTCAAAATCACTATAAGTTATC   836
               ||||||||||||||||||||||||||||||||||||||||||||||||||||||| || |
Seq_2   777    TGTCCTCGATGATCAAATTGAATCGTTTACTAAGATTATTCAAAATCACTATAAGCTAGC   836
                V  L  D  D  Q  I  E  S  F  T  K  I  I  Q  N  H  Y  K  L  A
                                                                     NheI

                P  N  D  F  A  D  P  T  I  Q  S  Q  S  E  I  Y  A  V  G  R
Seq_1   837    TCCAAACGACTTCGCTGATCCAACTATTCAATCTCAGTCCGAAATTTATGCTGTTGGAAG   896
               ||||||||||||||||||||||||||||||||||||||||||||||||||||||||||||
Seq_2   837    TCCAAACGACTTCGCTGATCCAACTATTCAATCTCAGTCCGAAATTTATGCTGTTGGAAG   896
                P  N  D  F  A  D  P  T  I  Q  S  Q  S  E  I  Y  A  V  G  R


                I  V  P  D  S  P  T  Y  D  K  F  L  N  P  E  S  L  S  L  E
Seq_1   897    GATAGTTCCAGATTCGCCGACCTACGACAAGTTTTTGAATCCAGAATCGCTTTCCCTAGA   956
               ||||||||||||  |||| || || |||||||||||||||||||||||||||||||||||
Seq_2   897    GATAGTTCCAGACGCGCCTACGTATGACAAGTTTTTGAATCCAGAATCGCTTTCCCTAGA   956
                I  V  P  D  A  P  T  Y  D  K  F  L  N  P  E  S  L  S  L  E
                                  SnaBI

                T  S  R  M  G  G  V  G  R  R  V  R  L  D  L  S  Q  V  N  E
Seq_1   957    GACCTCAAGAATGGGCGGCGTTGGAAGAAGGGTAAGGTTAGATCTATCGCAGGTCAACGA   1016
               ||||||||||||||||||||||||||||||||||||||||||||||||||||||||||||
Seq_2   957    GACCTCAAGAATGGGCGGCGTTGGAAGAAGGGTAAGGTTAGATCTATCGCAGGTCAACGA   1016
                T  S  R  M  G  G  V  G  R  R  V  R  L  D  L  S  Q  V  N  E


                L  S  F  F  L  G  Q  I  V  A  F  K  G  K  N  A  N  G  D  Y
Seq_1   1017   GCTTTCGTTTTTCTTAGGCCAAATTGTGGCATTCAAAGGTAAAAATGCTAATGGGGATTA   1076
               ||||||||||||||||||||||||||||||||||||||||||||||||||||||||||||
Seq_2   1017   GCTTTCGTTTTTCTTAGGCCAAATTGTGGCATTCAAAGGTAAAAATGCTAATGGGGATTA   1076
                L  S  F  F  L  G  Q  I  V  A  F  K  G  K  N  A  N  G  D  Y


                F  T  V  N  S  I  L  P  L  P  Y  P  N  S  P  V  S  T  S  Q
Seq_1   1077   CTTCACCGTTAACTCTATTTTGCCGCTACCTTATCCAAACTCCCCAGTGTCCACATCGCA   1136
               |||||||||||||||||||||||||||||||||||||||| | || || |||||    ||
Seq_2   1077   CTTCACCGTTAACTCTATTTTGCCGCTACCTTATCCAAACGCACCGGTTTCCACTAGTCA   1136
                F  T  V  N  S  I  L  P  L  P  Y  P  N  A  P  V  S  T  S  Q
                                                                   SpeI

                E  L  Q  E  F  Q  A  N  L  E  G  S  S  L  K  V  I  V  T  C
Seq_1   1137   GGAACTGCAGGAGTTTCAAGCAAATTTGGAAGGTTCTTCTTTGAAGGTTATCGTGACCTG   1196
               ||||||||||||||||||||||||||||||||||||||||||||||||||||||||||||
Seq_2   1137   GGAACTGCAGGAGTTTCAAGCAAATTTGGAAGGTTCTTCTTTGAAGGTTATCGTGACCTG   1196
                E  L  Q  E  F  Q  A  N  L  E  G  S  S  L  K  V  I  V  T  C


                G  P  Y  F  A  N  D  N  F  S  L  E  L  L  Q  E  F  I  D  S
Seq_1   1197   TGGCCCATACTTTGCAAATGATAACTTCTCCCTCGAATTGTTGCAAGAATTTATCGATAG   1256
               ||||||||||||||||||||||||||||||||||||||||||||||||||||||||||||
Seq_2   1197   TGGCCCATACTTTGCAAATGATAACTTCTCCCTCGAATTGTTGCAAGAATTTATCGATAG   1256
                G  P  Y  F  A  N  D  N  F  S  L  E  L  L  Q  E  F  I  D  S


                I  N  N  E  V  K  P  H  V  L  I  M  F  G  P  F  I  D  I  T
Seq_1   1257   TATAAATAATGAGGTAAAACCTCACGTCTTGATAATGTTTGGCCCATTTATAGATATCAC   1316
               ||||||||||||||||||||||||||||||||||||||||||||||||||||||||||||
Seq_2   1257   TATAAATAATGAGGTAAAACCTCACGTCTTGATAATGTTTGGCCCATTTATAGATATCAC   1316
                I  N  N  E  V  K  P  H  V  L  I  M  F  G  P  F  I  D  I  T
```

```
               H  P  L  I  A  S  G  K  L  P  N  F  P  Q  F  K  T  Q  P  K
Seq_1   1317  CCATCCTCTGATAGCGAGTGGTAAATTGCCAAATTTTCCCCAGTTCAAGACACAGCCCAA  1376
              ||||||||||||||||||||||||||||||||||||||||||||||||||||||||||||
Seq_2   1317  CCATCCTCTGATAGCGAGTGGTAAATTGCCAAATTTTCCCCAGTTCAAGACACAGCCCAA  1376
               H  P  L  I  A  S  G  K  L  P  N  F  P  Q  F  K  T  Q  P  K


               T  L  D  E  L  F  L  K  L  F  T  P  I  L  K  T  I  S  P  H
Seq_1   1377  AACATTGGACGAGCTTTTTTTGAAGTTATTTACTCCTATTCTAAAAACCATATCACCACA  1436
              ||||||||||||||||||| | ||||||||  | |||||||||||||| ||  |||||||
Seq_2   1377  AACATTGGACGAGCTTTTTCTTAAGTTATTCGCGCCTATTCTAAAAACGATCGCACCACA  1436
               T  L  D  E  L  F  L  K  L  F  A  P  I  L  K  T  I  A  P  H
                                  AflII                      PvuI

               I  Q  T  V  L  I  P  S  T  K  D  A  I  S  N  H  A  A  Y  P
Seq_1   1437  TATCCAAACCGTATTGATACCCTCCACAAAAGATGCAATTTCTAATCACGCCGCATATCC  1496
              ||||||||||||||||||||| || |||||||||||||||||||||||||||||||||||
Seq_2   1437  TATCCAAACCGTATTGATACCGTCGACAAAAGATGCAATTTCTAATCACGCCGCATATCC  1496
               I  Q  T  V  L  I  P  S  T  K  D  A  I  S  N  H  A  A  Y  P
                                   AflII

               Q  A  S  L  I  R  K  A  L  Q  L  P  K  R  N  F  K  C  M  A
Seq_1   1497  ACAAGCTTCTTTAATTAGAAAGGCTTTACAGCTGCCTAAAAGGAACTTCAAATGCATGGC  1556
              ||||||||||||||||||||||||||||||||||||||||||||||||||||||||||||
Seq_2   1497  ACAAGCTTCTTTAATTAGAAAGGCTTTACAGCTGCCTAAAAGGAACTTCAAATGCATGGC  1556
               Q  A  S  L  I  R  K  A  L  Q  L  P  K  R  N  F  K  C  M  A


               N  P  S  S  F  Q  I  N  E  I  Y  F  G  C  S  N  V  D  T  F
Seq_1   1557  TAATCCTTCATCTTTTCAAATAAATGAGATATACTTCGGTTGCTCAAACGTTGATACATT  1616
              ||||||||||||||||||||||||||||||||||||||||||||||||||||||||||||
Seq_2   1557  TAATCCTTCATCTTTTCAAATAAATGAGATATACTTCGGTTGCTCAAACGTTGATACATT  1616
               N  P  S  S  F  Q  I  N  E  I  Y  F  G  C  S  N  V  D  T  F


               K  D  L  K  E  V  I  K  G  G  T  T  S  S  R  Y  R  L  D  R
Seq_1   1617  CAAAGATCTAAAGGAAGTCATAAAAGGCGGTACTACCTCATCAAGATATAGATTAGATCG  1676
              ||||||||||||||||||||||||||||||||||||||||||||||||||||||||||||
Seq_2   1617  CAAAGATCTAAAGGAAGTCATAAAAGGCGGTACTACCTCATCAAGATATAGATTAGATCG  1676
               K  D  L  K  E  V  I  K  G  G  T  T  S  S  R  Y  R  L  D  R


               V  S  E  H  I  L  Q  Q  R  R  Y  Y  P  I  F  P  G  S  I  R
Seq_1   1677  TGTTTCTGAGCATATTTTACAACAGCGCAGGTATTACCCCATATTTCCAGGTAGTATTCG  1736
              ||||||||||||||||||||||||||||||||||||||||||||||||||||||||||||
Seq_2   1677  TGTTTCTGAGCATATTTTACAACAGCGCAGGTATTACCCCATATTTCCAGGTAGTATTCG  1736
               V  S  E  H  I  L  Q  Q  R  R  Y  Y  P  I  F  P  G  S  I  R


               T  R  I  K  P  K  D  V  S  T  K  K  E  T  N  D  M  E  S  K
Seq_1   1737  TACAAGAATAAAACCAAAAGACGTATCTACGAAGAAGGAAACCAATGATATGGAAAGCAA  1796
              ||||||||||||||||||||||||||||||||||||||||||||||||||||||||||||
Seq_2   1737  TACAAGAATAAAACCAAAAGACGTATCTACGAAGAAGGAAACCAATGATATGGAAAGCAA  1796
               T  R  I  K  P  K  D  V  S  T  K  K  E  T  N  D  M  E  S  K


               E  E  K  V  Y  E  H  I  S  G  A  D  L  D  V  S  Y  L  G  L
Seq_1   1797  AGAAGAAAAAGTTTATGAACATATATCTGGTGCAGATTTAGACGTGAGTTATCTGGGACT  1856
              ||||||||||||||||||||||||||||||||||||||||||||||||||||||||||||
Seq_2   1797  AGAAGAAAAAGTTTATGAACATATATCTGGTGCAGATTTAGACGTGAGTTATCTGGGACT  1856
               E  E  K  V  Y  E  H  I  S  G  A  D  L  D  V  S  Y  L  G  L


               T  E  F  V  G  G  F  S  P  D  I  M  I  I  P  S  E  L  Q  H
Seq_1   1857  AACAGAGTTTGTTGGTGGGTTCTCGCCTGACATAATGATTATACCCAGTGAATTACAACA  1916
              |||||||||||||||||||||| | || ||||||||||||||||||||||||||||||||
Seq_2   1857  AACAGAGTTTGTTGGTGGGTTCGCTCCGGACATAATGATTATACCCAGTGAATTACAACA  1916
               T  E  F  V  G  G  F  A  P  D  I  M  I  I  P  S  E  L  Q  H


               F  A  R  V  V  Q  N  V  V  V  I  N  P  G  R  F  I  R  A  T
Seq_1   1917  CTTTGCAAGAGTTGTCCAAAACGTAGTTGTTATAAATCCCGGAAGATTTATTAGAGCAAC  1976
              ||||||||||||||||||||||||||||||||||||||||||||||||||||||||||||
Seq_2   1917  CTTTGCAAGAGTTGTCCAAAACGTAGTTGTTATAAATCCCGGAAGATTTATTAGAGCAAC  1976
               F  A  R  V  V  Q  N  V  V  V  I  N  P  G  R  F  I  R  A  T
```

```
                G  N  R  G  S  Y  A  Q  I  T  V  Q  C  P  D  L  E  D  G  K
Seq_1   1977  AGGTAACAGGGGATCCTATGCACAAATTACTGTCCAATGCCCTGATCTTGAAGACGGGAA  2036
              ||||||||||||||||||||||||||||||||||||||||||||||||||||||||||||
Seq_2   1977  AGGTAACAGGGGATCCTATGCACAAATTACTGTCCAATGCCCTGATCTTGAAGACGGGAA  2036
                G  N  R  G  S  Y  A  Q  I  T  V  Q  C  P  D  L  E  D  G  K

                L  T  L  V  E  G  E  E  P  V  Y  L  H  N  V  W  K  R  A  R
Seq_1   2037  ATTGACGCTTGTTGAAGGTGAAGAGCCAGTTTATCTACACAACGTGTGGAAGCGCGCTAG  2096
              ||||||||||||||||||||||||||||||||||||||||||||||||||||||||||||
Seq_2   2037  ATTGACGCTTGTTGAAGGTGAAGAGCCAGTTTATCTACACAACGTGTGGAAGCGCGCTAG  2096
                L  T  L  V  E  G  E  E  P  V  Y  L  H  N  V  W  K  R  A  R

                V  D  L  I  A  S  *
Seq_1   2097  AGTTGACTTGATTGCTAGTTGA  2118
              ||||||||||||||||||||||
Seq_2   2097  AGTTGACTTGATTGCTAGTTGA  2118
                V  D  L  I  A  S  *
```

Appendix Figure S5: *POL12* sequence with *cdk12A* mutations and diagnostic restriction sites. WT Cdk site: **SP/TP.** Mutated Cdk site: **AP.** Silent mutation**.**
