# Supplementary material for: Switch from canonical to recombination-dependent DNA synthesis by M-CDK phosphorylation of DNA Polα
Source: EMBO J. 2026 Oct 10;45(21):7621–46. doi: 10.1038/s44318-026-00919-5 (PMC13728163; doi:10.1038/s44318-026-00919-5)
Supplement: Supplementary file 3 [file 44318_2026_919_MOESM2_ESM.pdf]

Table EV1: Pol1 and Pol12 phospho-peptides identified in mitotic *cdc6-1* cells at 30°C.

Position Pol1	Phosphopeptide	Probability	Predicted
3	S**S**KSEKLEK	0.59	-
5	SSK**S**EKLEK	0.99	-
31	NGTSIDDYEGDE**S**DGDRIYDEIDEK	0.99	**Cdc5**
149	SIPIDNFDDILGEFE**S**GEVEKPNILLPSK	0.99	-
169	ENLN**S**SPTSEFK	0.97	-
170	ENLNS**S**PTSEFK	0.99	**Cdk**
172	LRENLNSSP**T**SEFK	0.53	-
188	VNGNDE**S**SHDAGISK	0.75	**Cdc5**
189	RVNGNDES**S**HDAGISK	0.49	-
204	VKIDPD**S**STDKYLEIESSPLK	0.66	-
205	VKIDPDS**S**TDKYLEIESSPLK	0.66	-
206	VKIDPDSS**T**DKYLEIESSPLK	0.66	-
215	YLEIES**S**PLK	0.72	**Cdk**
240	YANDVQDLLDDVEN**S**PVVATK	0.99	**Cdk**
274	QNVLQDTLLANPPSAQSLADEEDDED**S**DEDIILKR	0.99	**Cdc5**
300	**S**NPSTSPFVTAPGTPIGIK	0.99	-
303	SNP**S**TSPFVTAPGTPIGIK	0.57	-
304	SNPS**T**SPFVTAPGTPIGIK	0.57	-
305	SNPST**S**PFVTAPGTPIGIK	0.88	**Cdk**
313	SNPSTSPFVTAPG**T**PIGIK	0.99	**Cdk**
321	GL**T**PSKSLQSNTDVATLAVNVK	0.80	**Cdk**
323	GLTP**S**KSLQSNTDVATLAVNVK	0.19	-
325	GLTPSK**S**LQSNTDVATLAVNVK	0.19	**Cdc5**
328	SLQ**S**NTDVATLAVNVK	0.99	-
330	GLTPSKSLQSN**T**DVATLAVNVK	0.19	-
560	ENKQEIV**S**ITLSAYR	0.56	**Cdc5**
562	ENKQEIVSI**T**LSAYR	0.53	**Cdc5**
568	NI**S**LDSPIPENIKPDDLCTLVRPPQSTSFPLGLAALAK	0.54	**Cdc5**
1231	ID**S**FNVVR	1	-

Position Pol12	Phosphopeptide	Probability	Predicted
80	ANQI**S**SSSKVNTSTK	0.49	-
81	ANQIS**S**SSKVNTSTK	0.83	-
82	ANQISS**S**SKVNTSTK	0.8	-
83	ANQISSS**S**KVNTSTK	0.49	-
87	VNT**S**TKKPVIK	0.87	-
88	VNTS**T**KKPVIK	0.98	-
97	K**S**LNSSPLFGLSIPK	0.48	**Cdc5**
100	KSLN**S**SPLFGLSIPK	0.99	-
101	KSLNS**S**PLFGLSIPK	0.99	**Cdk**
111	KSLNSSPLFGLSIPK**T**PTLK	0.89	**Cdk**
124	KLHGPF**S**LSDSK	0.99	**Cdc5**
126	LHGPFSL**S**DSK	0.99	-
128	KLHGPFSLSD**S**KQTYNVGSEAETNEK	0.25	-
131	KLHGPFSLSDSKQ**T**YNVGSEAETNEK	0.25	-
136	QTYNVG**S**EAETNEK	0.95	-
146	GN**S**SLKLEFTPGMAEDAVGDSAPLSHAK	0.53	-
147	GNS**S**LKLEFTPGMAEDAVGDSAPLSHAK	0.44	-
153	GNSSLKLEF**T**PGMAEDAVGDSAPLSHAK	0.99	**Cdk1**
168	LEFTPGMAEDAVGDSAPL**S**HAK	0.99	-
180	TPG**S**STFQTPTTNTPTTSR	0.90	-
181	TPGS**S**TFQTPTTNTPTTSR	0.53	-
182	TPGSS**T**FQTPTTNTPTTSR	0.36	-
185	TPGSSTFQ**T**PTTNTPTTSR	0.99	**Cdk**
188	TPGSSTFQTPT**T**NTPTTSR	0.56	-
190	TPGSSTFQTPTTN**T**PTTSR	0.82	**Cdk**
304	IVPD**S**PTYDK	0.99	**Cdk**
