# Supplementary material for: Switch from canonical to recombination-dependent DNA synthesis by M-CDK phosphorylation of DNA Polα
Source: EMBO J. 2026 Oct 10;45(21):7621–46. doi: 10.1038/s44318-026-00919-5 (PMC13728163; doi:10.1038/s44318-026-00919-5)
Supplement: Supplementary file 4 [file 44318_2026_919_MOESM3_ESM.pdf]

Table EV2: Yeast strains used in this study

Strain	**Genotype** (W303 *RAD5* background, unless stated otherwise)	Figure	Notes
E1266	*MATa, chk1::LEU2, rad53-11*	5E	1
E1299	*MATa, URA3::TUB1-GFP, rad53-11*	5E	
E1760	*MATa, CAN1, hxt13::URA3*	3D	1, 2
E1782	*MATa, CAN1, hxt13::URA3, cdc6-1*	3D	1, 2
E1878	*MATa, CAN1, hxt13::URA3, sic1::HIS3*	3D	1, 2
E2437	*MATa, bub1::HIS5, CEN5-GFP*	EV6B	1
E2885	*MATα, cdc6-1, rad53-11*	5E	1
E2888	*MATα, cdc6-1, rad53-11, chk1::LEU2*	5E	1
E3087	*MATa, URA3::GPD-TK(5x), AUR1c::ADH-hENT1*	1B,C,E,F; 2B,C; 3A; 4B; EV1A-E; EV3B; Appendix Figures S1-S3	
E3379	*MATa, cdc6-1, URA3::GPD-TK(5x), AUR1c::ADH-hENT1*	1B-D	
E3403	*MATa, cdc6-1, RAD52-GFP, URA3::mCherry-TUB1*	2A,E,F; 4D; 5B; 6C; EV3E; EV5F	1
E3408	*MATa, RAD52-GFP, URA3::mCherry-TUB1*	2A; 4D; 6C; EV3E	1
E3410	*MATa/α, cdc6-1/cdc6-1, RAD52-GFP/RAD52-GFP, URA3::mCherry-TUB1/URA3::mCherry-TUB1*	Movie EV1, EV2	1
E3642	*MATa, cdc6-1, rad51::LEU2, RAD52-GFP, URA3::mCherry-TUB1*	EV5F	1
E3645	*MATα, cdc6-1, RAD52-GFP, URA3::mCherry-TUB1*	5E	
E3680	*MATa, cdc6-1, cdc15-2, URA3::mCheTUB1, RAD52-GFP*	2E	1
E3689	*MATa, cdc6-1, URA3::GAL-SWE1, RAD52-GFP*	2D	1
E3690	*MATa, cdc6-1, URA3::GAL-SWE1, RAD52-GFP*	2E	1
E3692	*MATa, URA3::GAL-SWE1(1x), RAD52-GFP*	6C	1
E3732	*MATa, cdc6-1, MET-HA3-CDC20::TRP1, URA3::mCheTUB1, RAD52-GFP*	2E	1
E3879	*MATa, cdc6-1, rad51::LEU2, URA3::GPD-TK(5x), AUR1c::ADH-hENT1*	3C	
E3888	*MATa, cdc6-1, URA3::GPD-TK(5x), AUR1c::ADH-hENT1*	2B,D; 3A; 5A	1
E3898	*MATa, cdc6-1, cdc5::ura4, LEU2::MET-CDC5, RAD52-GFP, URA3::mCherry-TUB1*	2G	
E4029	*MATa, cdc6-1, cdc28-as1, RAD52-GFP, URA3::mCherry-TUB1*	2F	1
E4108	*MATa, RFA1-CFP*	2A	
E4168	*MATα, cdc6-1, pol32::KanMX4, RAD52-GFP, URA3::mCherry-TUB1*	EV5F	
E4513	*MATa, cdc6-1, RAD52-GFP, URA3::mCherryTUB1, pol12-cdk12A*	EV5D	
E4518	*MATa, RAD52-GFP, URA3::mCherryTUB1, pol1-cdk13A*	EV5D	
E4519	*MATa, RAD52-GFP, URA3::mCherryTUB1, pol12-cdk12A*	EV5D	
E4521	*MATa, cdc6-1, RAD52-GFP, URA3::mCherryTUB1, pol1-cdk13A*	EV5D	
E4522	*MATa, cdc6-1, RAD52-GFP, URA3::mCherryTUB1, pol1-cdk13A, pol12-cdk12A*	4D; EV5D	1
E4523	*MATa, RAD52-GFP, URA3::mCherryTUB1, pol1-cdk13A, pol12-cdk12A*	4D; EV5D	
E4572	*MATa, cdc6-1, RAD52-GFP, URA3::mCherryTUB1, pol1-cdk13A, pol12-cdk12A*	5A,B; 6C	1, 2
E4575	*MATa, RAD52-GFP, URA3::mCherryTUB1, pol1-cdk13A, pol12-cdk12A*	4A,B	1
E4701	*MATa*	5E,F; EV5B,C,F; EV6B,C	
E4713	*MATa, URA3::GAL-SWE1, TRP1::ADH1pr- OsTIR1-myc9, cdc5-mAID3::NatNT, POL32-6HA::HIS3MX6, RAD52-GFP*	4A	
E4887	*MATa, rad52::TRP1*	EV5F	
E4889	*MATa, cdc6-1, rad52::TRP1*	EV5F	
E4899	*MATa, cdc6-1*	EV5F	
E5124	*MATa/α, cdc6-1/cdc6-1, LEU2/leu2Δ1, ura3-52, trp1Δ63/TRP1, HIS3/his3Δ200, ade2Δ::hisG/id, III-205::URA3, III-314::ADE2*	3E; EV3D	1, 2
E5317	*MATa, cdc6-1, pol1-cdk13A, pol12-cdk12A, RFA1-CFP, URA3::mCherryTUB1*	5B	
E5585	*MATα, pol1-cdk13A, pol12-cdk12A*	EV5B,C,E	
E5793	*MATa, cdc6-1, pol1-cdk13A, pol12-cdk12A, URA3::GPD-TK (5 copies), AUR1c::ADH-hENT1*	5C,D,F; EV6A,C	
E5797	*MATa, cdc6-1, RFA1-CFP, URA3::mCherryTUB1*	2A; 5B	

E5834	*MATa, ade2-1, trp1-1, can1-100, leu2-3,112, his3-11,15, ura3-1, POL12-3xPK, GAL, psi+, RAD5+*	4B	
E5956	*MATa, cdc6-1, URA3::GPD-TK(5x), AUR1c::ADH-hENT1*	1E,F; 2C; 3B,C; 5C,D,F; EV1C-E; EV2C; EV3B,C; EV4F,G;EV6A,C; Appendix Figures S1-S3	
E5964	*MATa, cdc6-1, LEU2::GPD1-OsTIR1, DNA2-AID*-myc9::HYG, mcm4::MCM4-5FLAG (hphNT), POL1-3PK, CTF4-6HA::HIS3*	6A,B	
E6004	*MATa, cdc6-1, LEU2::GPD1-OsTIR1, DNA2-AID*-myc9::HYG, mcm4::MCM4-5FLAG (hphNT), CTF4-6HA::HIS3, pol1-cdk13A-3PK, pol12-cdk12A*	6A,B	
E6012	*MATa, cdc6-1, leu2::GPD1-OsTIR::LEU2, DNA2-AID*-myc9::HYG, mcm4::MCM4-5FLAG (hphNT), CTF4-6HA::HIS3*	6A,B	
E6098	*MATa, cdc6-1, DNA2-AID*-myc9::HYG, CTF4-6HA::HIS3, POL12-3PK, POL1-3PK, mcm4::MCM4-5FLAG (hphNT)*	4C, Table EV1	
E6272	*MATa, pol1-cdk13A, pol12-cdk12A, URA3::GPD-TK, AUR1c::ADH-hENT1*	EV5A	
E6274	*MATa, pol12-cdk12A, URA3::GPD-TK, AUR1c::ADH-hENT1*	EV5A	
E6276	*MATa, pol1-cdk13A, URA3::GPD-TK, AUR1c::ADH-hENT1*	EV5A	
E6354	*MATa, cdc6-1, URA3::GPD-TK(5x), AUR1c::ADH-hENT1, pol32Δ::KanMX4*	3C, EV4F	
E6484	*MATα, sid2-21 (cdt1-21), pol1-cdk13A, pol12-cdk12A*	EV5E	
E6497	*MATα, sid2-21 (cdt1-21)*	EV5E	
E6527	*MATα, cdc6-1, RFA1-CFP, RAD52-mcherry*	EV2A,B	
E6557	*MATa, sid2-21 (cdt1-21), URA3::GPD-TK(5x), AUR1c::ADH-hENT1*	EV1D	
E6855	*MATa, cdc6-1, mus81Δ::KanMX4, URA3::GPD-TK(5x), AUR1c::ADH-hENT1*	EV4G	
E6716	*MATa, cdc6-1, mph1::KanMX4, URA3::GPD-TK(5x), AUR1c::ADH-hENT1*	EV4F	
E6722	*MATa, cdc6-1, sgs1::HIS3, URA3::GPD-TK(5x), AUR1c::ADH-hENT1*	EV4F	
E6725	*MATa, cdc6-1, srs2::KanMX4, URA3::GPD-TK(5x), AUR1c::ADH-hENT1*	EV4F	
E6727	*MATa, cdc6-1, exo1::KanMX4, URA3::GPD-TK(5x), AUR1c::ADH-hENT1*	EV4F	
E7008	*MATa, RAD52-aid, Gal-OsTIR1, cdc6-1, GPD-TK(5x), ADH-hENT1*	EV4A-E	
E7015	*MATa, cdc6-1, MET-CDC20, pol1-cdk13A, pol12-cdk12A*	EV6D,E	
E7020	*MATa, cdc6-1, MET-CDC20*	EV6D,E	
**Notes**	1) Contains the hypomorph *rad5-535* allele originally present in W303; 2) Backcrossed at least 3 times to W303		
