# Supplementary material for: Switch from canonical to recombination-dependent DNA synthesis by M-CDK phosphorylation of DNA Polα
Source: EMBO J. 2026 Oct 10;45(21):7621–46. doi: 10.1038/s44318-026-00919-5 (PMC13728163; doi:10.1038/s44318-026-00919-5)
Supplement: Supplementary file 5 [file 44318_2026_919_MOESM4_ESM.pdf]

THE EMBO JOURNAL

Switch from canonical to recombination-dependent DNA synthesis by M-CDK phosphorylation of DNA Pola

Juan de Dios Barba Tena, Riccardo Montecchi, Maxime Lambert, Thomas Defrance, Elisabeth Gueydon, Julie Petit, Marjorie Drac, Noémie Legoff, Michella Galli, Isabelle Goiffon, Thierry Gostan, Khadija El Koulali, Serge Urbach, David Toczyski, Nicolas Talarek, and Etienne Schwob

Corresponding author(s): Etienne Schwob (etienne.schwob@igmm.cnrs.fr) , Nicolas Talarek (nicolas.talarek@igmm.cnrs.fr)

Review Timeline:

Submission Date:	23rd Apr 26
Editorial Decision:	19th May 26
Revision Received:	24th Aug 26
Editorial Decision:	7th Sep 26
Revision Received:	14th Sep 26
Accepted:	15th Sep 26

Editor: Hartmut Vodermaier

Transaction Report:

(Note: With the exception of the correction of typographical or spelling errors that could be a source of ambiguity, letters and reports are not edited. Depending on transfer agreements, referee reports obtained elsewhere may or may not be included in this compilation. Referee reports are anonymous unless the Referee chooses to sign their reports.)

Dr. Etienne Schwob
Institut de Génétique Moléculaire (IGMM)
CNRS, Univ Montpellier
1919, route de Mende
Montpellier 34293
France

19th May 2026

Re: EMBOJ-2026-124453
Switch from canonical to recombination-dependent DNA synthesis by M-CDK phosphorylation of DNA Pola

Dear Etienne,

Thank you for submitting your manuscript on replication origin under-licensing in yeast for our consideration. I sent it to three expert referees, who have now returned the reports that are copied below. Since all reviewers acknowledge the interest of the subject and of your new findings, and also consider the study overall well done, we would be happy to pursue a revised version further for EMBO Journal publication. Nevertheless, each referee notes a number of conclusions about which they are not yet fully convinced; and while these issues may be to some extent addressed by additional discussion or toning down, I feel it would be important to also follow-up on some of them experimentally. In this light, I would invite you to contact me with a tentative point-by-point response and revision plan within the next few weeks, proposing how you could envision addressing the various concerns raised in the reports; based on this, we could then define more concrete plans and requirements for a successful revision. We would also be open to extension of the regular three-months revision period if needed; our 'scooping protection' (meaning that competing work appearing elsewhere in the meantime will not affect our considerations of your study) would of course remain valid also throughout such an extension.

Further information on preparing, formatting and uploading a revised manuscript can be found below and in our Guide to Authors. Thank you again for the opportunity to consider this work for The EMBO Journal, and I look forward to hearing from you soon.

With kind regards,

Hartmut

Hartmut Vodermaier, PhD
Senior Editor, The EMBO Journal
h.vodermaier@embojournal.org

*** PLEASE NOTE: All revised manuscript are subject to initial checks for completeness and adherence to our formatting guidelines. Revisions may be returned to the authors and delayed in their editorial re-evaluation if they fail to comply to the following requirements. As a first step please read our guidelines for revised submissions:
https://link.springer.com/journal/44318/submission-guidelines#cms-Revised-submissions

1) Every manuscript requires a Data Availability section (even if only stating that no deposited datasets are included). Primary datasets or computer code produced in the current study have to be deposited in appropriate public repositories prior to resubmission, and reviewer access details provided in case that public access is not yet allowed.

2) Each figure legend must specify
- size of the scale bars that are mandatory for all micrograph panels
- the statistical test used to generate error bars and P-values
- the type error bars (e.g., S.E.M., S.D.)
- the number (n) and nature (biological or technical replicate) of independent experiments underlying each data point
- Figures may not include error bars for experiments with n<3; scatter plots showing individual data points should be used instead.

3) Revised manuscript text (including main tables, and figure legends for main and EV figures) has to be submitted as editable text file (e.g., .docx format). We encourage highlighting of changes (e.g., via text color) for the referees' reference.

4) Each main and each Expanded View (EV) figure should be uploaded as individual production-quality files (preferably in .eps,

.tif, .jpg formats). For suggestions on figure preparation/layout, please refer to our Figure Preparation Guidelines: https://media.springernature.com/original/springer-cms/rest/v1/content/27825798/data/v1

5) Point-by-point response letters should include the original referee comments in full together with your detailed responses to them (and to specific editor requests if applicable), and also be uploaded as editable (e.g., .docx) text files.

6) Please complete our Author Checklist, and make sure that information entered into the checklist is also reflected in the manuscript; the checklist will be available to readers as part of the Review Process File.

7) All authors listed as (co-)corresponding need to deposit, in their respective author profiles in our submission system, a unique ORCiD identifier linked to their name. Please see our Guide to Authors for detailed instructions.

8) Please note that supplementary information at EMBO Press has been superseded by the 'Expanded View' for inclusion of additional figures, tables, movies or datasets; with up to five EV Figures being typeset and directly accessible in the HTML version of the article.

9) To facilitate reproducibilty and cross-laboratory adoption of methodologies, please structure the Materials & Methods section as outlined in our guide to authors, including a completed Reagents and Tools Table.

10) Digital image enhancement is acceptable practice, as long as it accurately represents the original data and conforms to community standards. If a figure has been subjected to significant electronic manipulation, this must be clearly noted in the figure legend and/or the 'Materials and Methods' section. The editors reserve the right to request original versions of figures and the original images that were used to assemble the figure. Finally, we generally encourage uploading of numerical as well as gel/blot image source data.

At EMBO Press we ask authors to provide source data for the main manuscript figures. You will receive a separate email with instructions for providing source data with your revised manuscript, including how to upload and organize the files.

In the interest of ensuring the conceptual advance provided by the work, we recommend submitting a revision within 3 months (17th Aug 2026). Please discuss the revision progress ahead of this time with the editor if you require more time to complete the revisions. Use the link below to submit your revision:

Link Not Available

Referee #1:

This study addresses how cells handle entry into mitosis with incompletely replicated chromosomes and its consequences for genome stability. Using yeast origin licensing mutants, the authors show that under-licensed cells enter mitosis without checkpoint arrest, where M-CDKs phosphorylate Polα, displacing it from the replisome and triggering a DDR that enables BIR-like DNA synthesis.
This is a thoughtful and technically strong study that tackles an important question in chromosome biology. The experiments are carefully designed, and the identification of Polα phosphorylation as a regulatory switch is particularly compelling. I have several suggestions that I believe will help strengthen the manuscript and clarify some of the interpretations.

specific concerns
The global depletion of replication origins in a synchronized yeast population represents a substantially different scenario from the localized origin scarcity observed at human common fragile sites. While the parallels to cancer biology are interesting, they currently feel somewhat speculative, especially given the noted mechanistic differences (e.g., lack of DSBs and Mus81-Mms4 dependence in yeast MiDAS). It would strengthen the manuscript to more clearly acknowledge these distinctions and to temper the translational implications.

The nocodazole experiment in cdc6-1 polα-cdk25A cells is elegant and suggests that extending metaphase can rescue viability. However, since nocodazole activates the spindle assembly checkpoint rather than acting as a simple timer, it may have additional effects on DNA damage signaling or replication intermediates. The interpretation would be strengthened either by testing alternative ways of prolonging metaphase or by more explicitly discussing this caveat.

The manuscript proposes that BIR-like MiDAS completes replication of a substantial fraction of the genome (~30%), while also reporting an approximately 800-fold increase in GCR rates. It would be helpful to further discuss how cells tolerate such elevated genome instability across divisions, and how these observations can be reconciled.

The conclusion that origin firing efficiency is the primary determinant of replication timing is stated strongly but is based mainly on the observed flattening of timing profiles in under-licensed cells. While consistent with this model, other contributing factors such as chromatin organization, transcription, and nuclear architecture are not examined. A more balanced framing of this conclusion would improve the manuscript.

Given the cell-to-cell variability in S-phase progression and mitotic entry, there is a possibility that the BrdU pulse used to detect MiDAS (80-135 min) captures residual late S-phase synthesis. Although morphology-based gating is helpful, it may not fully exclude this possibility. Additional clarification or discussion would be valuable.

The observation that Cdc5 depletion reduces Rad52 foci in cdc6-1 cells is intriguing but not fully integrated into the model. If Cdc5/Plk1 contributes to Polα phosphorylation alongside M-CDK, this could have implications for the model in Figure 6D. It would be helpful to either explore this further or explicitly note it as an open question.

Several strains used in the GCR assays carry the rad5-535 allele, which affects post-replication repair. As BIR intermediates may intersect with this pathway, it would be useful to discuss whether this genetic background could influence the elevated GCR rates observed in cdc6-1 cells.

Referee #2:

In this study, the authors investigated the consequences of replication origin under licensing in budding yeast. They show that cdc6-1 or cdt1-21 conditional mutants, when grown under semi-permissive conditions, have a lower number of active origins and a prolonged S-phase resulting in mitotic entry before S-phase completion. Surprising, they do not observe activation of the DNA replication or damage checkpoint (DDC) until mitotic entry. Preventing mitotic entry by over-expression of SWE1 or by use of the cdcd28-as1 allele averted DNA damage and checkpoint activation suggesting forks collapse on mitotic entry. Consistent with fork collapse, cdc6-1 cells exhibit elevated frequencies of chromosome rearrangements and mitotic crossovers/break-induced replication (BIR) at the semi-permissive temperature. In contrast to wild-type cells, cdc6-1 cells exhibit DNA synthesis tracts in mitosis, reminiscent of the aberrant form of DNA synthesis observed at common fragile sites in human cells, referred to as MiDAS. These observations are consistent fork breakage at under-replicated regions on mitotic entry and subsequent repair by recombination-dependent DNA synthesis. The authors identify the Pol1 and Pol12 subunits of DNA Pol alpha as targets for M-CDK phosphorylation, and subsequently show that mutating these sites to alanines prevents DDC activation, reduces viability and EdU incorporation in mitosis in cdc6-1 cells and leads to premature mitotic exit. Furthermore, they find that Pol alpha phosphorylation by M-CDK in mitotic cells disrupts its interaction with other replisome components, suggesting that replication fork uncoupling is likely responsible for checkpoint activation and initiation of MiDAS.

This is an interesting study that provides a mechanistic understanding for how MiDAS initiates and could potentially explain the higher mutagenic burden of late-replicating regions of the genome. The experimental data are convincing and I have no major concerns with the conclusions.

Minor comments:

Figure 3C: Was MiDAS detected in cdc6-1 rad52 cells? Cells lacking Rad52 generally exhibit stronger HR defects and are defective for the residual BIR detected in rad51 and pol32 cells. Do the rad51 and pol32 mutations reduce viability of cdc6-1 cells grown at the semi-permissive temperature?

Line 233 and Figure S3D: The Hiraoka et al study reported that ~ 10% of mitotic crossovers/BIR are due to ectopic recombination between repeated sequences so these events are not that rare. Although the PFG does show an aberrant karyotype for 5/11 events tested it is not clear that these are due to translocations involving chromosome III because both copies of chromosome III appear to be present in some lanes labeled as TL (#4, #6).

Line 304: The authors suggest that Pol alpha phosphorylation does not promote MiDAS directly and is mainly to trigger the checkpoint to arrest cell division to allow time for MiDAS to occur. Studies of BIR at endonuclease-induced double-strand breaks (DSBs) have shown an essential role for Pol alpha-primase to complete BIR (Lydeard et al., 2007; Donnianni et al., 2019), presumably to prime synthesis of the second strand. It would be of interest to determine whether the pol alpha-cdk25A mutant is proficient for BIR at DSBs.

Line 472 and Figure 6D: Presumably, the initiation of MiDAS would require fork breakage to generate an end for strand invasion. The authors state that yeast cells perform MiDAS independently of the Mus81-Mms4 nuclease. There is no reference for this statement or data addressing this point in the manuscript. Yen1 would likely not be activated at the time MiDAS initiates, but Slx1 or Rad1 might be candidated for cleavage of uncoupled forks. I'm not suggesting that the authors test these candidates but there should be some discussion of this point.

Referee #3:

This paper examines what happens in budding yeast when the total number of replication origins is reduced by partially inhibiting the replication licensing machinery. As has been shown in animal cells, this causes a significant increase in mitotic DNA synthesis. Interestingly, the paper goes on to show that this causes hyperphosphorylation of DNA polymerase alpha and its removal from chromatin, with the mitotic DNA synthesis being dependent on genes involved in break-induced replication. This provides interesting new detail about mitotic DNA synthesis, and suggests conservation of mechanism across the eukaryotic kingdom. The work merits publication.

Overall the data are strong and support the central hypothesis. However, there are two areas where I thought the authors have pushed the interpretation of the data a little too far; neither of these cases however undermine the central thesis as outlined above. My criticisms can be addressed by either softening the wording or addition of a few simple controls.

1. In two places, the authors comment on how they think that under-licensing caused by the cdc6-1 mutation affects the replication programme. They draw conclusions based on a relatively superficial analysis of the DNAscent data in Figure 1 and S1. Line 131 "Unexpectedly, the late/inefficient origins fired more frequently in cdc6-1 cells at 30{degree sign}C." Line 351: "An unexpected finding is that origin under-licensing obliterates the stereotypical replication timing program shared by most cells in the population (Figure 1F)." Without more analysis of the DNAscent data I don't think these statements are justified, just because the peaks have become more flattened. At most, it may be possible that the difference between early and late origins is reduced and they now fire more equivalently. I think it is enough to simply says that the replication programme is clearly changed from normal.

2. In Figure 2 the authors use a couple of approaches to inhibit CDC28 in order to conclude that mitotic entry is responsible for RAD53 phosphorylation. But since CDC28 is also responsible for S phase entry and origin firing as well as mitotic entry, I don't find this data convincing. I would have thought that SWE1 overexpression would inhibit the S phase function of CDC28 as well as its mitotic function. 1-NMPP1 inhibition of cdc28-as1 is better evidence, because 1-NMPP1 added after alpha factor release should allow the majority of origins to fire. If the authors want to maintain this argument, DNA content analysis by flow cytometry should at the minimum be shown. Even so, a better approach would be use of a mutant that really should not itself interfere with DNA replication (CDC5/Polo or MIH1/CDC25?).

Minor points:

3. I presume controls were done for Fig 2E that CDC20 and CDC15 mutations did indeed block anaphase and cell division?
4. Figure 3C: why are ~10% of cells scored as Mitotic EdU+ even in the absence of EdU? This seems like a very high false positive rate.
5. Line 401: "Our study uncovers the long-sought role of Pola phosphorylation,". They shown one role, but there could be others.

Manuscript EMBOJ-2026-124453 – Rebuttal letter

Switch from canonical to recombination-dependent DNA synthesis
by M-CDK phosphorylation of DNA Polα
Barba Tana JD et al.

We thank the referees for their fair review, insightful suggestions and positive assessment of our manuscript.
To address their specific concerns and curiosity, we have performed additional experiments & analyses to:

1. Quantify the alterations of chromosome replication timing induced by origin under-licensing: we measured the area under the curve (AUC) for early- and late-replicating domains on chromosome replication profiles generated by BrdU-nanoporeSeq for WT and *cdc6-1* cells grown at 25°C and 30°C. While no significant changes are seen at permissive temperature, the quantification shows a 50-80% increase of BrdU incorporation in 'late' domains and an equivalent decrease in 'early' domain incorporation in *cdc6-1* cells at 30°C. Data are shown for ChrIV in figure EV1.
2. Show the requirement of Cdc5/Plk1 for Rad52 foci formation in *cdc6-1* cells grown at 30°C, alongside M-CDK (new Fig.2G). These two kinases act synergistically to create the mitotic state and likely contribute to the full phosphorylation of Polα. Cdc5 is now added in our model of fork remodelling at mitotic entry (Fig.6D).
3. Show that we fail to detect DSBs by PFGE analysis of *cdc6-1* cells undergoing MiDAS (new Fig.EV3E), and that MiDAS is independent of Mus81 (new Fig.EV4G). This suggests that fork remodelling by Polα eviction at mitotic entry may not induce DSBs nor require them for initiating mitotic DNA synthesis. Mus81 and Yen1 being required for Rad52 foci disappearance in *cdc6-1* cells, we presume that these nucleases are involved in the resolution of branched DNA structures later in the process.
4. Address the role of Rad52 in MiDAS induced by origin under-licensing: *cdc6-1 rad52Δ* cells being hard to synchronize, we used RAD52-AID2 to deplete Rad52 with OsTIR1/5-Ph-IAA before αF release. This showed unexpectedly that i) Rad52 is required for the metaphase delay of under-licensed *cdc6-1* cells and ii) that MiDAS in *cdc6-1* cells is only partly dependent on Rad52 (30% decrease of MiDAS-positive cells). This suggests that completion of DNA synthesis in mitosis uses both BIR/MMBIR and FoSTeS/MMIR mechanisms, the latter using microhomologies independently of Rad52 (Lee et al., Cell 2007; Payen et al., PLoS Gen2008). It also confirms the role of Rad52 as a sensor of ssDNA along with RPA for the persistent activation of the DNA damage response (Lee et al., Mol Cell Biol 2003). These data are shown in figure EV4 and discussed in the text.
5. Check the rescue of *cdc6-1 polα-cdk25A* lethality by imposing an ectopic metaphase delay, without using nocodazole that activates the SAC and may interfere with the DDR. We made a *cdc6-1 polα-cdk25A MET-CDC20* strain to conditionally deplete (+Met) this APC activator and arrest cells in metaphase for increasing times before scoring for viability on -Met plates. This confirmed and expanded the nocodazole experiment of Fig.5F by showing that *cdc6-1 polα-cdk25A MET-CDC20* loose viability as they exit mitosis (-Met), which is rescued by extending metaphase orthogonally (+Met). These data are shown in figure EV6 and mentioned in the text.

We address below, in blue, all points raised by the referees. The changes to the original manuscript are also indicated in blue in the text.

Referee #1:
This study addresses how cells handle entry into mitosis with incompletely replicated chromosomes and its consequences for genome stability. Using yeast origin licensing mutants, the authors show that under-licensed cells enter mitosis without checkpoint arrest, where M-CDKs phosphorylate Polα, displacing it from the replisome and triggering a DDR that enables BIR-like DNA synthesis.
This is a thoughtful and technically strong study that tackles an important question in chromosome biology. The experiments are carefully designed, and the identification of Polα phosphorylation as a regulatory switch is particularly compelling. I have several suggestions that I believe will help strengthen the manuscript and clarify some of the interpretations.
We are delighted that Referee 1 considers our study thoughtful, technically strong and addressing an important question in chromosome biology.

Specific concerns
The global depletion of replication origins in a synchronized yeast population represents a substantially different scenario from the localized origin scarcity observed at human common fragile sites. While the parallels to cancer biology are interesting, they currently feel somewhat speculative, especially given the noted mechanistic differences (e.g., lack of DSBs and Mus81-Mms4 dependence in yeast MiDAS). It would strengthen the manuscript to more clearly acknowledge these distinctions and to temper the translational implications.
We agree with these comments and will temper the implications of our findings for CFS and cancer biology. Indeed, there are obvious differences between our and those systems, that we acknowledge in the revised ms. Yet, our current work on origin under-licensing in mammalian cells and hPOLA1/A2 phosphorylation gives us confidence that at least some of our conclusions may translate to mechanisms of genome evolution in human and cancer cells.

The nocodazole experiment in cdc6-1 polα-cdk25A cells is elegant and suggests that extending metaphase can rescue viability. However, since nocodazole activates the spindle assembly checkpoint rather than acting as a simple timer, it may have additional effects on DNA damage signalling or replication intermediates. The interpretation would be strengthened either by testing alternative ways of prolonging metaphase or by more explicitly discussing this caveat.

We thank the reviewer for describing this experiment as elegant. As stated above, we now have repeated it using a transient CDC20 arrest, which does not activate the SAC, and found an identical suppression of *cdc6-1 polα-cdk25A* lethality. This confirms that the main reason for co-lethality between origin under-licensing and failure to phosphorylate Polα is due to precocious mitotic exit before cells have time to complete DNA synthesis by alternate routes. It reinforces the role of phosphorylation-induced Polα eviction from the replisome in activating a strong DDR.

The manuscript proposes that BIR-like MiDAS completes replication of a substantial fraction of the genome (~30%), while also reporting an approximately 800-fold increase in GCR rates. It would be helpful to further discuss how cells tolerate such elevated genome instability across divisions, and how these observations can be reconciled.

The GCR assay is sensitive and scores rare events (10e-10/cell division in WT cells). Kolodner & colleagues have scored GCR rates in many yeast mutants, and found equivalent x100-1000 increase GCR in *rad52Δ, mre11Δ* or *rad51Δ* mutants without impairing viability. Although elevated 1000—fold, these GCR events involving the left arm of chromosome V occur only every 10e7 cell division. By extrapolating this region for the entire genome, the GCR rate would remain at 4.10e-5/cell division, sufficiently low to maintain viability, yet high enough to generate new genomic variants. Notably, 10% of *cdc6-1* cells are unable to form colonies at 30°C, underscoring the negative impact of origin under-licencing. This is now commented in the text.

The conclusion that origin firing efficiency is the primary determinant of replication timing is stated strongly but is based mainly on the observed flattening of timing profiles in under-licensed cells. While consistent with this model, other contributing factors such as chromatin organization, transcription, and nuclear architecture are not examined. A more balanced framing of this conclusion would improve the manuscript.

We agree that origin firing is influenced by chromatin context, and now mention it in the text. This notion is exemplified by a recent paper from the Boeke lab (*Lazar-Stefanita et al., Cell Rep 2026*) showing that humanized yeast histones change chromatin structure and flatten replication timing profiles. Similarly, an Orc4 mutant lacking its ACS-recognition motif binds DNA more randomly and also flattens replication profiles (*Lee et al.; Nat Comm 2021*), similar to yeast or mouse *rif1Δ* mutants that advance the firing of late origins. Interestingly, all these mutants affect the rate and/or localization of origin firing, which is consistent with our conclusion that origin firing efficiency is a major contributor of replication timing profiles.

Given the cell-to-cell variability in S-phase progression and mitotic entry, there is a possibility that the BrdU pulse used to detect MiDAS (80-135 min) captures residual late S-phase synthesis. Although morphology-based gating is helpful, it may not fully exclude this possibility. Additional clarification or discussion would be valuable.

We are well aware of this caveat, which is difficult to rule out formally, especially in the QIBC experiments where morphology-based gating is limited. However, to capture MiDAS events, we started the BrdU/EdU pulse at 80 min after G_1 release, well beyond the time of Polα phosphorylation (45 min) and spindle formation (60 min, metaphase) in *cdc6-1* cells. Hence it is more likely that we miss some early MiDAS events using this protocol than capturing late S events. Moreover, we detected MiDAS events (EdU foci in cells with a metaphase spindle, Fig.3A) only in *cdc6-1* cells, not in WT cells, using very short EdU pulses down to 3 min, which limits the possibility of catching late S events. We nevertheless agree that some late S events might be present and contaminate the data in the BrdU-nanoporeSeq experiment. This is now discussed in the revised ms.

The observation that Cdc5 depletion reduces Rad52 foci in cdc6-1 cells is intriguing but not fully integrated into the model. If Cdc5/Plk1 contributes to Polα phosphorylation alongside M-CDK, this could have implications for the model in Figure 6D. It would be helpful to either explore this further or explicitly note it as an open question.

We thank Referee 1 for this comment as we also think that Cdc5/Plk1 should be put in the picture. Pol1 and Pol12 contain several Cdc5/Plk1 consensus phosphorylation sites as well as priming sites (Polo-Box Domain); many are present in our time-resolved phospho-MS data. A paper posted on BioRxiv after our submission finds evidence for Pol12 phosphorylation by Cdc5 *in vitro* (*Shresta et al.; BioRxiv 2026*). We found that depleting Cdc5 suppresses Rad52 foci formation in *cdc6-1* cells, and also have evidence that Cdc5 might phosphorylate Pol1 alongside with M-CDK (see the slight mobility shift of Pol1 in Noc, lost in Noc+IAA *cdc5-aid* in Fig.4A). We have added our data on Rad52 foci after Cdc5 depletion, and now incorporate Cdc5/Plk1 in the model of Polα phosphorylation.

Several strains used in the GCR assays carry the rad5-535 allele, which affects post-replication repair. As BIR intermediates may intersect with this pathway, it would be useful to discuss whether this genetic background could influence the elevated GCR rates observed in cdc6-1 cells.

We agree that the *rad5-535* hypomorphic mutation likely interferes with BIR and affects the nominal GCR rate. We will acknowledge this in the ms. All our GCR strains are congenic (contain the same *rad5* allele present in the original W303 strain) and we score GCR rates as fold-increase compared to WT. It is because Rad5 has been involved in fork regression and template switching that all other key experiments have been carried out in a W303 RAD5 corrected background.

Referee #2:

In this study, the authors investigated the consequences of replication origin under licensing in budding yeast. They show that cdc6-1 or cdt1-21 conditional mutants, when grown under semi-permissive conditions, have a lower number of active origins and a prolonged S-phase resulting in mitotic entry before S-phase completion. Surprising, they do not observe activation of the DNA replication or damage checkpoint (DDC) until mitotic entry. Preventing mitotic entry by over-expression of SWE1 or by use of the cdcd28-as1 allele averted DNA damage and checkpoint activation suggesting forks collapse on mitotic entry. Consistent with fork collapse, cdc6-1 cells exhibit elevated frequencies of chromosome rearrangements and mitotic crossovers/break-induced replication (BIR) at the semi-permissive temperature. In contrast to wild-type cells, cdc6-1 cells exhibit DNA synthesis tracts in mitosis, reminiscent of the aberrant form of DNA synthesis observed at common fragile sites in human cells, referred to as MiDAS. These observations are consistent with fork breakage at under-replicated regions on mitotic entry and subsequent repair by recombination-dependent DNA synthesis. The authors identify the Pol1 and Pol12 subunits of DNA Pol alpha as targets for M-CDK phosphorylation, and subsequently show that mutating these sites to alanines prevents DDC activation, reduces viability and EdU incorporation in mitosis in cdc6-1 cells and leads to premature mitotic exit. Furthermore, they find that Pol alpha phosphorylation by M-CDK in mitotic cells disrupts its interaction with other replisome components, suggesting that replication fork uncoupling is likely responsible for checkpoint activation and initiation of MiDAS.

This is an interesting study that provides a mechanistic understanding for how MiDAS initiates and could potentially explain the higher mutagenic burden of late-replicating regions of the genome. The experimental data are convincing and I have no major concerns with the conclusions.

Minor comments:

Figure 3C: Was MiDAS detected in cdc6-1 rad52 cells? Cells lacking Rad52 generally exhibit stronger HR defects and are defective for the residual BIR detected in rad51 and pol32 cells. Do the rad51 and pol32 mutations reduce viability of cdc6-1 cells grown at the semi-permissive temperature?

As stated above, we have now addressed the role of Rad52 for MiDAS in *cdc6-1* cells using an auxin-inducible degron, and thank the referee for insisting on this since the results were unexpected. We found that Rad52 depletion only decreases the fraction of cells displaying MiDAS without abolishing it, but that it causes *cdc6-1* cells to exit mitosis precociously leading to lethality. Accordingly, we also show that *cdc6-1* is co-lethal with *rad52*Δ and with *pol32*Δ at 30°C, but not *rad51*Δ (new Fig.EV5)

Line 233 and Figure S3D: The Hiraoka et al study reported that ~ 10% of mitotic crossovers/BIR are due to ectopic recombination between repeated sequences so these events are not that rare. Although the PFG does show an aberrant karyotype for 5/11 events tested it is not clear that these are due to translocations involving chromosome III because both copies of chromosome III appear to be present in some lanes labeled as TL (#4, #6).

Thanks for this insightful comment. Indeed, lanes 4 & 6 (perhaps 10 also) do not show a loss of one copy of ChrIII, but contain a new chromosome of aberrant size. These clones might have arisen from multiple recombination events, a crossing-over at ChrIII and an ectopic recombination at another chromosome (note the lowered intensity of ChrXIII in lane #4). We will precise this in the text.

Line 304: The authors suggest that Pol alpha phosphorylation does not promote MiDAS directly and is mainly to trigger the checkpoint to arrest cell division to allow time for MiDAS to occur. Studies of BIR at endonuclease-induced double-strand breaks (DSBs) have shown an essential role for Pol alpha-primase to complete BIR (Lydeard et al., 2007; Donnianni et al., 2019), presumably to prime synthesis of the second strand. It would be of interest to determine whether the pol alpha-cdk25A mutant is proficient for BIR at DSBs.

We agree that it would be interesting to test if Polα phosphorylation is required for endonuclease-induced DSB repair by BIR, but we feel that this out of the scope of this ms since we do not use induced DSBs in our system. Although we favour a main role for Polα phosphorylation in eviction from CMG and checkpoint activation, we cannot rule out a second role on BIR itself, as shown in the cited papers. In fact, we have preliminary evidence that phosphorylation might redirect Polα from CMG to CST in late G_2, in order to perform second strand synthesis of telomeres (C-strand). It is possible and will be interesting to test in the future if second strand BIR synthesis is performed by phospho-Polα bound to CST.

Line 472 and Figure 6D: Presumably, the initiation of MiDAS would require fork breakage to generate an end for strand invasion. The authors state that yeast cells perform MiDAS independently of the Mus81-Mms4 nuclease. There is no reference for this statement or data addressing this point in the manuscript. Yen1 would likely not be activated at the time MiDAS initiates, but Slx1 or Rad1 might be candidated for cleavage of uncoupled forks. I'm not suggesting that the authors test these candidates but there should be some discussion of this point.

We stated (line 472) that DDR activation (using Rad52 foci as a proxy), not MiDAS, is independent of Mus81-Mms4. We have now tested directly the role of Mus81 for MiDAS and found that it is not required (new Fig.EV4G). We also show that we failed to detect broken chromosomes in *cdc6-1* cells entering mitosis (Fig.EV3E). Although it remains possible that cleaved forks remain in the well of the PFGE gel, our data rather suggest that MiDAS in under-licensing yeast does not cause or require fork breakage, similar to what has been shown for FoSTeS, segmental duplications

or in the RTS1 fork stalling system in fission yeast. However, this does not rule out a role of branched-structure nucleases and Rad1 later in the resolution of template switching intermediates, as we found that i) Rad52 foci have a longer lifetime in *cdc6-1 mus81 yen1* cells and ii) that Mus81, Yen1 and Rad1 each contribute to the viability of *cdc6-1* cells at 30°C. This is now commented in the revised version.

Referee #3:
This paper examines what happens in budding yeast when the total number of replication origins is reduced by partially inhibiting the replication licensing machinery. As has been shown in animal cells, this causes a significant increase in mitotic DNA synthesis. Interestingly, the paper goes on to show that this causes hyperphosphorylation of DNA polymerase alpha and its removal from chromatin, with the mitotic DNA synthesis being dependent on genes involved in break-induced replication. This provides interesting new detail about mitotic DNA synthesis, and suggests conservation of mechanism across the eukaryotic kingdom. The work merits publication.

Overall the data are strong and support the central hypothesis. However, there are two areas where I thought the authors have pushed the interpretation of the data a little too far; neither of these cases however undermine the central thesis as outlined above. My criticisms can be addressed by either softening the wording or addition of a few simple controls.
We thank this referee for his analysis and support.

1. In two places, the authors comment on how they think that under-licensing caused by the cdc6-1 mutation affects the replication programme. They draw conclusions based on a relatively superficial analysis of the DNAscent data in Figure 1 and S1. Line 131 "Unexpectedly, the late/inefficient origins fired more frequently in cdc6-1 cells at 30°C." Line 351: "An unexpected finding is that origin under-licensing obliterates the stereotypical replication timing program shared by most cells in the population (Figure 1F)." Without more analysis of the DNAscent data I don't think these statements are justified, just because the peaks have become more flattened. At most, it may be possible that the difference between early and late origins is reduced and they now fire more equivalently. I think it is enough to simply says that the replication programme is clearly changed from normal.
BrdU nanopore sequencing is performed on native DNA, is quantitative and done on cells arrested in early S with HU. We have now measured the area under the curve (AUC) for normally early and late-replicating domains along yeast chromosomes (Fig.EV1E). This shows that so-called early and late origins fire less or more frequently, respectively, in early S or *cdc6-1* cells arrested in early S. This is consistent with the idea that reduced licensing in *cdc6-1* or *cdt1-1* cells affects all origins stochastically in the cell population, redistributing origin firing and limiting initiation factors at non-preferred sites including normally late/inefficient origins, which causes the flattening of the replication profile. This is in line with what Referee #3 says: 'early and late origins fire more equivalently'. We take the comment and have improved our message in the text.

2. In Figure 2 the authors use a couple of approaches to inhibit CDC28 in order to conclude that mitotic entry is responsible for RAD53 phosphorylation. But since CDC28 is also responsible for S phase entry and origin firing as well as mitotic entry, I don't find this data convincing. I would have thought that SWE1 overexpression would inhibit the S phase function of CDC28 as well as its mitotic function. 1-NMPP1 inhibition of cdc28-as1 is better evidence, because 1-NMPP1 added after alpha factor release should allow the majority of origins to fire. If the authors want to maintain this argument, DNA content analysis by flow cytometry should at the minimum be shown. Even so, a better approach would be use of a mutant that really should not itself interfere with DNA replication (CDC5/Polo or MIH1/CDC25?).
This key experiment addressing the role of M-CDK in DDR activation in under-licensed cells has been performed in three different ways: Swe1 OE, cdc28-as inhibition by 1-NMPP1 after replication, and M-cyclins deletion/depletion (in *clb1 clb3 clb4 GAL-CLB2* strain). They all confirmed the requirement of M-CDK for checkpoint activation in *cdc6-1* cells. It has been clearly shown that Swe1 does not inhibit S-phase cyclins (Clb5,6-Cdk1) but targets preferentially mitotic cyclins (Clb2 > Clb3,4)-associated Cdk1 *in vivo* (Hu & Aparicio, PNAS 2005; Liu & Wang, MBoC 2006; Hu et al., Genetics 2008). Accordingly, DNA synthesis is unaffected by Swe1 over expression from αF release in our experiment shown in Fig 2D, 2E and 6C. Moreover, mutating CDK consensus sites in Pol1 & Pol12 suppressed DDR activation in mitosis of *cdc6-1* cells. We have tested Cdc5 depletion, which also suppresses Rad52 foci formation in *cdc6-1* cells (new Fig.2G). Deletion of *MIH1* does not block mitotic entry, it only delays it (Pal et al., JCB 2008). Altogether, we feel that we provide irrefutable evidence that M-CDK activity, along with Cdc5/Plk1, is required for Polα phosphorylation, eviction from forks and activation of the DDR.

Minor points:
3. I presume controls were done for Fig 2E that CDC20 and CDC15 mutations did indeed block anaphase and cell division?
Absolutely. These cells contain mCherry-tagged tubulin that allowed us to confirm metaphase and telophase arrests after Cdc20 or Cdc15 inactivation, respectively. We will mention this in the text, and provide images in data source deposition.

4. Figure 3C: why are ~10% of cells scored as Mitotic EdU+ even in the absence of EdU? This seems like a very high false positive rate.

We developed QIBC for yeast, which are small objects with high autofluorescence, explaining in part the rather high false-positive EdU score. We since have optimized the filtering pipeline, which increased the MiDAS signal-to-noise ratio, yet without reducing the near 10% false-positive rate (rarely provided in other studies). These new analyses confirm the requirement of Pol32 for MiDAS, and the contribution of Mph1, Sgs1 and Srs2 (Fig.EV4F).

5. Line 401: "Our study uncovers the long-sought role of Pola phosphorylation,". They show one role, but there could be others.
Of course, Polα phosphorylation could have other roles, and we already have evidence for that. We will change our wording.

Dr. Etienne Schwob
Institut de Génétique Moléculaire (IGMM)
CNRS, Univ Montpellier
1919, route de Mende
Montpellier 34293
France

7th Sep 2026

Re: EMBOJ-2026-124453R
Switch from canonical to recombination-dependent DNA synthesis by M-CDK phosphorylation of DNA Pola

Dear Etienne,

Thank you for submitting your revised manuscript on CDK phosphorylation of Pol alpha to The EMBO Journal. It has now once more been seen by two of the original referees (see comments below), who were both fully satisfied with the revisions. We shall therefore be happy to accept the manuscript for publication, following incorporation of a number of remaining editorial issues as follows:

- Please carefully go through the reference list and make sure that all references have their complete citation information - including year, journal name & volume, and page/elocator numbers - some of this information seems to be missing in cases.

- In the Data Availability section, please spell out (rather than embed) general access hyperlinks to each of the utilized databases (i.e., ENA, PRIDE, BioStudies), followed by the respective access codes. Please remove any referee tokens, and initiate public release of the deposited data at this stage to ensure their open availability at the time of publication.

- As we have switched from a free-text author contribution statement towards a more formal statement based on Contributor Role Taxonomy (CRediT) terms, please remove the present Author Contribution section and instead specify each author's contribution(s) directly in the Author Information page of our submission system during upload of the final manuscript. See https://casrai.org/credit/ for more information.

- Please rename the Conflict of Interest section into "Disclosure and Competing Interests Statement", in accordance with our updated Guide to Authors (https://www.embopress.org/competing-interests), and place it AFTER the Acknowledgements section.

- In the Appendix, please make sure to label each Appendix Figure not just in the ToC, but also on its respective first page, with Figure name and Figure legend underneath. Please remove the mention of Appendix material from the (end of) the main text. Finally, there seems to be a citation of "Appendix Text" in the main text, which will need to be corrected/removed

- Please remove the mention of EV Tables from the main text, and make sure to include each EV Table legend within each Table file itself. In Table EV2, please make sure that the references to other items all follow the same conventions as in-text references (e.g., Appendix Figure S1 rather than Appendix S1).

- Please remove mention/legends of EV Movies from the main text, their legends should only be contained within each respective Movie ZIP file.

- Please provide suggestions for a short 'blurb' text prefacing and summing up the conceptual aspect of the study in two sentences (max. 250 characters), followed by 3-5 one-sentence 'bullet points' with brief factual statements of key results of the paper; they will form the basis of an editor-written 'Synopsis' accompanying the online version of the article. Please also upload a synopsis image, which can be used as a "visual title" for the synopsis section of your paper. The image should be in JPG format and exactly 550 pixels wide and 300 pixels high, and please make sure that it remains clear enough even in these modest dimensions.

- Finally, during routine pre-acceptance checks, our data editors have raised the following queries regarding figures, data, and legends; I would appreciate if you briefly answered to them in the cover letter of your final submission, and made the requested text modifications with changes/additions highlighted via the "Track changes" option, to facilitate our final checking:
1) Please define the annotated p values ****/***/**/* as well as provide the exact p-values for the same in the legend of figure 1C, EV4 E as appropriate.
2) Please indicate the exact p values in the legends of figures 1C, 3B, C; 5C
3) Please indicate the statistical test used for data analysis in the legends of figures 6A, EV4 E
4) Please provide information related to n in the legends of figures EV1 E, EV4 E

5) Please define the error bars in the legends of figures 3C, 5C, EV1 E, EV4 E
6) Please define the dashed lines in the legend of figure 1C.
7) Please define the dashed circular borders in the legend of figure 3B.

I am returning the manuscript to you for a final round of minor revision, hoping you will be able to re-upload the modified final files during the course of this week. Once we will have received them, we should be ready to swiftly proceed with formal acceptance and production of the manuscript.

With kind regards,

Hartmut

Hartmut Vodermaier, PhD
Senior Editor, The EMBO Journal
h.vodermaier@embojournal.org

*** PLEASE NOTE: All revised manuscript are subject to initial checks for completeness and adherence to our formatting guidelines. Revisions may be returned to the authors and delayed in their editorial re-evaluation if they fail to comply to the following requirements. As a first step please read our guidelines for revised submissions:
https://link.springer.com/journal/44318/submission-guidelines#cms-Revised-submissions

1) Every manuscript requires a Data Availability section (even if only stating that no deposited datasets are included). Primary datasets or computer code produced in the current study have to be deposited in appropriate public repositories prior to resubmission, and reviewer access details provided in case that public access is not yet allowed.

2) Each figure legend must specify
- size of the scale bars that are mandatory for all micrograph panels
- the statistical test used to generate error bars and P-values
- the type error bars (e.g., S.E.M., S.D.)
- the number (n) and nature (biological or technical replicate) of independent experiments underlying each data point
- Figures may not include error bars for experiments with n<3; scatter plots showing individual data points should be used instead.

3) Revised manuscript text (including main tables, and figure legends for main and EV figures) has to be submitted as editable text file (e.g., .docx format). We encourage highlighting of changes (e.g., via text color) for the referees' reference.

4) Each main and each Expanded View (EV) figure should be uploaded as individual production-quality files (preferably in .eps, .tif, .jpg formats). For suggestions on figure preparation/layout, please refer to our Figure Preparation Guidelines:
https://media.springernature.com/original/springer-cms/rest/v1/content/27825798/data/v1

5) Point-by-point response letters should include the original referee comments in full together with your detailed responses to them (and to specific editor requests if applicable), and also be uploaded as editable (e.g., .docx) text files.

6) Please complete our Author Checklist, and make sure that information entered into the checklist is also reflected in the manuscript; the checklist will be available to readers as part of the Review Process File.

7) All authors listed as (co-)corresponding need to deposit, in their respective author profiles in our submission system, a unique ORCiD identifier linked to their name. Please see our Guide to Authors for detailed instructions.

8) Please note that supplementary information at EMBO Press has been superseded by the 'Expanded View' for inclusion of additional figures, tables, movies or datasets; with up to five EV Figures being typeset and directly accessible in the HTML version of the article.

9) To facilitate reproducibilty and cross-laboratory adoption of methodologies, please structure the Materials & Methods section as outlined in our guide to authors, including a completed Reagents and Tools Table.

10) Digital image enhancement is acceptable practice, as long as it accurately represents the original data and conforms to community standards. If a figure has been subjected to significant electronic manipulation, this must be clearly noted in the figure legend and/or the 'Materials and Methods' section. The editors reserve the right to request original versions of figures and the original images that were used to assemble the figure. Finally, we generally encourage uploading of numerical as well as gel/blot image source data.

At EMBO Press we ask authors to provide source data for the main manuscript figures. You will receive a separate email with instructions for providing source data with your revised manuscript, including how to upload and organize the files.

In the interest of ensuring the conceptual advance provided by the work, we recommend submitting a revision within 3 months (6th Dec 2026). Please discuss the revision progress ahead of this time with the editor if you require more time to complete the revisions. Use the link below to submit your revision:

Link Not Available

Referee #2:

The authors have addressed my concerns/comments in the revised manuscript.

Referee #3:

The authors have addressed all my points and I think the manuscript is suitable for publication.

EMBO Press Author Checklist

Corresponding Author Name: Etienne SCHWOB
Journal Submitted to: The EMBO Journal
Manuscript Number: 2026-124453R

USEFUL LINKS FOR COMPLETING THIS FORM
The EMBO Journal - Author Guidelines
EMBO Reports - Author Guidelines
Molecular Systems Biology - Author Guidelines
EMBO Molecular Medicine - Author Guidelines

Reporting Checklist for Life Science Articles (updated January
This checklist is adapted from Materials Design Analysis Reporting (MDAR) Checklist for Authors. MDAR establishes a minimum set of requirements in transparent reporting in the life sciences (see Statement of Task: 10.31222/osf.io/9sm4x). Please follow the journal's guidelines in preparing your
Please note that a copy of this checklist will be published alongside your article.

Abridged guidelines for figures
1. Data
The data shown in figures should satisfy the following conditions:
- the data were obtained and processed according to the field's best practice and are presented to reflect the results of the experiments in an accurate and unbiased manner.
- ideally, figure panels should include only measurements that are directly comparable to each other and obtained with the same assay.
- plots include clearly labeled error bars for independent experiments and sample sizes. Unless justified, error bars should not be shown for technical
- if n<5, the individual data points from each experiment should be plotted. Any statistical test employed should be justified.
- Source Data should be included to report the data underlying figures according to the guidelines set out in the authorship guidelines on Data

2. Captions
Each figure caption should contain the following information, for each panel where they are relevant:
- a specification of the experimental system investigated (eg cell line, species name).
- the assay(s) and method(s) used to carry out the reported observations and measurements.
- an explicit mention of the biological and chemical entity(ies) that are being measured.
- an explicit mention of the biological and chemical entity(ies) that are altered/varied/perturbed in a controlled manner.
- the exact sample size (n) for each experimental group/condition, given as a number, not a range;
- a description of the sample collection allowing the reader to understand whether the samples represent technical or biological replicates (including how many animals, litters, cultures, etc.).
- a statement of how many times the experiment shown was independently replicated in the laboratory.
- definitions of statistical methods and measures:
  - common tests, such as t-test (please specify whether paired vs. unpaired), simple χ2 tests, Wilcoxon and Mann-Whitney tests, can be unambiguously identified by name only, but more complex techniques should be described in the methods section;
  - are tests one-sided or two-sided?
  - are there adjustments for multiple comparisons?
  - exact statistical test results, e.g., P values = x but not P values < x;
  - definition of 'center values' as median or average;
  - definition of error bars as s.d. or s.e.m.

Please complete ALL of the questions below.
Select "Not Applicable" only when the requested information is not relevant for your study.

Materials

Newly Created Materials	Information included in the manuscript?	In which section is the information available? (Reagents and Tools Table, Materials and Methods, Figures, Data Availability Section)
New materials and reagents need to be available; do any restrictions apply?	Yes	Table S2: Yeast strains

Antibodies	Information included in the manuscript?	In which section is the information available? (Reagents and Tools Table, Materials and Methods, Figures, Data Availability Section)
For **antibodies** provide the following information:<br>- Commercial antibodies: RRID (if possible) or supplier name, catalogue number and or/clone number<br>- Non-commercial: RRID or citation	Yes	Materials and Methods

DNA and RNA sequences	Information included in the manuscript?	In which section is the information available? (Reagents and Tools Table, Materials and Methods, Figures, Data Availability Section)
**Short novel DNA or RNA including primers, probes**: provide the sequences.	Not Applicable	

Cell materials	Information included in the manuscript?	In which section is the information available? (Reagents and Tools Table, Materials and Methods, Figures, Data Availability Section)
**Cell lines:** Provide species information, strain. Provide accession number in repository **OR** supplier name, catalog number, clone number, and/**OR** RRID.	Yes	Table S2: Yeast strains
**Primary cultures:** Provide species, strain, sex of origin, genetic modification status.	Not Applicable	
Report if the cell lines were recently **authenticated** (e.g., by STR profiling) and tested for mycoplasma contamination.	Not Applicable	

Experimental animals	Information included in the manuscript?	In which section is the information available? (Reagents and Tools Table, Materials and Methods, Figures, Data Availability Section)
**Laboratory animals or Model organisms:** Provide species, strain, sex, age, genetic modification status. Provide accession number in repository **OR** supplier name, catalog number, clone number, **OR** RRID.	Not Applicable	
**Animal observed in or captured from the field:** Provide species, sex, and age where possible.	Not Applicable	
Please detail **housing and husbandry conditions**.	Not Applicable	

Plants and microbes	Information included in the manuscript?	In which section is the information available? (Reagents and Tools Table, Materials and Methods, Figures, Data Availability Section)
**Plants:** provide species and strain, ecotype and cultivar where relevant, unique accession number if available, and source (including location for collected wild specimens).	Not Applicable	
**Microbes:** provide species and strain, unique accession number if available, and source.	Yes	Table S2: Yeast strains

Human research participants	Information included in the manuscript?	In which section is the information available? (Reagents and Tools Table, Materials and Methods, Figures, Data Availability Section)
If collected and within the bounds of privacy constraints report on age, sex and gender or ethnicity for all study participants.	Not Applicable	

Core facilities	Information included in the manuscript?	In which section is the information available? (Reagents and Tools Table, Materials and Methods, Figures, Data Availability Section)
If your work benefited from core facilities, was their service mentioned in the acknowledgments section?	Yes	

Design

Study protocol	Information included in the manuscript?	In which section is the information available? (Reagents and Tools Table, Materials and Methods, Figures, Data Availability Section)
If study protocol has been **pre-registered, provide DOI in the manuscript**. For clinical trials, provide the trial registration number **OR** cite DOI.	Not Applicable	
Report the **clinical trial registration number** (at ClinicalTrials.gov or equivalent), where applicable.	Not Applicable	

Laboratory protocol	Information included in the manuscript?	In which section is the information available? (Reagents and Tools Table, Materials and Methods, Figures, Data Availability Section)
Provide DOI OR other citation details if **external detailed step-by-step protocols** are available.	Yes	Materials and Methods. References

Experimental study design and statistics	Information included in the manuscript?	In which section is the information available? (Reagents and Tools Table, Materials and Methods, Figures, Data Availability Section)
Include a statement about **sample size** estimate even if no statistical methods were used.	Yes	Figures
Were any steps taken to minimize the effects of subjective bias when allocating animals/samples to treatment (e.g. **randomization procedure**)? If yes, have they been described?	Yes	Sample blinding and two-people scoring
Include a statement about **blinding** even if no blinding was done.	Yes	Materials and Methods.
Describe **inclusion/exclusion criteria** if samples or animals were excluded from the analysis. Were the criteria pre-established?<br><br>If sample or data points were omitted from analysis, report if this was due to attrition or intentional exclusion and provide justification.	Not Applicable	
For every figure, are **statistical tests** justified as appropriate? Do the data meet the assumptions of the tests (e.g., normal distribution)? Describe any methods used to assess it. Is there an estimate of variation within each group of data? Is the variance similar between the groups that are being statistically compared?	Yes	Figures

Sample definition and in-laboratory replication	Information included in the manuscript?	In which section is the information available? (Reagents and Tools Table, Materials and Methods, Figures, Data Availability Section)
In the figure legends: state number of times the experiment was **replicated** in laboratory.	Yes	Figures
In the figure legends: define whether data describe **technical or biological replicates**.	Yes	Figures

Ethics

Ethics	Information included in the manuscript?	In which section is the information available? (Reagents and Tools Table, Materials and Methods, Figures, Data Availability Section)
Studies involving **human participants**: State details of **authority granting ethics approval** (IRB or equivalent committee(s), provide reference number for approval.	Not Applicable	
Studies involving **human participants**: Include a statement confirming that **informed consent** was obtained from all subjects and that the experiments conformed to the principles set out in the WMA Declaration of Helsinki and the Department of Health and Human Services Belmont Report.	Not Applicable	
Studies involving **human participants:** For publication of **patient photos**, include a statement confirming that consent to publish was obtained.	Not Applicable	
Studies involving experimental **animals**: State details of **authority granting ethics approval** (IRB or equivalent committee(s), provide reference number for approval. Include a statement of compliance with ethical regulations.	Not Applicable	
Studies involving **specimen and field samples:** State if relevant **permits** obtained, provide details of authority approving study; if none were required, explain why.	Not Applicable	

Dual Use Research of Concern (DURC)	Information included in the manuscript?	In which section is the information available? (Reagents and Tools Table, Materials and Methods, Figures, Data Availability Section)
Could your study fall under dual use research restrictions? Please check biosecurity documents and list of **select agents and toxins** (CDC): https://www.selectagents.gov/sat/list.htm	Not Applicable	
If you used a select agent, is the security level of the lab appropriate and reported in the manuscript?	Not Applicable	
If a study is subject to dual use research of concern regulations, is the name of the **authority granting approval and reference number** for the regulatory approval provided in the manuscript?	Not Applicable	

Reporting

The MDAR framework recommends adoption of discipline-specific guidelines, established and endorsed through community initiatives. Journals have their own policy about requiring specific guidelines and recommendations to complement MDAR.

Adherence to community standards	Information included in the manuscript?	In which section is the information available? (Reagents and Tools Table, Materials and Methods, Figures, Data Availability Section)
State if relevant guidelines or checklists (e.g., **ICMJE, MIBBI, ARRIVE, PRISMA**) have been followed or provided.	Yes	Authors contribution. Data availability
For **tumor marker prognostic studies,** we recommend that you follow the **REMARK** reporting guidelines (see link list at top right). See author guidelines, under 'Reporting Guidelines'. Please confirm you have followed these guidelines.	Not Applicable	
For **phase II and III randomized controlled trials**, please refer to the **CONSORT** flow diagram (see link list at top right) and submit the CONSORT checklist (see link list at top right) with your submission. See author guidelines, under 'Reporting Guidelines'. Please confirm you have submitted this list.	Not Applicable	

Data Availability

Data availability	Information included in the manuscript?	In which section is the information available? (Reagents and Tools Table, Materials and Methods, Figures, Data Availability Section)
Have **primary datasets** been deposited according to the journal's guidelines (see 'Data Deposition' section) and the respective accession numbers provided in the Data Availability Section?	Yes	Data availability.
Were **human clinical and genomic datasets** deposited in a public access-controlled repository in accordance to ethical obligations to the patients and to the applicable consent agreement?	Not Applicable	
Are **computational models** that are central and integral to a study available without restrictions in a machine-readable form? Were the relevant accession numbers or links provided?	Yes	Code availability
If publicly available data were reused, provide the respective **data citations in the reference list**.	Not Applicable	
