# Supplementary material for: Switch from canonical to recombination-dependent DNA synthesis by M-CDK phosphorylation of DNA Polα
Source: EMBO J. 2026 Oct 10;45(21):7621–46. doi: 10.1038/s44318-026-00919-5 (PMC13728163; doi:10.1038/s44318-026-00919-5)
Supplement: Supplementary file 6 [file 44318_2026_919_MOESM10_ESM.zip › Figure 4/4D/Figure 4D.pdf]

25ºC

WT
cdc6-1
cdc6-1 polα-cdk25A
polα-cdk25A

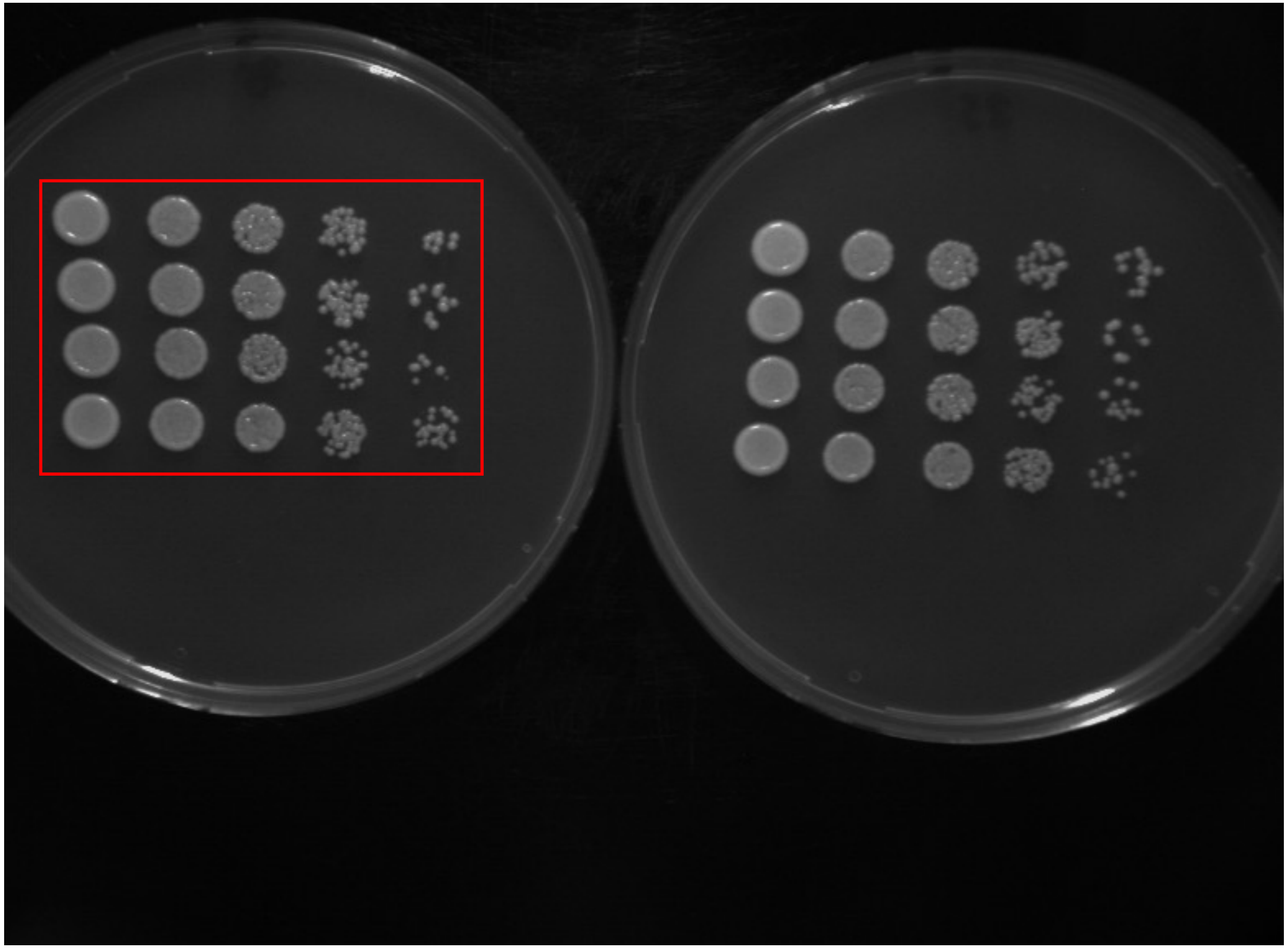

30ºC

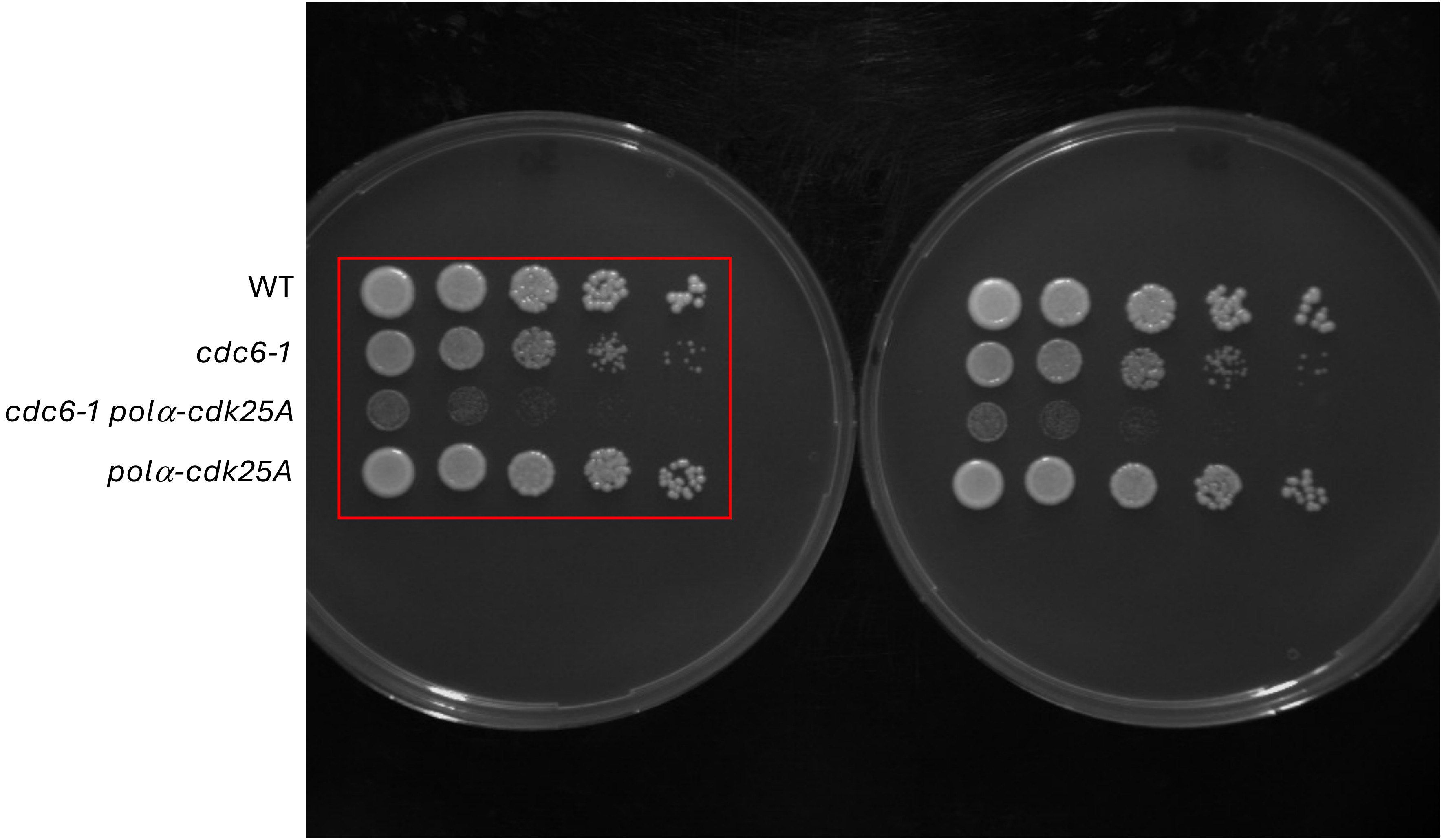

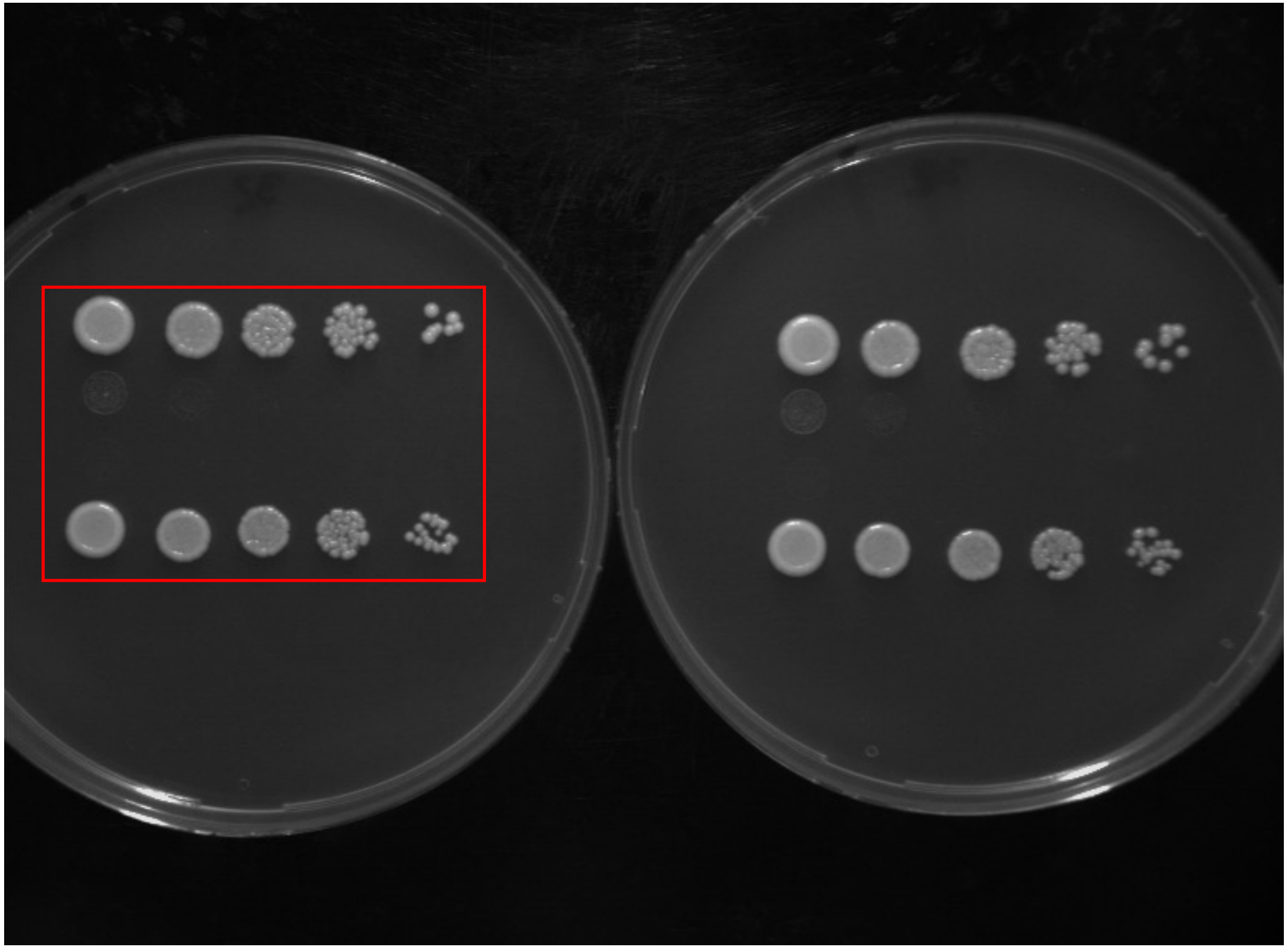
32ºC
WT
cdc6-1
cdc6-1 polα-cdk25A
polα-cdk25A
