# Supplementary material for: Switch from canonical to recombination-dependent DNA synthesis by M-CDK phosphorylation of DNA Polα
Source: EMBO J. 2026 Oct 10;45(21):7621–46. doi: 10.1038/s44318-026-00919-5 (PMC13728163; doi:10.1038/s44318-026-00919-5)

2020 11 28

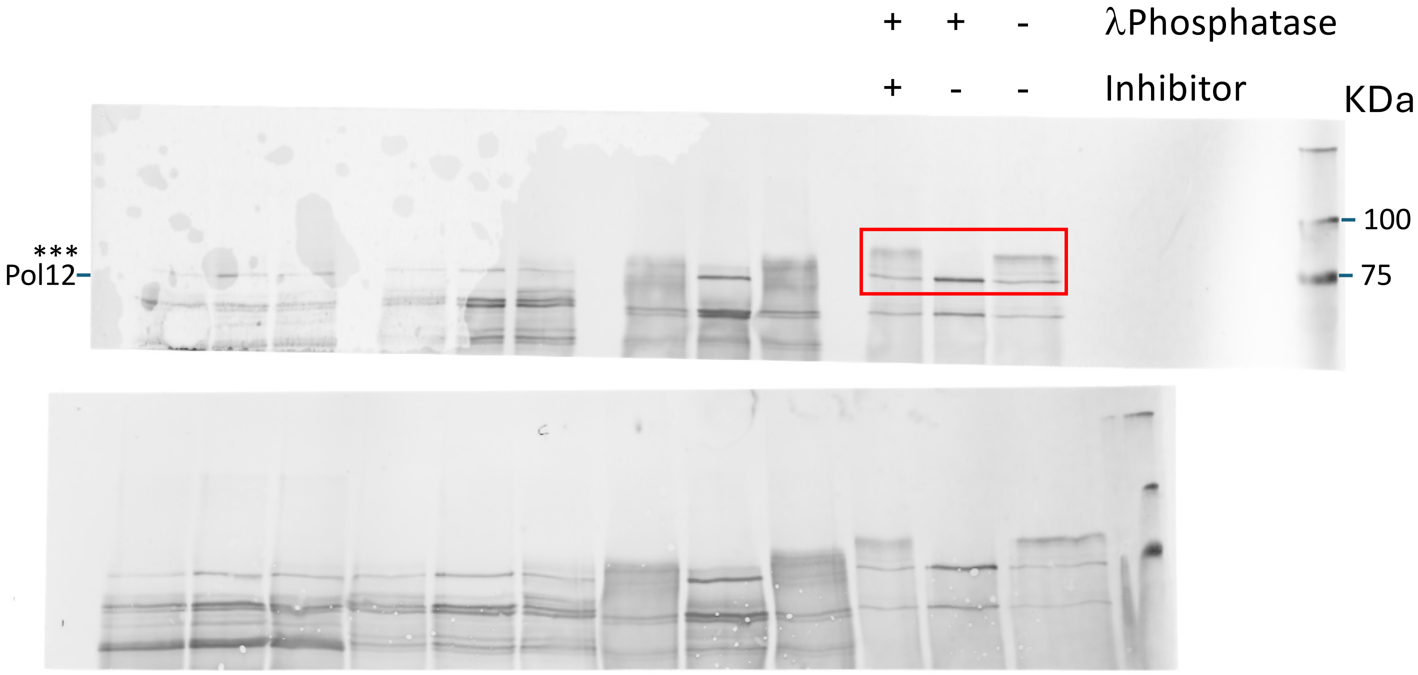

Supplement: Supplementary file 6 [file 44318_2026_919_MOESM10_ESM.zip › Figure 4/4B/phosphatase assay.pdf]
