# Supplementary material for: Switch from canonical to recombination-dependent DNA synthesis by M-CDK phosphorylation of DNA Polα
Source: EMBO J. 2026 Oct 10;45(21):7621–46. doi: 10.1038/s44318-026-00919-5 (PMC13728163; doi:10.1038/s44318-026-00919-5)
Supplement: Supplementary file 7 [file 44318_2026_919_MOESM11_ESM.zip › Figure 5/5E/fig5E.pdf]

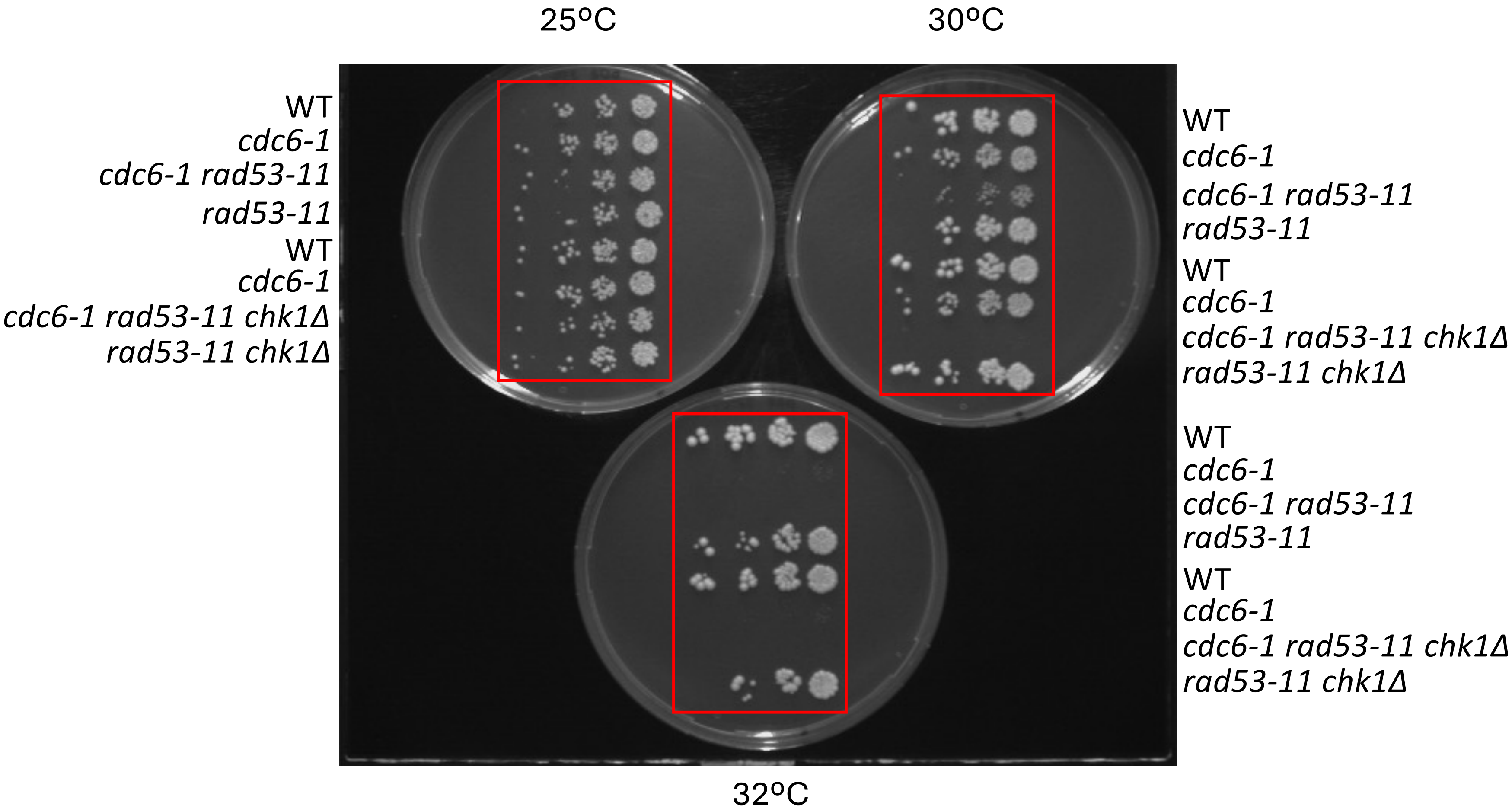
25ºC
30ºC
32ºC
WT
cdc6-1
cdc6-1 rad53-11
rad53-11
WT
cdc6-1
cdc6-1 rad53-11 chk1Δ
rad53-11 chk1Δ
WT
cdc6-1
cdc6-1 rad53-11
rad53-11
WT
cdc6-1
cdc6-1 rad53-11 chk1Δ
rad53-11 chk1Δ
WT
cdc6-1
cdc6-1 rad53-11
rad53-11
WT
cdc6-1
cdc6-1 rad53-11 chk1Δ
rad53-11 chk1Δ
