# Supplementary material for: Switch from canonical to recombination-dependent DNA synthesis by M-CDK phosphorylation of DNA Polα
Source: EMBO J. 2026 Oct 10;45(21):7621–46. doi: 10.1038/s44318-026-00919-5 (PMC13728163; doi:10.1038/s44318-026-00919-5)

Strain E3692: *GALp-SWE1*

Protein: Pol12

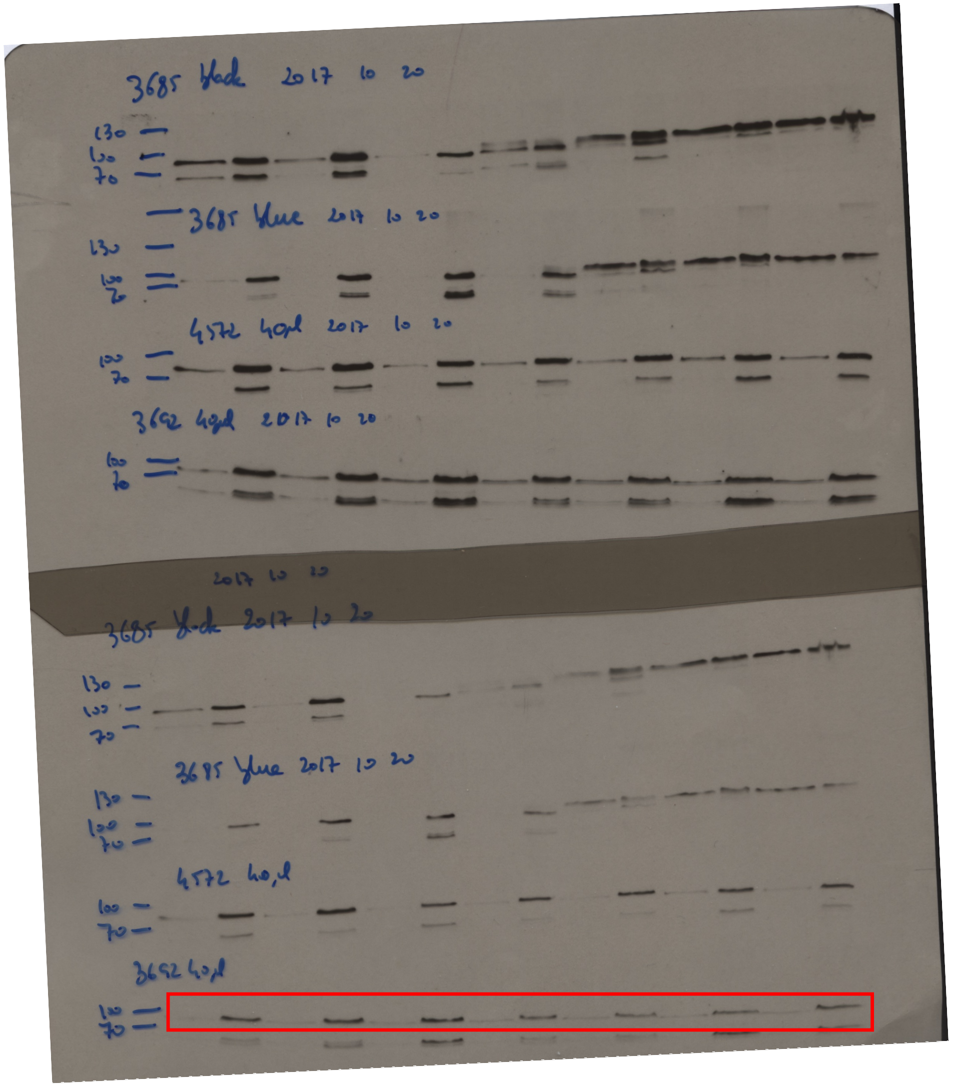

Strain E3692: *GALp-SWE1*

Protein: Orc6

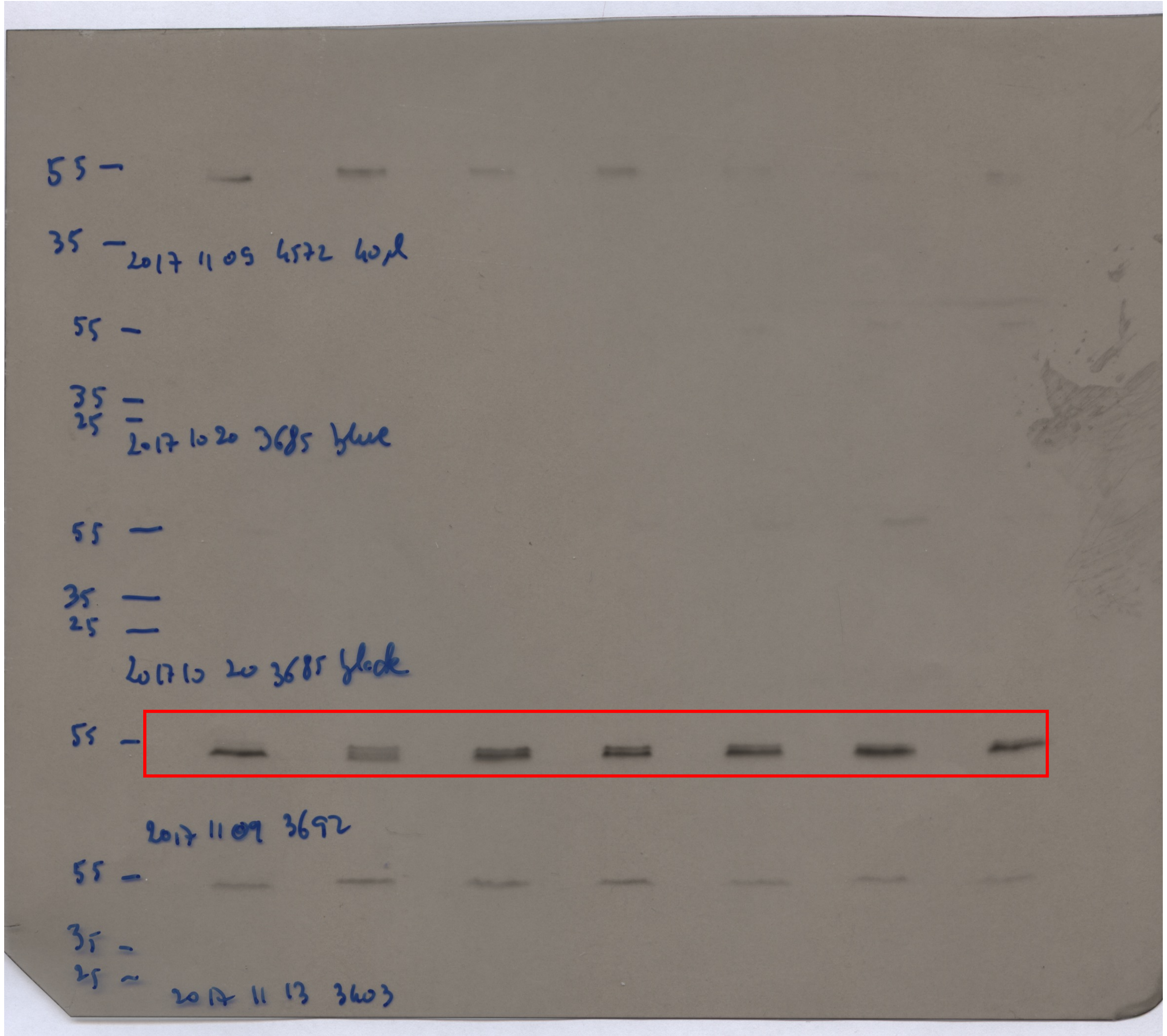

Strain E3692: *GALp-SWE1*

Protein: Pgk1

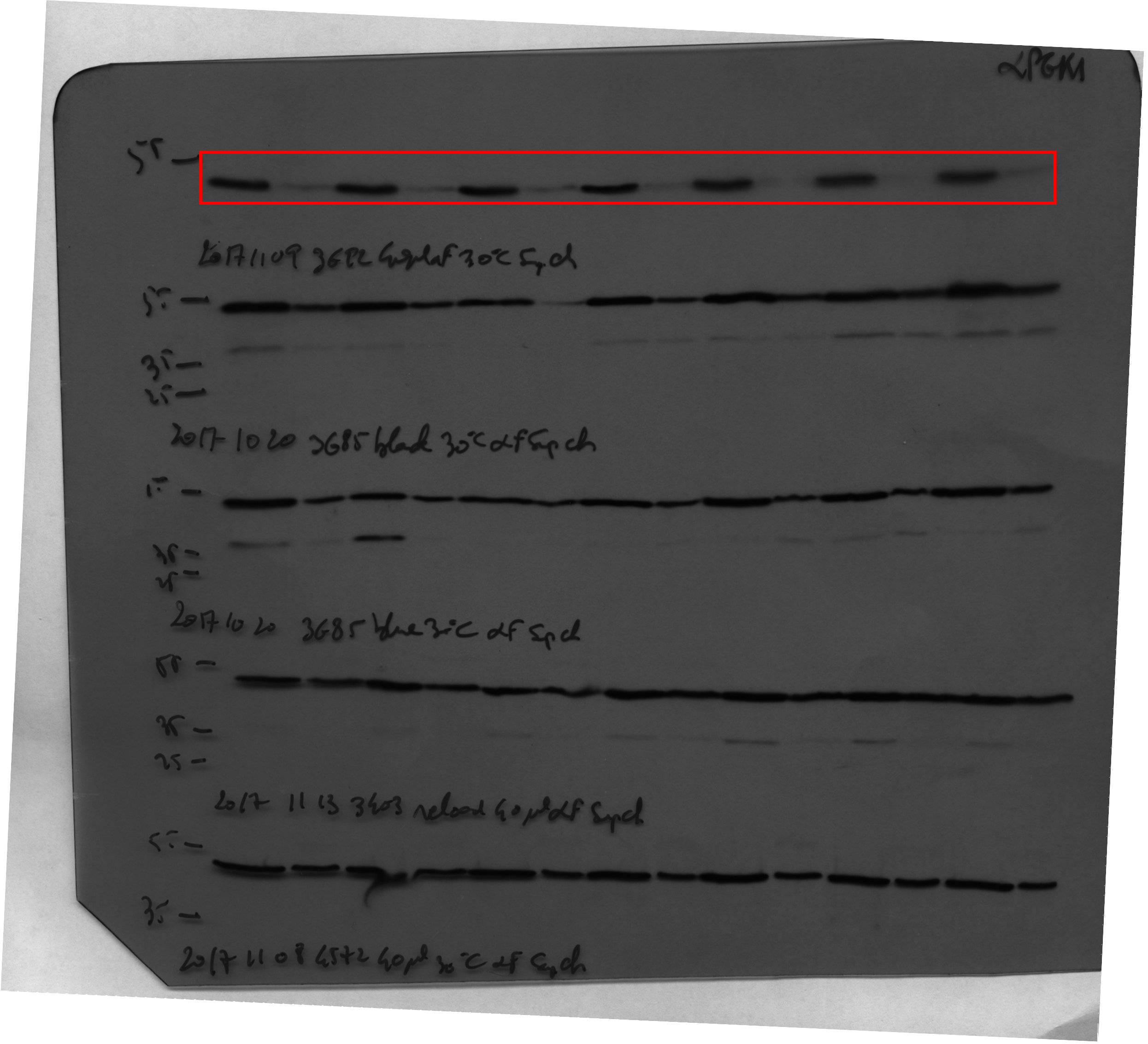

Supplement: Supplementary file 8 [file 44318_2026_919_MOESM12_ESM.zip › Figure 6/6C/6C WB GAL_SWE1.pdf]
