# Supplementary material for: Switch from canonical to recombination-dependent DNA synthesis by M-CDK phosphorylation of DNA Polα
Source: EMBO J. 2026 Oct 10;45(21):7621–46. doi: 10.1038/s44318-026-00919-5 (PMC13728163; doi:10.1038/s44318-026-00919-5)
Supplement: Supplementary file 9 [file 44318_2026_919_MOESM5_ESM.zip › Movie EV1 legend.docx]

**Movie EV1: Rad52 foci formation and cell cycle progression *cdc6-1* cells at 30°C**.

*cdc6-1* cells were imaged every 2 min for 12h at 30°C on a spinning disk microscope to visualize Rad52 foci (green) alongside mitotic spindles (mCherry-TUB1, red). Rad52 foci appear shortly before spindle formation and disappear just before anaphase.
