# Supplementary material for: Switch from canonical to recombination-dependent DNA synthesis by M-CDK phosphorylation of DNA Polα
Source: EMBO J. 2026 Oct 10;45(21):7621–46. doi: 10.1038/s44318-026-00919-5 (PMC13728163; doi:10.1038/s44318-026-00919-5)
Supplement: Supplementary file 10 [file 44318_2026_919_MOESM6_ESM.zip › Movie EV2 legend.docx]

**Movie EV2: Rad52 foci formation, metaphase arrest and cell death in *cdc6-1* cells at 34°C.**

*cdc6-1* cells were imaged every 2 min for 12h at 34°C on a spinning disk microscope to visualize Rad52 foci (green) alongside mitotic spindles (mCherry-Tub1, red). Rad52 foci appear shortly before spindle formation, but persist in enlarging cells blocked in metaphase, until death.
