# Supplementary material for: Switch from canonical to recombination-dependent DNA synthesis by M-CDK phosphorylation of DNA Polα
Source: EMBO J. 2026 Oct 10;45(21):7621–46. doi: 10.1038/s44318-026-00919-5 (PMC13728163; doi:10.1038/s44318-026-00919-5)
Supplement: Supplementary file 11 [file 44318_2026_919_MOESM7_ESM.zip › Figure 1/1B/Figure 1B drop test WT cdc6_1 diff temp.pdf]

25°

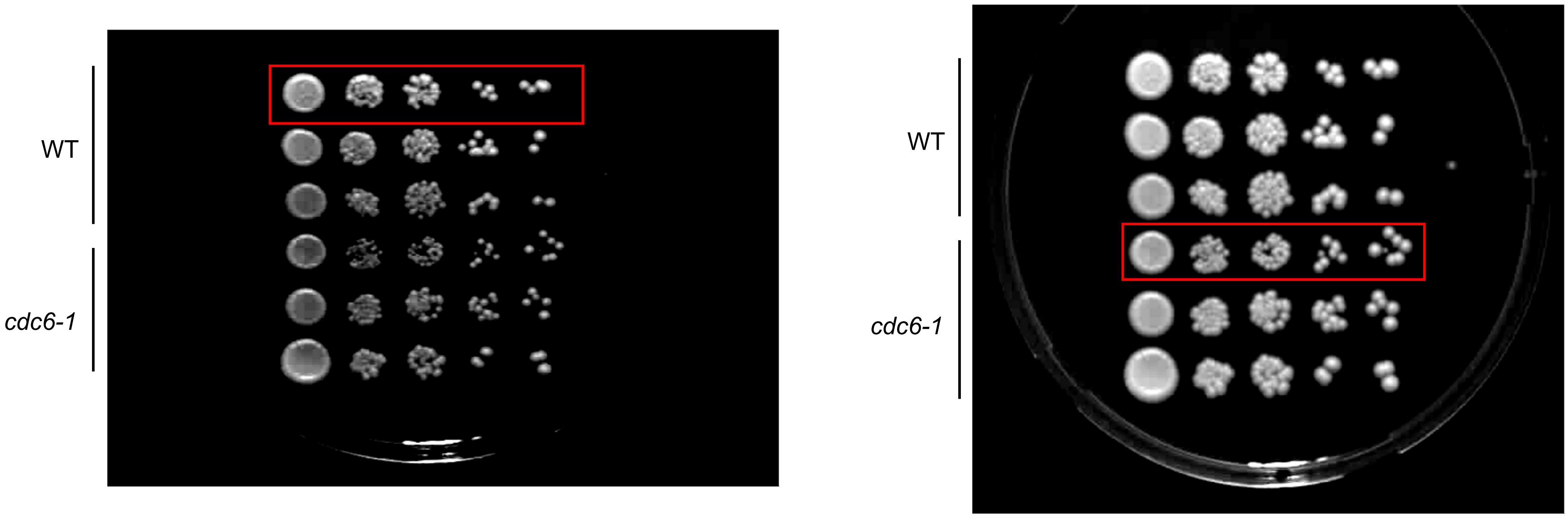

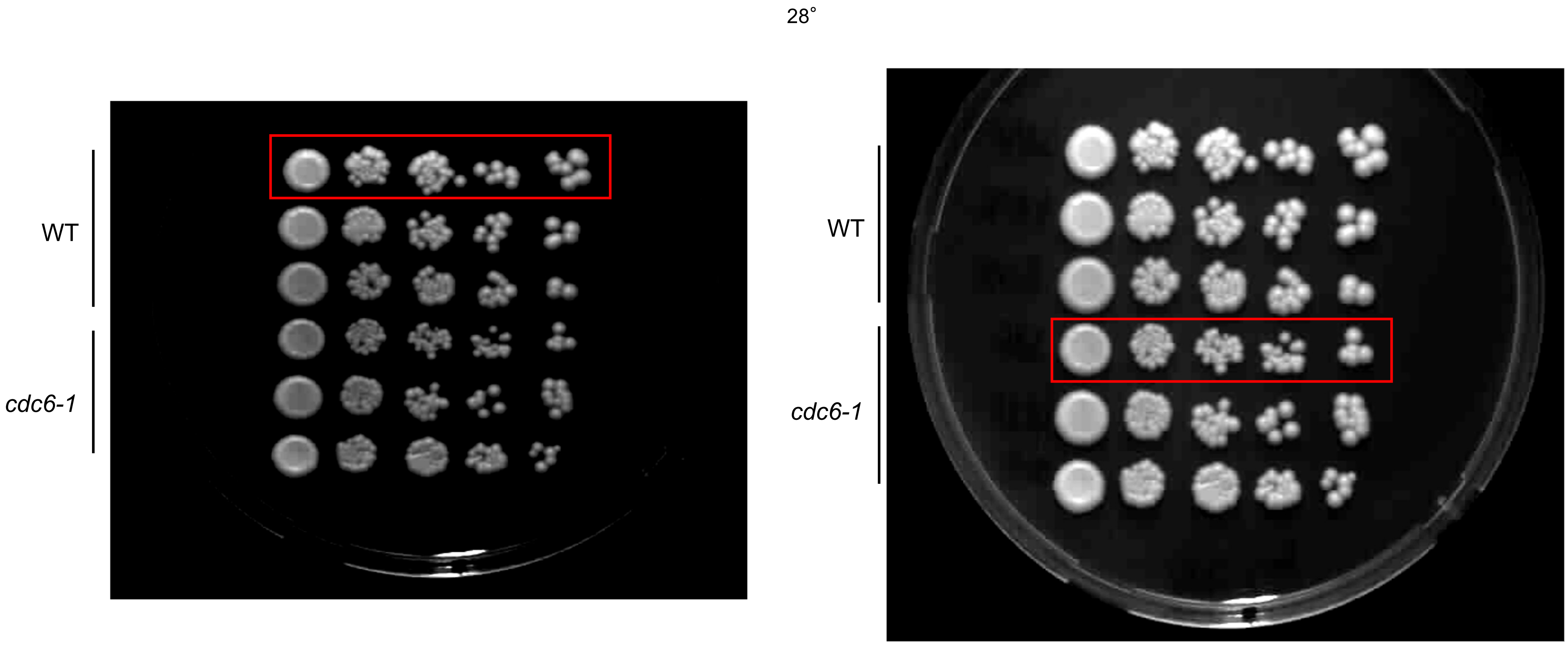
28˚
WT
cdc6-1
WT
cdc6-1

30°

WT

cdc6-1

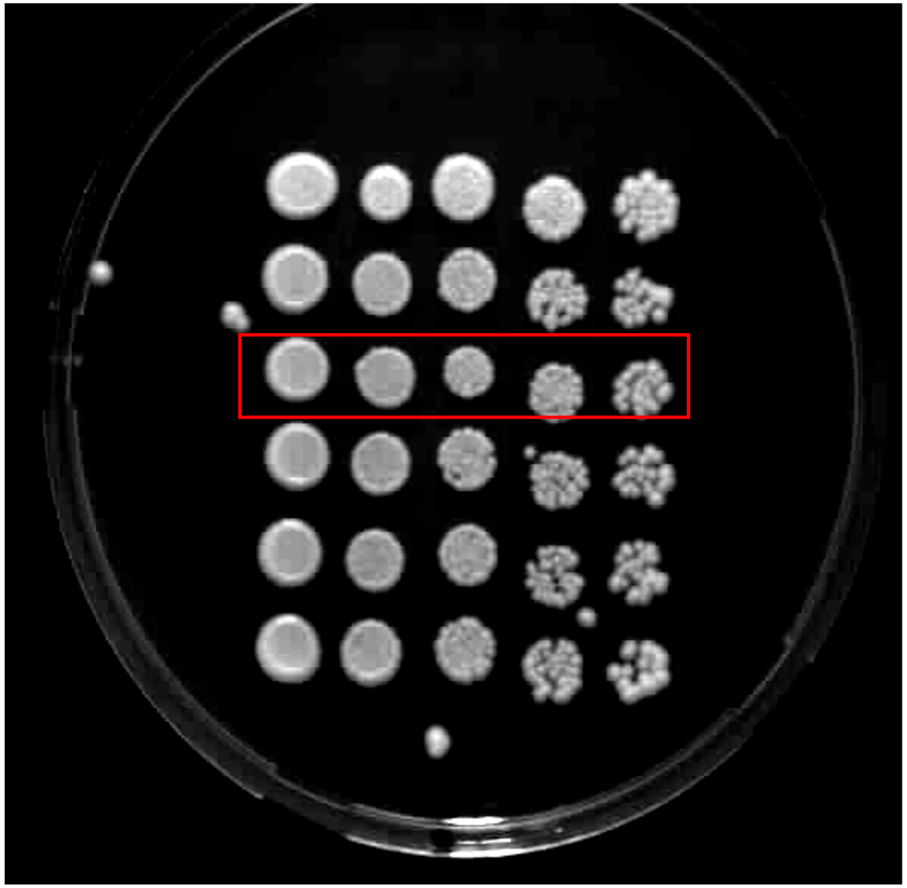

WT

cdc6-1

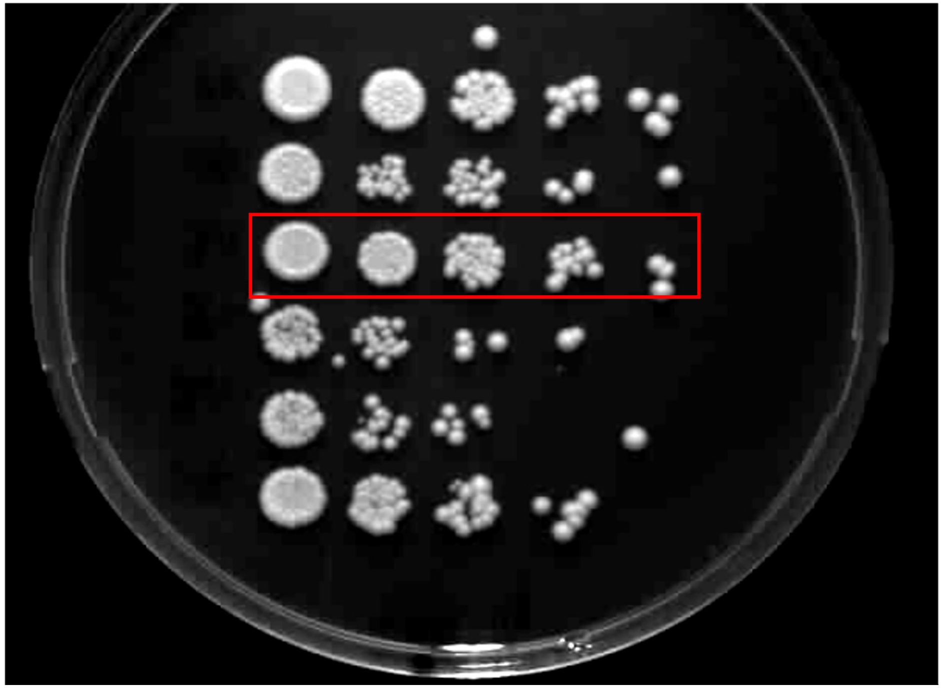

32°

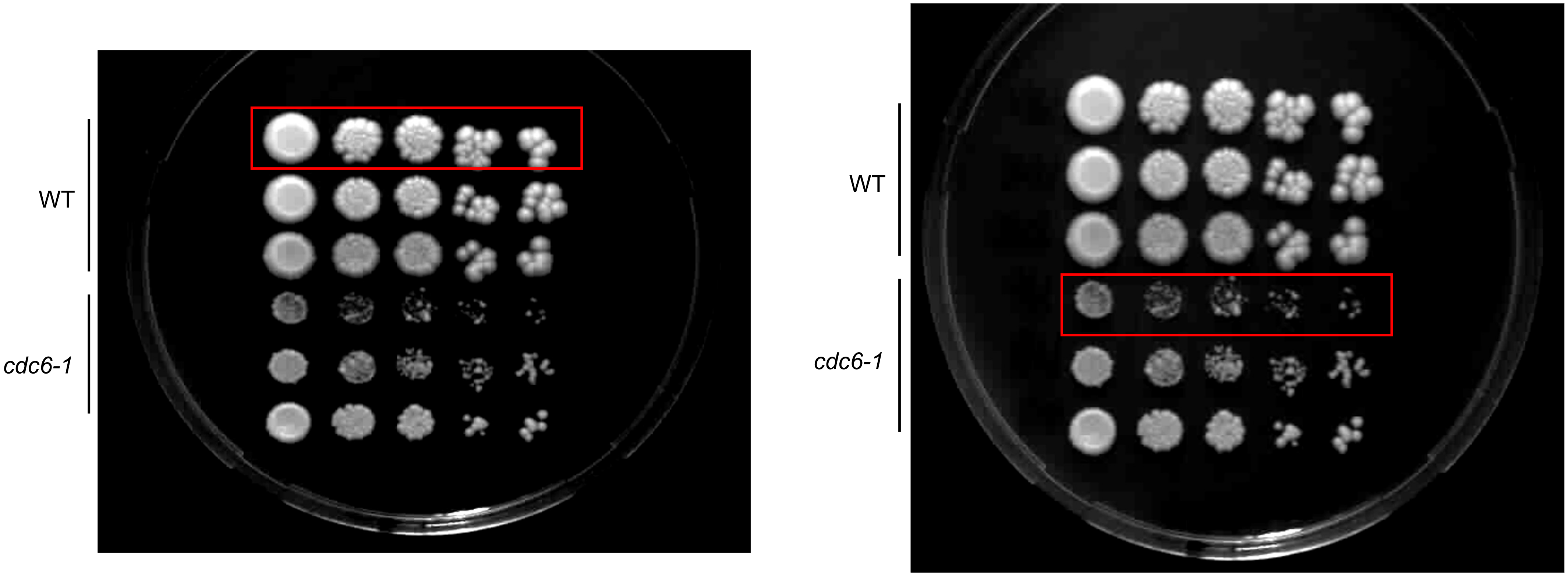

34°

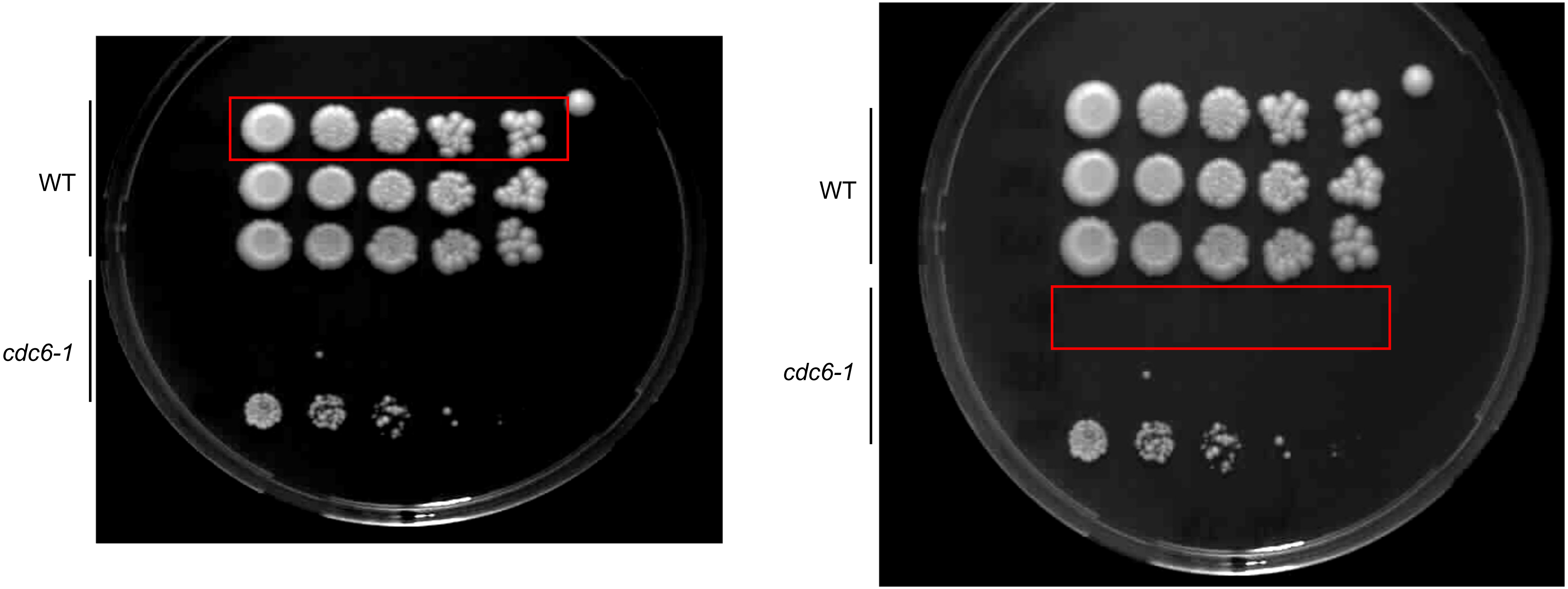

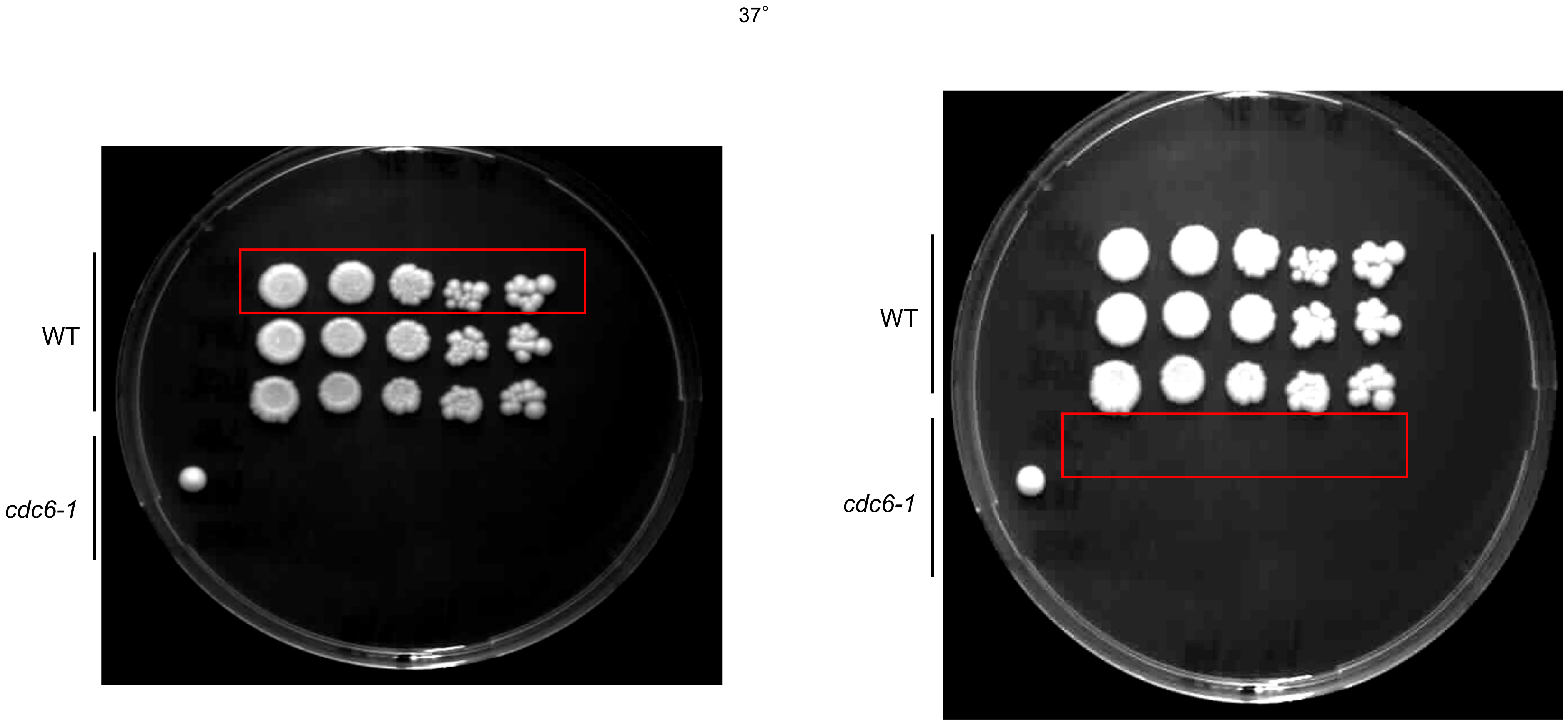
37°
WT
cdc6-1
WT
cdc6-1
