# Supplementary material for: Switch from canonical to recombination-dependent DNA synthesis by M-CDK phosphorylation of DNA Polα
Source: EMBO J. 2026 Oct 10;45(21):7621–46. doi: 10.1038/s44318-026-00919-5 (PMC13728163; doi:10.1038/s44318-026-00919-5)
Supplement: Supplementary file 13 [file 44318_2026_919_MOESM9_ESM.zip › 3E/Comment on the data fig 3E.docx]

Comment on raw data fig 3E

The folder contains the values Juande used to make the panel in a excel sheet named Fig3E JD and an excel sheet containing Riccardo’s data I found named Fig3E RM.

The values Juande used are in the 10^4^ range while Riccardo’s are in the 10^5^ range. Both graphs are otherwise identical. I added both in the folder named Fig3E JD (that in the paper) and Fig3E RM (found in Riccardo’s file).

I don’t know if Riccardo made a mistake in making the math that both Juande and you fixed or if Juande forgot a 0 when he reported the values!

Here is the path to Riccardo’s raw data: ESL$_Riccardo_Recomb Assay
